# Uniform Preparation of (4*R*)- and (4*S*)‑Fluoro‑l‑lysines from One Precursor: Retrosynthesis Software Approach and the Route of a Chemist

**DOI:** 10.1021/acs.joc.5c02332

**Published:** 2025-11-27

**Authors:** Vladimir N. Belov, Christopher Golz

**Affiliations:** † Department of NanoBiophotonics, 28282 Max Planck Institute for Multidisciplinary Sciences (MPI NAT), Am Fassberg 11, Göttingen 37077, Germany; ‡ Facility for Synthetic Chemistry, MPI NAT, Am Fassberg 11, Göttingen 37077, Germany; § 9375 Institut für organische und biomolekulare Chemie der Georg-August-Universität, Tammannstrasse 2, Göttingen 37077, Germany

## Abstract

Fluorine-containing analogues of natural amino acids are becoming increasingly important as constituents of peptides, building blocks for new drugs, and drug candidates. We experimentally verified the synthesis of 4-fluoro-l-lysine proposed by SYNTHIA software, found the step which did not work as predicted, and modified it. Hereby, we found that α-iodoketones may be used in C-alkylation of enolates generated from (chiral) glycine derivatives. Eventually, a successful route was developed by us. It started from *Z*-Asp-OBn and led to a separable mixture of (4*R*)- and (4*S*)-epimers of 4-hydroxylysine, with protected amino groups (α-*Z*, ϵ-Boc) and carboxyl groups (as *tert*-butyl ester). These compounds were obtained from the common precursor (a 4-oxo-l-lysine derivative). Further transformations of each C^4^–OH epimer included the S_N_2 reaction with PyFluor followed by manipulation with protecting groups; they were carried out separately and resulted in (4*R*)- and (4*S*)-fluoro-l-lysine isomers. Orthogonally protected α-Fmoc-ε-Boc-4-fluoro-l-lysines as (4*R*)- and (4*S*)-epimers were prepared as compounds with a set of protecting groups compatible with the conditions of solid-phase peptide synthesis.

## Graphical abstract

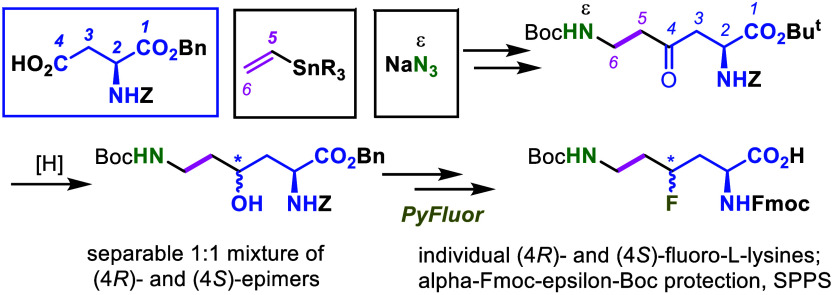

## Introduction

Fluorine-containing analogues of natural amino acids turned to be essential building blocks for the construction of new (cyclic) peptides, drugs, or drug candidates.^1a–1c^ The substitution of hydrogen by fluorine often results in analogues having longer durations and greater levels of activity than their nonfluorinated counterparts. Recently, the ribosomal translation of fluorinated noncanonical amino acids was reported to provide biologically active macrocyclic peptides incorporating these compounds.^1d^ Due to the unique NMR spectroscopic properties and absence in most biosystems, ^19^F allows to study protein folding and dynamics.^1e^ The limited number of fluorine-containing α-amino acids, available commercially or synthetically, demands the development of reliable syntheses of these compounds; in particular, amino acids with one fluorine atom attached to the side chain are the most underrepresented ones.

This report deals with the preparation of both (4*R*)- and (4*S*)-epimers of 4-fluoro-l-lysine with a natural *S*-configuration at C-2 (Scheme 1). In the literature, we found one multistep synthesis (**a**) of orthogonally protected (4*R*)-fluoro-l-lysine (Scheme 1a).^2^ The overall yield over 10 steps was high (28%).^2^ Other relevant routes resulted in 4-hydroxylysine derivatives (precursors to 4-fluorolysine), which were obtained in good yields (Scheme 1) as an inseparable mixture of epimers (**b**)^3a^ or one single (4*R*)-epimer (**c**).^4^ Importantly, routes (**a**)^2^ and (**c**)^4^ are based on asymmetric induction. Without modifications (e.g., introducing appropriate chiral catalysts or new chiral auxiliaries), they are inapplicable to the preparation of (4*S*)-fluoro- or (4*S*)-hydroxylysine derivatives. The route (**b**)^3^ lacks experimental details and is complicated by the spontaneous cyclization of benzyl 4-hydroxylysinate into the mixture of epimeric five-membered lactones (Scheme 1b). The routes (**b**)^3^ and (**c**),^4^ though high-yielding, are “incomplete”, as they do not provide 4-fluoro-l-lysine.

**Scheme 1:**
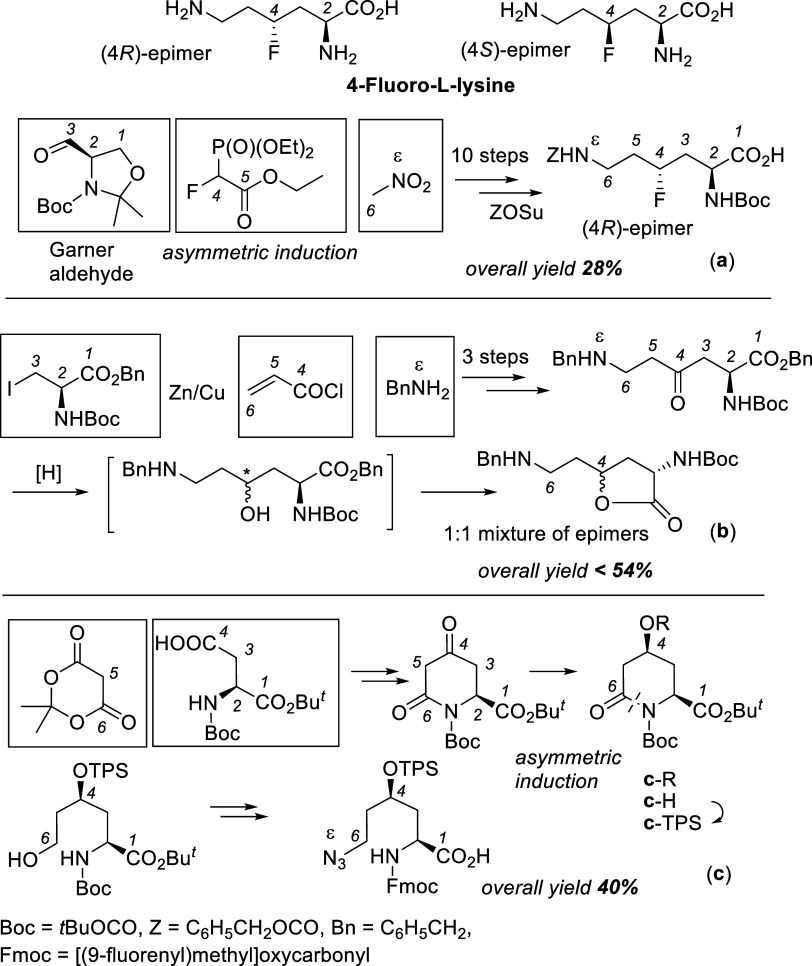
Published Syntheses of (4*R*)-4-Fluorolysine (**a**)^2^ and 4-Hydroxylysine Derivatives (**b**),^3a^ (**c**)^4^ ^a^

a See the text for details.

The literature data (Scheme 1b,c and ref 5) underscore the importance of 4-hydroxy-l-lysine derivatives as intermediates which may be converted into 4-fluoro-l-lysine epimers via nucleophilic substitution of the secondary hydroxyl group (with inversion at C-4).

The synthesis of 4-hydroxy-l-lysine epimers from l-lysine was reported as early as 1965.^5^ This approach was based on the photochlorination of l-lysine in aqueous (aq.) HCl to 4-chloro-l-lysine^5a^ followed by hydrolysis and isolation of only *threo* (4*R*)-hydroxy-l-lysine (as lactone).^5b^ In 5–6 additional synthesis steps, this lactone was converted into the mixture of *threo* (4*R*)- and *erythro* (4*S*)-epimers of 4-hydroxy-l-lysine formed in the ratio of 3:7.^5b^ For separation and isolation of 90 and 230 mg amounts of these epimers, 1.7 L of the Amberlite IR-120 ion exchanger was used. Nowadays, *threo* (4*R*)-hydroxy-l-lysine is produced biochemically with high efficiency and capacity from lysine by using lysine hydroxylases.^6^

Importantly, the ring closure to stable five-membered lactones occurs very readily^3,5^ and “masks” the hydroxyl group at C-4; even if the carboxyl group is protected as benzyl ester.^3^ Therefore, a promising synthesis strategy based on the conversion of this hydroxyl group to fluoride requires stable protection of the carboxyl group (i.e., as *tert*-butyl ester). Taking into account the literature data and the experimental evaluation of the route provided by SYNTHIA software (see the next section), we worked out the new “human-based” route to (4*R*)- and (4*S*)-epimers of 4-fluoro-l-lysine with a natural *S*-configuration at C-2 (Scheme 1). They were prepared from a common precursor (Cbz-Asp-OBn) in the course of a diverging route involving the straightforward separation of two diastereomers of 4-hydroxy-l-lysine with appropriately protected amino and carboxyl groups. Our approach affords both epimers of 4-hydroxy- and 4-fluoro-l-lysines. In this respect, it is beneficial as compared with published syntheses (**a–c**) of 4-fluorolysine and 4-hydroxylysine presented in Scheme 1.

## Results and Discussion

### Retrosynthesis and Evaluation

Before embarking on the syntheses involving the transformation of C-4 hydroxyl to fluoride, we assessed the possible routes to 4-fluorolysine in a broader context based on computer-assisted retrosynthesis. For that, we applied SYNTHIA software.^7^ By using SYNTHIA, the chemist can define search parameters and types of starting materials with respect to their commercial availability, price, patent pending, published or unpublished data, etc. The user can set protecting group preferences, view literature prototypes of the proposed transformations, define stop-search conditions, and filter, sort, compare, and analyze custom pathways by viewing detailed molecule structures or graphs to explore the most cost-effective routes to chemical targets. Scheme 2 contains the route proposed by SYNTHIA (**A** + **B**–X). Halides **B**–X (X = Br, I) were expected to provide stereoselective C-alkylation of enolate generated from the chiral glycine derivative (**A**).^8^ Halides **B**–X (X = Br, I) correspond to the C^3^–C^6^ carbon chain of 4-fluoroglycine with the *N*-phthalimido group (NP) and two vicinal halogen atoms: F at C-4 and X = Br or I at C-3. We found this route reasonable and evaluated its feasibility.^9^

**Scheme 2:**
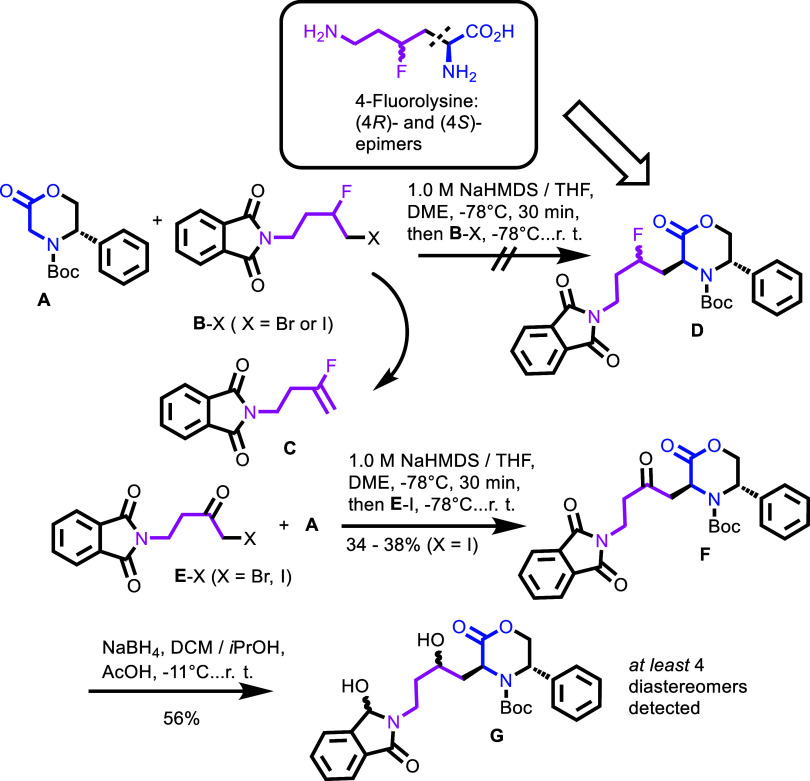
Retrosynthesis Route to 4-Fluorolysine Proposed by SYNTHIA Software^7^ and Based on the Chiral Glycine Equivalent (A) and *N*-(3-Fluoro-4-X-butyl)­phthalimide B–X (X = Br, I)^a^

a The strategic C–C bond in 4-fluorolysine is crossed by a dashed line. The unproductive route (A + B–X) led to the elimination product (C). The modified approach involved iodoketone (E–I); it provided the required intermediate F, but was complicated at the reduction step leading to an inseparable mixture of diastereomers (G).

We prepared vicinal halides **B**–X (X = Br, I, Scheme 2) by “formal” addition of the “X–F reagent” to the alkene PN–(CH_2_)_2_CH=CH_2_ (PN = *N*-phthalimido group). In the prototype Ad_E_ reaction of alkenes, the combination of either the HF–pyridine or the HF–Et_3_N complex with *N*-bromo- or *N*-iodosuccinimide acts as the synthetic equivalent of an “X–F” reagent (X = Br, I).^10^ Flash chromatography followed by recrystallization from cyclohexane removed the undesired gem-dibromides or gem-diiodides, which were formed as byproducts. Unfortunately, the next step toward compound **D** (suggested by the retrosynthesis software)^7^ invariably delivered fluoroalkene **C** (and only **C**) formed from compounds **B**–X upon elimination of HX (X = Br, I). The desired product **D** did not form. The formation of the elimination product **C** makes this synthesis route unproductive (Scheme 1). Post factum, it is easy to explain this result; the higher acidity of the −CHF- proton in the fragment −CHFCH_2_X of compound **B**–X in Scheme 2 favors elimination over nucleophilic substitution.

Eventually, we corrected the plan proposed by SYNTHIA software^7^ (**A** + **B**–X) and replaced vicinal dibromides **B**–X with α-haloketones **E**–X (X= Br, I in Scheme 2). For α-haloketones, the elimination of HX is impossible. Yet another and, in fact, the main reason for the use of α-haloketones was that only highly reactive halides (e.g., benzyl, allyl, RO_2_CCH_2_) undergo C-alkylation of (chiral) glycine equivalents.^11^ In line with that, the reactivity of α-haloketones in S_N_2 reactions is several orders of magnitude higher than that of inactivated alkyl halides. In this event, only α-iodoketone **E**–I provided the required product **F**. The reactivity profile of bromoketone **E**–Br toward compound **A** was different. Thus, the modified approach (**A** + **E**–I) worked, and the desired compound **F** was obtained in a moderate yield.

The susceptibility of the phthalimido group toward NaBH_4_ was expected to be lower than that of ketone,^12^ but the actual outcome of the reduction of compound **F** was difficult to foresee. We observed that the reaction of compound **F** with NaBH_4_ was always accompanied by a “partial” reduction of the phthalimido group, so that only compound(s) **G** could be isolated in useful amounts (as mixtures of at least four diastereomers, as evidenced by NMR spectra). The selectivity was too low, and the desired alcohol with an intact phthalimido group (though detected in the reaction mixture by means of LC-MS) was very difficult to isolate, as it formed in appreciable amounts only when the degree of conversion of the starting material (ketone **F**) was low. Interestingly, the mild method of amine deprotection based on the “partial” reduction of the phthalimido group followed by mild acidic hydrolysis was reported.^13^ We did not pursue this route any further because compound **G** was expected to undergo nucleophilic substitution of hydroxyl groups with fluorine on both sites (C-4 and CH­(OH)N in phthalimide).

The important result of this part of the work is that α-iodoketone (as strong) electrophiles may be used in C-alkylation of enolates generated from (chiral) glycine derivatives and probably related structures. This conclusion is new because up to now, the reactivity of α-haloketones toward carbanions was not studied.

### Synthesis, Separation, and Structure Elucidation of *(*4*S)*- and *(*4*R)*-Fluoro-l-lysine Derivatives

After failing to prepare the target structure according to the route proposed by SYNTHIA software, we developed our synthesis of two epimers of 4-fluoro-l-lysine from the common precursor *Z*-Asp-OBn (**1**), as given in Scheme 3. The main assumption was that the suitably protected 4-hydroxylysines could be converted into the corresponding fluorides via the S_N_2 reaction (with inversion of the configuration at C-4).

**Scheme 3:**
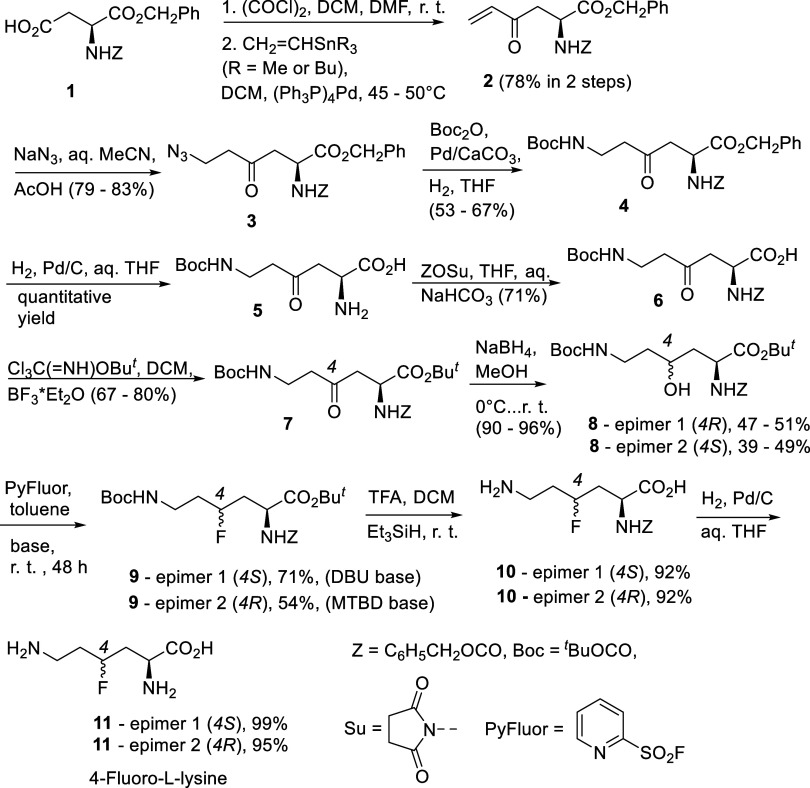
Synthesis of Two Epimers of 4-Fluoro-l-lysine from the Common Precursor *Z*-Asp-OBn^a^

a Reduction of ketone **7** gives a separable mixture of two diastereomeric alcohols **8**. Transformations **8** → **9** → **10** → **11** were carried out for each epimer separately. The absolute configuration (4*S*) of compound **9**–epimer 1 was established by X-ray analysis of the product obtained from it (compound **13**–epimer 1) (Scheme 4).

The report published by Fülöp et al.^14^ confirmed the feasibility of OH to F conversion for (cyclic) structures containing allylic alcohol and N-Boc groups (urethane protection).

The disconnection approach to the 4-fluorolysine structure is given in the TOC graph. The lysine side chain [H_2_N­(CH_2_)_4_] is two carbon atoms longer than that of asparagine [HO_2_CCH_2_]. If we consider the linear extension of the asparagine side chain, then the position of the carbonyl group in HOOCCH_2_ corresponds to C-4 in lysine. Thus, the objective was to start from the suitably protected asparagine derivative and simply “add” a two-carbon chain (with a surrogate of the terminal amino group) to the carbonyl function of the asparagine side chain. This objective is easy to match if we consider Stille enone synthesis, which involves acid chlorides RCOCl and CH_2_=CHSnR_3_ and affords ketones RCOCH=CH_2_.^15^ This transformation works also for highly functionalized and complex structures.^16^ Having in mind these facts, we started from commercially available and affordable Z-Asp-OBn (compound **1** in Scheme 3) and converted it to a stable acid chloride using the published protocol.^17^ The following Pd(0)-catalyzed reaction with CH_2_=CHSnR_3_ (R = Me, *n*Bu) afforded enone **2** with the required number of carbon atoms (4) in the side chain. The terminal amino group of lysine was then introduced (as azide) when compound **2** reacted with HN_3_ generated from sodium azide and acetic acid^18^ in aq. MeCN. The direct reduction of the azide group in compound **3** into amine is likely to lead to the seven-membered amide formed upon cyclization involving the ester group. We observed this kind of spontaneous and very rapid reaction readily occurring at room temperature between methyl ester (C-1) and the primary amino group attached to C-7 in cyclopropanated amino acid derivatives.^19^ Fortunately, it is possible to capture the amino group and transform it into urethane if the azide reduction is carried out in the presence of *tert*-butyl pyrocarbonate.^20^ However, azide **3** in Scheme 3 cannot be directly converted into N-Boc-protected amino acid **5**. This transformation was carried out in 2 steps: first by hydrogenolysis over the Lindlar catalyst, which does not cleave benzyl ester and *N*-benzyloxycarbonyl groups,^21^ and then, upon isolation of intermediate **4**, by hydrogenolysis with “normal”, highly active Pd on charcoal. Compound **5** was then transformed into *tert-*butyl ester **7**. *tert-*Butyl ester is stable against nucleophiles, and its presence prevents the formation of five-membered lactone, which is likely to occur upon the envisaged reduction of the carbonyl group in ester **7**. For that, it was necessary first to protect the α-amino group in compound **5** and obtain bis-N-protected diamino acid **6**. This step (**5** → **6**) was carried out by using *N*-(benzyloxycarbonyl)­oxy succinimide (ZOSu; Scheme 3),^22^ which is superior to Z-Cl. *tert*-Butyl ester **7** was prepared by employing *tert*-butyl trichloroacetimidate.^23^ Compounds **3**–**7** represent 4-oxo-l-lysine equivalents with protected functionalities; azide in compound **3** is a surrogate for the terminal amino group (compound **3**). Reduction of the carbonyl group in compound **7** with sodium borohydride in methanol was selective (ester and urethane groups were not involved) and led to a 1:1 mixture of two epimers of alcohol **8**. Epimer 1 of alcohol **8** has a higher retention factor (*R*_f_), while epimer 2 has a lower *R*_f_ value. Remarkably, with the given set of protecting groups in structure **8**, isomers were separable by flash chromatography on regular silica gel. This feature makes the synthesis of individual epimers of 4-fluorolysine possible from one common precursor and via one branching route. Starting from compound **8**, all subsequent steps in Schemes 3 and 4 were performed separately (for individual epimers). The substitution of the hydroxyl group with fluorine was found to be feasible by applying the PyFluor reagent in toluene and in the presence of DBU or MTBD as strong organic bases.^24^ Under conditions reported in ref 14 (1.3 equiv of DAST without a base), we observed no deoxyfluorination, but only the formation of five-membered urethane formed upon cleavage of the acid-sensitive N-Boc group (from epimer 1 of compound **8**) and cyclization of isocyanate (formed upon elimination of *t*BuOH) involving the hydroxyl residue at C-4. *tert*-Butyl ester and N-Boc groups in fluorides **9** were cleaved in one run (TFA in DCM with Et_3_SiH),^25^ and α-*N*-Z-4-fluorolysines **10** were isolated by flash chromatography on the reversed phase (RP-C18).

**Scheme 4:**
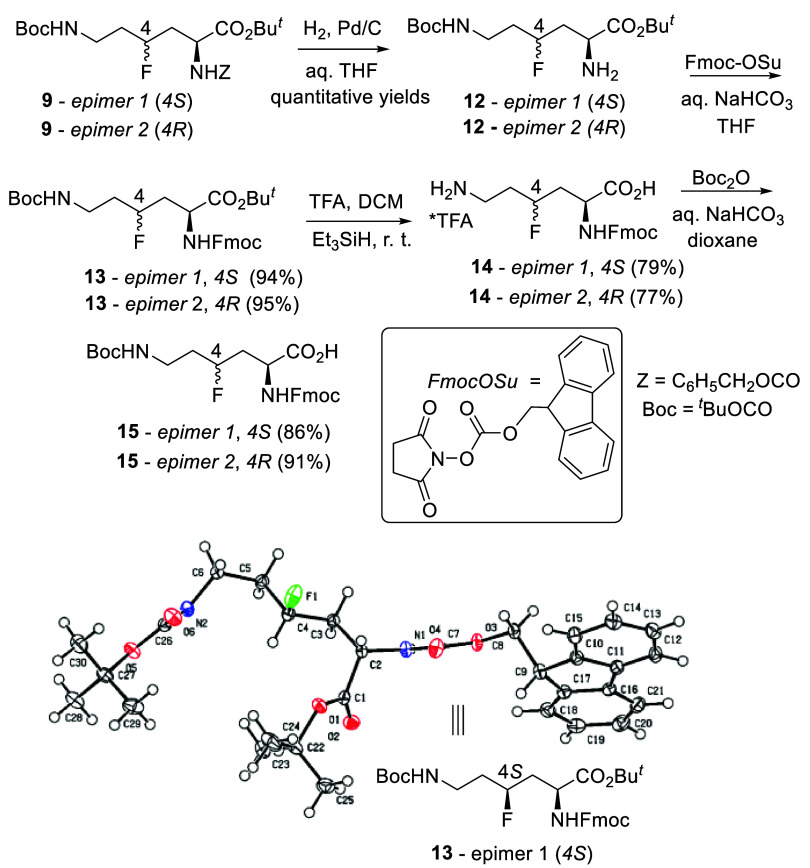
Synthesis of Orthogonally Protected (α-Fmoc-ε-Boc) 4-Fluoro-l-lysine Epimers 4*S* (Epimer 1) and 4*R* (Epimer 2)^a^

a Transformations **9** → **12** → **13** → **14** → **15** were carried out for each epimer separately. The absolute configuration (4*S*) of compound **13**–epimer 1 was established by X-ray analysis of the sample recrystallized from acetonitrile.

4-Fluorolysine epimers (**11** in Scheme 3) were obtained by hydrogenolytic deprotection of compounds **10** in aq. THF over Pd/C followed by lyophilization. Over 10 steps, the yield of the (4*S*)-epimer was found to be 5.7% and the yield of the (4*S*)-epimer was found to be 3.7%. Thus, the sum of epimers was prepared with a 9% overall yield and an average yield of 78.6% in one step (0.786^10^ = 0.09).

The transformations shown in Scheme 4 were performed for two reasons. One objective was to prepare a crystalline derivative suitable for X-ray analysis in order to determine the absolute configuration at C-4 at least for one compound. Compound **13**–epimer 1 (Scheme 4) contained the Fmoc residue, and it readily crystallized from acetonitrile in prisms (plates) melting at 171–173 °C. According to X-ray analysis, in this compound, the configuration at C-4 was *S* (Scheme 4). Thus, the absolute (4*S*)-configuration was attributed to epimers 1 of compounds **9–12** as precursors of **13**–epimer 1. We assume that inversion of the configuration at C-4 takes place upon conversion of secondary alcohols **8** into fluorides **9**. Thus, we attribute the (4*R*)-configuration to compound **8**–epimer 1 and the (4*S*)-configuration to compound **8**–epimer 2. This enabled to establish the configuration at C-4 for all epimers in the whole series of compounds **8**–**15** (Schemes 3 and 4).

Another objective was to prepare individual epimers of 4-fluoro-l-lysine with orthogonally protected amino groups (α-Fmoc and ε-Boc), suitable for solid-phase peptide synthesis. This goal was achieved by performing the four-step syntheses shown in Scheme 4. The overall yields were high (64–67%). Though all of these transformations are straightforward and trivial, as they involve standard protocols for *N*-Cbz, *N*-Fmoc, *t*-butyl ester, and *N*-Boc cleavage and introduction,^22,25^ they do have only one direct literature prototype for the natural amino acid lysine.^26^

## Conclusion

We prepared both epimers of 4-fluoro-l-lysine (with a natural *S*-configuration at C-2) starting from the asparagine derivative Z-Asp-OBn (**1** in Scheme 3)—a commercially available compound affordable on a multigram scale. The synthesis scheme was elaborated and realized after considering the literature data (see the Introduction section) and experimental evaluation of the approach proposed by the synthesis software (Scheme 2).^7^ As a result, we developed a synthesis (Schemes 3 and 4) based on reliable transformations, well established for amino acids and organic chemistry in general. The separation of epimers at C-4 was realized for 4-hydroxy-l-lysines with common protecting groups (compound **8** in Scheme 3); without the necessity to screen and choose other derivatives. The access to orthogonally protected α-Fmoc-ε-Boc-4-fluoro-l-lysine epimers (4*S* and 4*R* in Scheme 4) affords their use in automated peptide synthesis (on the solid phase) with a standard set of protecting groups. The absence of preparative HPLC separations throughout the syntheses facilitates upscaling.

Evaluating the synthesis route proposed by SYNTHIA software, we found that iodoketones may be used in C-alkylation of enolates generated from (chiral) glycine derivatives and probably related structures. This conclusion is new because up to now, the reactivity of α-haloketones toward carbanions was not studied. In the future, we are going to study the reactions of α-bromo and α-iodoketones with enolates generated from (chiral) glycine derivatives and related structures.

## Experimental Section

### General Remarks

Anhydrous solvents were stored in molecular sieves. The reactions were performed with magnetic stirring under argon. The temperature 0 °C corresponds to the cooling of the reaction mixture with an ice bath. Oil baths were used for heating the reaction mixtures, and the bath temperatures are given as the reaction temperatures. Evaporations in vacuo were performed in a rotary evaporator with a water-bath temperature not exceeding 45 °C (unless stated otherwise). Hydrogenation reactions were performed with prereduced Pd/C (10% Pd, oxidized form) in Schlenk flasks (stopped with septa) at ambient pressure and under vigorous stirring (500–1200 rpm). Organic solutions of reaction products were dried over anhydrous Na_2_SO_4_ prior to evaporation in vacuo. Automated flash column chromatography was carried out using cartridges with spherical silica gel from Biotage on a Biotage Isolera device. Reversed phase flash column chromatography was carried out on POLYGREP 60–50 C18 (MACHEREY-NAGEL GmbH & Co. KG) in columns packed manually. For analytical TLC, Merck Millipore ready-to-use plates with silica gel 60 (F_254_) were used. The spots were visualized by illumination with a UV lamp (λ = 254 nm) and staining with phosphomolybdic acid or ninhydrin solutions. Analytical TLC (reversed phase) was performed on Merck SiO_2_ 60-RP-C18 plates (F254S), with the possibility of UV detection (254 nm). The spots were detected by heating with a “heat gun”. ^1^H, ^13^C, and ^19^F NMR spectra were recorded at 25 °C on an Agilent 400-MR system (400 MHz ^1^H, 100.5 MHz ^13^C, and 376 MHz ^19^F). The NMR spectra are referenced to tetramethylsilane (TMS, δ = 0 ppm) using the signals of residual protons or ^13^C nuclei for CDCl_3_ (^1^H 7.26, ^13^C 77.4 ppm), CD_3_OD (^1^H 3.34, ^13^C 49.9 ppm), and CD_3_CN (^1^H 1.96, 1.76 ^13^C). Multiplicities of signals are described as follows: s = singlet, d = doublet, t = triplet, q = quartet, dd = double of doublets, m = multiplet or overlap of nonequivalent resonances, *m*_c_ = centrosymmetric multiplet, dm = clearly resolved doublet of multiplets, and br. = broad. *J* values are given in Hz. Analytical (HP)­LC-MS analyses were performed on a Phenomenex analytical column, Kinetex C18, 2.6 μm, 75 × 3 mm, flow 0.5 mL/min, with a Thermo Fisher Scientific ISQ EM mass spectrometer (coupled to an Ultimate 3000 system). Gradient: 20–100% v/v acetonitrile (+0.1% v/v HCOOH) in H_2_O (0.1% v/v HCO_2_H) over 7 min (unless stated otherwise). ESI-HRMS spectra were measured on a MICROTOF spectrometer (Bruker) equipped with an Apollo ion source and a direct injector with an LC-autosampler Agilent RR 1200 instrument (Institut für Organische and Biomolekulare Chemie, Georg-August-Universität Göttingen). Single-crystal X-ray diffraction analysis was carried out. Data collection was done on a two dual-source equipped Bruker D8 Venture four-circle diffractometer from Bruker AXS GmbH. Used X-ray sources: microfocus IμS 2.0 Cu/Mo from Incoatec GmbH with mirror optics HELIOS and a single-hole collimator from Bruker AXS GmbH; used detector: Photon III CE14 from Bruker AXS GmbH; used programs: APEX6 Suite (v2024.9–1) for data collection and therein integrated programs SAINT V8.41A (integration) and SADABS 2016/2 (absorption correction) from Bruker AXS GmbH; structure solution was done with SHELXT; refinement with SHELXL-2018/3.^27^ OLEX2 and FinalCif were used for data finalization.^28^

### Compound **2**

(*S*)-CH_2_=CHCOCH_2_CH­(NHCO_2_CH_2_C_6_H_5_)­CO_2_CH_2_C_6_H_5_. Z-Asp-OBn (**1**) was converted to acid chloride according to the published method.^17^ For that, compound **1** (5.00 g, 14 mmol) was dissolved in anhydrous DCM (50–60 mL), the solution was cooled to 0 °C in an ice bath, and 1.20 mL (1.78 g, 14 mmol) of freshly distilled (COCl)_2_ dissolved in ca. 10 mL of DCM was added via a syringe into the stirred solution of the starting material. Then, 2 drops of anhydrous DMF were added, and the cooling bath was removed. The evolution of gas was observed in the bubble counter. After the reaction mixture warmed up to room temperature and the gas evolution ceased (it took about 2 h), all volatile materials were removed in vacuo, and the residue solidified. The colorless solid residue of acid chloride was dissolved in DCM (40–50 mL) and converted into compound **2** according to the (modified) published procedure.^29^ For that, neat Bu_3_SnCH=CH_2_ (4.84 g, 14 mmol) was added to the solution of acid chloride in DCM followed by Pd­[Ph_3_P]_4_ (210 mg, 0.2 mmol). The reaction mixture was stirred at 45–50 °C for 4.5 h, diluted with CHCl_3_ (200 mL), and washed with sat. NaF solution (100 mL). The organic layer with the precipitate was separated and filtered, and the precipitate was washed with CHCl_3_. Combined organic solutions were washed with saturated brine and dried. The solvents were removed in vacuo, and the residue was subjected to flash chromatography on SiO_2_ (50 g cartridge). Elution with 5–75% EtOAc in hexane (over 12 column volumes) afforded Bu_3_SnOH first and then compound **2** as a slowly crystallizing oil (3.92 g, 78%), which was obtained after evaporation of the pooled fractions and keeping the residue in vacuo; mp 49–50 °C, colorless solid. [α] = +18 (*c* = 1.2, CHCl_3_). Alternatively, Me_3_SnCH=CH_2_ (prepared from CH_2_=CHBrMg and Me_3_SnCl)^30^ was used instead of Bu_3_SnCH=CH_2_. In this case, Me_3_SnOH (soluble in water) was removed by aq. workup. ^1^H NMR (400 MHz, CDCl_3_) δ = 7.43–7.25 m (10H, H^ar^), 6.32 dd (1H, *J* = 17.7 and 10.2 Hz, H-5), 6.22 dd (1H, *J* = 17.8 and 1.3 Hz, H-6), 5.90 dd (1H, *J* = 18.1 and 4.4 Hz, H-6), 5.84 br.d (1H, *J* = 8.7 Hz, NH), 5.18/5.17 (2H, AB-system, CH^A^H^B^O, *J* = 12.3 Hz), 5.12 s (2H, CH_2_O), 4.70 dt (1H, *J* = 8.6 and 4.3 Hz, H-2), 3.39 dd (1H, *J* = 18.1 and 4.4 Hz, H-3a), 3.18 dd (1H, *J* = 18.1 and 4.2 Hz, H-3b) ppm. ^13^C­{^1^H} NMR (101 MHz, CDCl_3_) δ = 198.0 (CO), 170.8 (COO), 156.0 (NC=O), 136.2 (C_q_), 135.9 (=C^5^H), 135.3 (C_q_), 129.7 (=C^6^H_2_), 128.6 (CH), 128.5 (CH), 128.4 (CH), 128.19 (CH), 128.17 (CH), 128.0 (CH), 67.5 (CH_2_O), 67.1 (CH_2_O), 50.0 (C^2^HNH), 41.3 (C^3^H_2_) ppm. HRMS (ESI-TOF) *m*/*z*: [M + Na]^+^ calcd for C_21_H_21_NO_5_Na 390.1312; found 390.1307.

### Compound **3**

(*S*)-N_3_(CH_2_)_2_COCH_2_CH­(NHCO_2_CH_2_C_6_H_5_)­CO_2_CH_2_C_6_H_5_. Vinyl ketone **2** (3.77 g, 10.2 mmol) was suspended in MeCN (25 mL), and the solution of 900 mg of NaN_3_ (13.8 mmol) in H_2_O (3.0 mL) followed by AcOH (2.3 mL) was added with stirring at 0 °C. The reaction mixture was warmed up to room temperature and stirred for 2 h. TLC (hexane/AcOEt, 2/1) indicated full conversion to the product with a slightly lower *R*_f_ than that of the starting material. The reaction mixture was diluted with AcOEt (250 mL), and sat. aq. NaHCO_3_ and solid NaHCO_3_ were added carefully under vigorous stirring until the evolution of CO_2_ ceased and the organic layer separated. Removal of the solvent in vacuo followed by application onto SiO_2_ (25 g cartridge) in warm DCM and elution with 5–80% EtOAc in a 3/1 mixture of cyclohexane/DCM afforded 3.34 g (79%) of a slowly crystallizing yellowish solid; mp 46–47 °C. [α]_25_^D^ = +14 (*c* = 1.1, CHCl_3_). ^1^H NMR (400 MHz, CD_3_OD) δ = 7.40–7.21 m (10HH^ar^), 5.16/5.13 (2H, AB-system, CH^A^H^B^O, *J* = 12 Hz), 5.08 s (2H, CH_2_O), 4.65 dt (1H, *J* = 6.8 and 5.4 Hz, H-2), 3.46 td (2H, *J* = 6.4 and 2.6 Hz, C^6^H_2_N_3_), 3.06 dd (1H, *J* = 17.7 and 5.4 Hz, H-3a), 2.97 dd (2H, *J* = 17.7 and 6.7 Hz, H-3b), 2.70 td (2H, *J* = 6.3 and 1.4 Hz, C^5^H_2_) ppm. ^13^C­{^1^H} NMR (101 MHz, CD_3_OD) δ = 206.8 (CO), 172.8 (COO), 158.4 (NHCOO), 138.1 (C_q_), 137.1 (C_q_), 129.6 (CH), 129.5 (CH), 129.3 (CH), 129.2 (CH), 129.0 (CH), 128.8 (CH), 68.2 (CH_2_O), 67.7 (CH_2_O), 51.2 (C^2^HNH), 46.7 (C^6^H_2_), 44.9 (C^3^H_2_), 42.4 (C^5^H_2_) ppm. LC-MS: the substance is unstable in an acidic aq. gradient. HRMS (ESI-TOF) *m*/*z*: [M + Na]^+^ calcd for C_21_H_22_N_4_O_5_Na 433.1482; found 433.1479.

### Compound **4**

(*S*)-BocNH­(CH_2_)_2_COCH_2_CH­(NHCO_2_CH_2_C_6_H_5_)­CO_2_CH_2_C_6_H_5_. The suspension of Pd/CaCO_3_ (Fluka, 10% Pd; 1.2 g) in THF (30 mL) was vigorously stirred under nitrogen (5 min), and then under hydrogen (15–20 min) in a Schlenk flask (250 mL) stopped with a septum. Azide **3** (1.71 g, 4.16 mmol) and Boc_2_O (1.02 g, 4.68 mmol) were dissolved in THF (20 mL), and this solution was introduced via a syringe into the flask with a stirred suspension of the prereduced catalyst in THF. The reaction mixture was vigorously (900 rpm) stirred at room temperature. The course of the reaction was monitored by TLC (hexane/EtOAc, 2/1). The product has a lower *R*_f_. The reaction was completed in 2 h. The reaction mixture was flushed with nitrogen, the catalyst was filtered off through Celite, and the filtrate was evaporated in vacuo. The residue was subjected to flash chromatography on SiO_2_ (25 g). Elution with a gradient of EtOAc (10–90% v/v) in a DCM/cyclohexane (1:1) mixture followed by pooling and evaporation of the fractions afforded the material, which was subjected to a second separation on a new cartridge under the same conditions. A yield of 1.08 g (53%) of ester **4** as a glasslike foam slowly crystallizing into a colorless solid was obtained upon drying under vacuum; mp 86–87 °C. In another run, 2.90 g (7.07 mmol) of azide **3**, 2.90 g (13.3 mmol) of Boc_2_O, and prereduced 1.97 g of Pd/CaCO_3_ in THF (30 mL) were used. An exothermic reaction was observed, which was completed in 3 h (TLC, hexane/EtOAc, 7/3). Flash chromatography on 50 g of SiO_2_ afforded 2.30 g (67%) of ester **4**. The product should be free from Boc_2_O, as Boc_2_O will react on the next step, when amino acid **5** with the free α-amino group is formed. [α]_25_^D^ = +6.0 (*c* = 1.5, CHCl_3_). ^1^H NMR (400 MHz, CDCl_3_) δ = 7.34 m (10H, H^ar^), 5.79 d (1H, *J* = 8.5 Hz, NH), 5.20/5.14 (2H, AB-system, CH^A^H^B^O, *J* = 12.2 Hz), 5.11 s (2H, PhCH_2_O), 4.88 br. s (1H, NHCO), 4.62 dt (1H, *J* = 8.8 and 4.5 Hz, H-2), 3.29 q (2H, *J* = 6.0 Hz, C^6^H_2_N), 3.15 dd (1H, *J* = 18.0 and 4.6 Hz, C^3^H^A^ H ^B^CO), 2.97 dd (1H, *J* = 18.0 and 4.2 Hz, C^3^ H ^A^H^B^CO), 2.58 t (2H, *J* = 5.9 Hz, C^5^H_2_), 1.43 s (9H, Me_3_CO) ppm. ^1^H NMR (400 MHz, CD_3_OD): δ = 7.39–7.23 m (10H, H^ar^), 6.47 br. s (1H, NHCO), 5.14/5.12 (2H, AB-system, CH^A^H^B^O, *J* = 12 Hz), 5.08/5.05 (2H, AB-system, CH^C^H^D^O, *J* = 12 Hz), 4.61 dd (1H, *J* = 6.7 and 5.2 Hz, H-2), 3.23 q (2H, *J* = 6.4 and 2.6 Hz, C^6^H_2_N), 2.99 m (2H, *J* = 12.9 and 5.9 Hz, H-3), 2.60 m (2H, *J* = 6.3 Hz, C^5^H_2_), 1.40 s (9H, Me_3_CO) ppm. ^13^C NMR­{^1^H} (101 MHz, CD_3_OD) δ = 207.8 (CO), 172.9 (COO), 158.4 (NHCOO), 138.1 (C_q_), 137.1 (C_q_), 129.6 (2 × CH), 129.5 (CH), 129.3 (CH), 129.2 (CH), 129.0 (CH), 128.8 (2 × CH), 80.2 (C_q_O), 68.1 (CH_2_O), 67.7 (CH_2_O), 51.2 (C^2^HNH), 44.7 (C^3^H_2_), 43.6 (C^5^H_2_), 36.3 (C^6^H_2_), 28.7 (3 × Me) ppm. HRMS (ESI-TOF) *m*/*z*: [M + Na]^+^ calcd for C_26_H_32_N_2_O_7_Na 507.2102; found 507.2100.

### Compound **5**

(*S*)-BocNH­(CH_2_)_2_COCH_2_CH­(NH_2_)­CO_2_H. Pd/C (1.0 g; MERCK, 10% Pd, oxidized form) was suspended in a THF/water mixture (4:1, 80 mL), flushed with nitrogen and then with hydrogen, and the suspension was vigorously stirred (1000 rpm) at room temperature for 15 min. Ester **4** (2.30 g, 4.75 mmol) was dissolved in THF (60–70 mL), water was added gradually until the solution became slightly turbid, and this solution was injected via a syringe into the flask with the vigorously stirred suspension of the catalyst. The course of the reaction was monitored by TLC on the normal phase for the educt **4** and TLC on the reversed phase (RP-C_18_) for detecting the final compound and intermediates. Eluent for RP-C_18_ TLC plates: 25% v/v CH_3_CN in 50 mmol of Et_3_N–HCO_2_H buffer (pH ∼ 5–6). The spots on the plates were developed by heating, after applying the ninhydrin reagent. The *R*_f_ of amino acid **5** is ca. 0.4. If the product precipitated on the catalyst, water and THF were added to the reaction mixture intermittently via a syringe (through septum). Gentle heating may be applied for the dissolution of the product. When the reaction was completed (one spot of the product on TLC), the reaction mixture was flushed with nitrogen, the catalyst was filtered off through Celite, and the filter cake was washed with water and THF, until TLC of the filtrate displayed no product. The filtrate was concentrated in vacuum (THF removed), filtered, and lyophilized. Amino acid **5** was thus isolated as a voluminous colorless powder (1.23 g, 100%); mp 128–129 °C. The crystalline sample was obtained by heating a part of this material with DCM–EtOAc to provide a colorless solid decomposing (upon drying) above 144 °C. [α]_25_^D^ = −16.0 (*c* = 0.95, MeOH). ^1^H NMR (400 MHz, D_2_O) δ = 4.02 dd (1H, *J* = 7.3 and 4.1 Hz, H-2), 3.33 t (2H, *J* = 6.3 Hz, C^6^H_2_N), 3.23 dd (1H, *J* = 19.1 and 4.1 Hz, H-3a), 3.15 dd (1H, *J* = 19.1 and 7.3 Hz, H-3b), 2.77 m (2H, C^5^H_2_), 1.42 s (9H, Me_3_CO) ppm. ^13^C­{^1^H} NMR (101 MHz, D_2_O) δ = 210.0 (CO), 158.1 (COO), 81.5 (C_q_O), 50.1 (C^2^HNH), 42.19/42.13 (C^3^H_2_/C^5^H_2_), 34.8 (C^6^H_2_), 27.7 (3 × Me) ppm. HRMS (ESI-TOF) *m*/*z*: [M + H]^+^ calcd for C_11_H_21_N_2_O_5_ [M + H]^+^ 261.1445; found 261.1444.

### Compound **6**

(*S*)-BocNH­(CH_2_)_2_COCH_2_CH­(NHCO_2_CH_2_C_6_H_5_)­CO_2_H. Amino acid **5** (1.14 g, 4.38 mmol) and ZOSu (1.09 g, 4.38 mmol) were dissolved in a THF (60 mL) and water (30 mL) mixture; NaHCO_3_ (440 mg, 5.24 mmol) was added, and the (two-phase) reaction mixture was stirred at room temperature overnight. The course of the reaction was monitored by TLC on the RP-C_18_ plates. Eluent for RP-C_18_ TLC plates: 25% v/v CH_3_CN in 50 mmol of Et_3_N–HCO_2_H buffer (pH ∼ 5–6). The spots on the plates were developed by heating, after application of the ninhydrin reagent. The *R*_f_ of acid **6** is lower than that of compound **5**. When the reaction was completed, the mixture was diluted with water (20 mL) and ether (100 mL), and the aq. layer was separated and extracted with ether (50 mL). Combined organic solutions were extracted with water (25 mL), and then combined aq. solutions (having pH ∼ 7.8) were acidified with 1 M aq. KHSO_4_ to pH ∼ 2, saturated with NaCl, and extracted with CHCl_3_ (2 × 30 mL). After drying, organic solutions were evaporated in vacuo to afford 1.22 g (71%) of acid **6** as a glasslike foam. [α]_25_^D^ = +35 (*c* = 1.3, CHCl_3_). ^1^H NMR (400 MHz, CD_3_OD) δ = 7.40–7.22 m (5HH^ar^), 5.08 s (2H, CH_2_O), 4.55 t (1H, *J* = 6.8 and 5.4 Hz, H-2), 3.26 t (2H, *J* = 6.5 Hz, C^6^H_2_N), 2.98 m (2H, *J* = 9.0 and 4.4 Hz, H-3), 2.64 m (2H, C^5^H_2_), 1.39 s (9H, OCOBu^t^) ppm. ^13^C­{^1^H} NMR (101 MHz, CD_3_OD) δ = 208.0 (CO), 174.8 (COO), 158.4 (NHCOO), 138.1 (C_q_), 129.4 (CH), 129.0 (CH), 128.8 (CH), 80.1 (C_q_O), 67.7 (CH_2_O), 67.7 (CH_2_O), 51.0 (C^2^HNH), 45.0 (C^3^H_2_), 43.6 (C^6^H_2_), 36.3 (C^5^H_2_), 28.7 (3 × Me) ppm. HRMS (ESI-TOF) *m*/*z* [M + Na]^+^ calcd for C_19_H_26_N_2_O_7_Na [M + Na]^+^ 417.1632; found 417.1626.

### Compound **7**

(*S*)-BocNH­(CH_2_)_2_COCH_2_CH­(NHCO_2_CH_2_C_6_H_5_)­CO_2_Bu^*t*^. Carboxylic acid **6** (0.95 g, 2.4 mmol) was dissolved in DCM (14 mL) and cooled to 0 °C. Cl_3_C­(=NH)­OBu^t^ (1.05 g, 4.83 mmol) was dissolved in anhydrous DCM (5 mL), and this solution was added to the cold solution of carboxylic acid **6**. The reaction mixture was stirred at 0 °C, and then BF_3_*Et_2_O (25 mL) was added. The precipitate formed gradually, which dissolved, however, next day upon the addition of DCM (20 mL) and on warming up to room temperature. The reaction was quenched by adding 5% aq. NaHCO_3_ (3 mL) to neutralize BF_3_*Et_2_O. The reaction mixture was diluted with EtOAc (50 mL), and the aq. layer was separated and extracted with EtOAc (20 mL). The combined organic layers were dried and evaporated to afford 2.0 g of the residue. Compound **7** was isolated by flash chromatography on SiO_2_ (25 g); elution with an EtOAc gradient (5–60%) in a cyclohexane–DCM mixture (2:1) afforded 0.864 g (77%) of compound **7** as a colorless gum. [α]_25_^D^ = +13 (*c* = 1.1, CHCl_3_). ^1^H NMR (400 MHz, CD_3_OD) δ = 7.43–7.22 m (5HH^ar^), 5.08 s (2H, CH_2_O), 4.43 t (1H, *J* = 6.0 Hz, H-2), 3.26 t (2H, *J* = 6.5 Hz, C^6^H_2_N), 3.02–2.86 m (2H, H-3), 2.64 tm (2H, *J* = 6.5 and 3.2 Hz, C^5^H_2_), 1.43 s (9H, CO_2_Bu^t^), 1.40 s (9H, OCOBu^t^) ppm. ^13^C­{^1^H} NMR (101 MHz, CD_3_OD): δ = 208.0 (CO), 172.2 (COO), 158.4 (NHCOO), 138.1 (C_q_), 129.5 (CH), 129.0 (CH), 128.9 (CH), 83.0 (C_q_O), 80.1 (C_q_O), 67.7 (CH_2_O), 51.9 (C^2^HNH), 45.0 (C^3^H_2_), 43.6 (C^5^H_2_), 36.3 (C^6^H_2_), 28.7 (3 × Me), 28.2 (3 × Me) ppm. HRMS (ESI-TOF) *m*/*z* [M + Na]^+^ calcd for C_23_H_34_N_2_O_7_Na 473.2258; found 473.2254.

### Compound **8**

Epimers 1 and 2. Compound **7** (0.79 g, 1.75 mmol) was dissolved in MeOH (15 mL), and the solution was cooled in an ice bath. NaBH_4_ (146 mg, 3.86 mmol) was added in one portion to the solution with stirring, and the mixture was stirred at room temperature for several hours. The products **8**–epimer 1 and **8**–epimer 2 have somewhat (marginally) lower *R*_f_ values than the starting material (TLC on regular SiO_2_ with hexane–EtOAc, 2:1). When the reaction was completed, AcOH (1 mL) was added to the reaction mixture, and it was stirred for 30 min at room temperature. Then, the reaction mixture was transferred into the separation funnel containing EtOAc (100 mL) and 1 M aq. KHSO_4_ solution (20 mL). The organic layer was separated (after shaking), and the aq. layer was extracted with EtOAc (50 mL). Combined organic layers were washed with sat. NaHCO_3_ (50 mL), dried, and evaporated. The residue (0.90 g) was subjected to flash chromatography on SiO_2_ (25 g). Elution with an EtOAc gradient (10–90%) in cyclohexane afforded fractions containing pure epimers 1 and 2 of compound **8**, as well as an intermediate fraction. The latter was evaporated and separated on 10 g of SiO_2_ (cartridge). Fractions containing pure epimers were combined and evaporated to afford compound **8**–epimer 1 (higher *R*_f_, 406 mg, 51%) and compound **8**–epimer 2 (lower *R*_f_, 308 mg, 39%) obtained as glasslike solids (upon drying in vacuo). Compound **8**–epimer 1: (2*S*,4*R*)-BocNH­(CH_2_)_2_CH­(OH)­CH_2_CH­(NHCO_2_CH_2_C_6_H_5_)-CO_2_Bu^*t*^. ^1^H NMR (400 MHz, CD_3_OD) δ = 7.44–7.22 m (5HH^ar^), 5.09 s (2H, CH_2_O), 4.29 dd (1H, *J* = 10.3 and 3.8 Hz, H-2), 3.68 m (1H, H-4), 3.13 m (2H, C^6^H_2_N), 1.77 m (2H, C^5^H_2_/C^3^H_2_), 1.57 m (2H, C^3^H_2_/C^5^H_2_), 1.45 s (9H, CO_2_Bu^t^), 1.39 s (9H, OCOBu^t^) ppm. ^13^C­{^1^H} NMR (101 MHz, CD_3_OD) δ = 173.8 (COO), 158.4 (NHCOO), 138.2 (C_q_), 129.4 (CH), 129.0 (CH), 128.9 (CH), 82.6 (C_q_O), 80.0 (C_q_O), 67.6 (CH_2_O), 66.4 (C^4^H), 53.6 (C^2^HNH), 39.6 (C^3^H_2_), 39.0 (C^5^H_2_), 38.1 (NC^6^H_2_), 28.8 (3 × Me), 28.2 (3 × Me) ppm. HRMS (ESI-TOF) *m*/*z*: [M + Na]^+^ calcd for C_23_H_36_N_2_O_7_Na 475.2415; found 475.2410.

Compound **8**–epimer 2: (2*S*,4*S*)-BocNH­(CH_2_)_2_CH­(OH)­CH_2_CH­(NHCO_2_CH_2_C_6_H_5_)­CO_2_Bu^*t*^. ^1^H NMR (400 MHz, CD_3_OD) δ = 7.40–7.21 m (5HH^ar^), 5.10/5.08 (2H, AB-system, CH^A^H^B^O, *J* = 13 Hz), 4.17 t (1H, *J* = 6.9 Hz, H-2), 3.74 tt (1H, *J* = 8.5 and 4.2 Hz, H-4), 3.14 t (2H, *J* = 6.8 Hz, H-6), 1.88 ddd (1H, *J* = 14.0, 6.7, and 4.8 Hz, C^5^H^b^/C^3^H^b^), 1.77 dt (1H, *J* = 14.4 and 7.6 Hz, C^5^H^a^/C^3^H^a^), 1.63 m (1H, C^3^H^a^/C^5^H^a^), 1.51 m (1H, C^3^H^b^/C^5^H^b^), 1.44 s (9H, CO_2_Bu^t^), 1.41 s (9H, OCOBu^t^) ppm. ^13^C­{^1^H} NMR (101 MHz, CD_3_OD) δ = 173.3 (COO), 158.6/158.4 (NHCOO), 138.2 (C_q_), 129.5 (CH), 129.0 (CH), 128.8 (CH), 82.7 (C_q_O), 80.0 (C_q_O), 67.6 (CH_2_O), 67.1 (C^4^H), 53.8 (C^2^HNH), 40.3 (C^5^H_2_/C^3^H_2_), 38.18 (NC^6^H_2_), 38.11 (C^3^H_2_/C^5^H_2_), 28.8 (3 × Me), 28.2 (3 × Me) ppm. HRMS (ESI-TOF) *m*/*z* [M + Na]^+^ calcd for C_23_H_36_N_2_O_7_Na [M + Na]^+^ 475.2415; found 475.2408.

### Compound **9**

Epimer 1: (2*S*,4*S*)-BocNH­(CH_2_)_2_CHFCH_2_CH (NHCO_2_CH_2_C_6_H_5_)­CO_2_Bu^*t*^. Compound **8**–epimer 1 (337 mg, 0.74 mmol) was dissolved in dry toluene (2.0 mL). PyFluor reagent (2-(fluorosulfonyl)­pyridine, 132 mg, 0.82 mmol) was added, and the mixture was stirred for 15 min. Then, the DBU base (0.221 mL, 225 mg, 1.48 mmol) was added. The precipitate started to deposit in several minutes, and the reaction mixture gradually turned yellow. The reaction mixture was stirred at room temperature for 48 h. TLC analysis (hexane/EtOAc, 2/1) indicated the complete conversion into the new substance with a higher *R*_f_ value. The reaction mixture was carefully diluted with DCM (ca. 1:1), until the precipitate was dissolved, and then it was directly applied onto the cartridge containing 25 g of SiO_2_ equilibrated with 5% EtOAc in a cyclohexane–DCM mixture (3:1). Elution with 5–70% EtOAc in the cyclohexane–DCM mixture (3:1) afforded 261 mg (71%) of compound **9**–epimer 1 as a glassy oil. [α]_25_^D^ = +13 (*c* = 1.7, CHCl_3_). ^1^H NMR (400 MHz, CD_3_CN) δ = 7.47–7.30 m (5HH^ar^), 5.96 br. d (1H, *J* = 8.6 Hz, NH), 5.40 br. s (1H, NH), 5.11/5.08 (2H, AB-system, CH^A^H^B^O, *J* = 12 Hz), 4.66 dm (1H, ^2^ *J*_HF_ = 50 Hz, CH-F), 4.20 ddd (1H, *J* = 11.6, 8.5, and 3.5 Hz, H-2), 3.14 m (2H, *J* = 6.7 and 3.6 Hz, C^6^H_2_N), 2.19–2.01 m (2H, C^5^H_2_/C^3^H_2_), 1.94–1.59 m (2H, C^3^H_2_/C^5^H_2_), 1.45 s (9H, CO_2_Bu^t^), 1.41 s (9H, OCOBu^t^) ppm. ^13^C–^1^H correlation NMR (101/400 MHz, gHSQCAD, CD_3_CN) δ (^13^C) = 129 (3 × CH), 89.9 d (^1^ *J*_CF_ = 171 Hz, C^4^H–F), 67.1 (CH_2_O), 52.7 (C^2^HNH), 37.5 (C^5^H_2_/C^3^H_2_), 37.2 (NC^6^H_2_), 36.4 d (C^3^H_2_/C^5^H_2_), 28.4 (3 × Me), 28.0 (3 × Me) ppm. ^19^F NMR (376 MHz, CD_3_CN) δ = −182.8 dt (*J* = 48 and 24 Hz). HRMS (ESI-TOF) *m*/*z*: [M + Na]^+^ calcd for C_23_H_35_FN_2_O_6_Na [M + Na]^+^ 477.2371; found 477.2366.

### Compound **9**

Epimer 2: (2*S*,4*R*)-BocNH­(CH_2_)_2_CHFCH_2_CH (NHCO_2_CH_2_C_6_H_5_)­CO_2_Bu^*t*^. Under reaction conditions and with the isolation procedure given above, compound **8**–epimer 2 (282 mg, 0.62 mmol), PyFluor (0.69 mmol, 111 mg), and DBU base (188 mg, 1.24 mmol) afforded 133 mg (47%) of compound **9**–epimer 2. Therefore, another strong base was applied.^24^ Compound **8**–epimer 2 (312 mg, 0.69 mmol), PyFluor (122 mg, 0.76 mmol), and 7-methyl-1,5,7-triazobicyclo[4.4.0]­undecene-5 (MTBD, 226 mg, 0.213 mL, 1.48 mmol) were combined in dry toluene (2.0 mL). The reaction mixture gradually turned orange, and the orange phase separated. The reaction mixture was stirred at room temperature for 48 h. Isolation (performed as described above) afforded 169 mg (54%) of compound **9**–epimer 2 as a viscous oil. [α]_25_^D^ = +3.1 (*c* = 1.8, CHCl_3_). ^1^H NMR (400 MHz, CD_3_CN) δ = 7.44–7.30 m (5HH^ar^), 6.03 br. d (1H, *J* = 8.0 Hz, NH), 5.34 br. s (1H, NH), 5.11/5.08 (2H, AB-system, CH^A^H^B^O, *J* = 12 Hz), 4.72 dm (1H, ^2^ *J*_HF_ = 49 Hz, CH-F), 4.18 q (1H, *J* = 6.8 Hz, H-2), 3.14 m (2H, *J* = 6.7 and 4.8 Hz, C^6^H_2_N), 2.05 dm (2H, ^3^ *J*_HF_ = 24 Hz, C^5^H_2_/C^3^H_2_), 1.76 dm (2H, ^3^ *J*_HF_ = 24 Hz, C^3^H_2_/C^5^H_2_), 1.44 s (9H, CO_2_Bu^t^), 1.41 s (9H, OCOBu^t^) ppm. ^13^C­{^1^H} NMR (101 MHz, CD_3_CN) δ = 171.7 (COO), 156.9 (NHCOO), 138.1 (C_q_), 129.4 (CH), 128.9 (CH), 128.7 (CH), 90.5 d (^1^ *J*_CF_ = 166 Hz, C^4^H–F), 82.5 (C_q_O), 79.2 (C_q_O), 67.1 (CH_2_O), 52.9 (C^2^HNH), 37.9 d (^2^ *J*_CF_ = 21 Hz, C^5^H_2_/C^3^H_2_), 37.2 (NC^6^H_2_), 36.0 d (^2^ *J*_CF_ = 21 Hz, C^3^H_2_/C^5^H_2_), 28.6 (3 × Me), 28.1 (3 × Me) ppm. ^19^F NMR (376 MHz, CD_3_CN): δ = −184.1 ppm (m). HRMS (ESI-TOF) *m*/*z*: [M + Na]^+^ calcd for C_23_H_35_FN_2_O_6_Na 477.2371; found 477.2366.

### Compound **10**

Epimer 1: (2*S*,4*S*)-H_2_N­(CH_2_)_2_CHFCH_2_CH (NHCO_2_CH_2_C_6_H_5_)­CO_2_H. Compound **9**–epimer 1 (258 mg, 0.568 mmol) was dissolved in DCM (2.5 mL); then, TFA (0.75 mL) and Et_3_SiH (0.37 mL) were added, and the reaction mixture was stirred at room temperature for 1.5 h. First, *tert*-butyl carbamate was cleaved off, while *tert*-butyl ester was more stable. The course of the reaction was controlled by TLC on regular SiO_2_ (the spot of the starting material disappeared) and by TLC on RP-C18; eluent: aq. 50 mM Et_3_N-HCO_2_H buffer (pH ∼ 6) with 25% (v/v) CD_3_CN. On the RP-C_18_ plate, the spot of the starting material did not move and stayed on the start, the spot of the final product, compound **10**–epimer 1, had the highest *R*_f_, while the spot of the intermediate (*tert*-butyl ester) had a lower *R*_f_ than that of compound **10**–epimer 1. To complete the reaction, it was necessary to add more TFA (1.5 mL), 0.5 mL of Et_3_SiH, and 2.0 mL of DCM to the reaction mixture. After stirring for 2 h at room temperature, the reaction was completed, and TLC on the RP-C18 plate displayed one spot. Volatile materials were removed in vacuo, and the residue [taken up in minimal amount of CH_3_CN with 10% v/v of 1 M aq. Et_3_N-HCO_2_H buffer (pH ∼ 6)] was applied onto the column with RP-C18 SiO_2_ (50 g) equilibrated with 50 mM Et_3_N-HCO_2_H buffer (pH ∼ 6) with 10% (v/v) CD_3_CN. Elution with 10–40% v/v CD_3_CN in aq. buffer (see above) followed by collecting the fractions containing compound **10**–epimer 1 (TLC control with ninhydrin detection) and lyophilization afforded the product as a colorless powder. It was dissolved in water (20 mL) and lyophilized (2 times) to remove Et_3_N and HCO_2_H as full as possible. According to ^1^H and ^19^F NMR spectra, the residue had the following composition: H_2_N­(CH_2_)_2_CHFCH_2_CH­(NHCOOCH_2_C_6_H_5_)­COOH × 0.2 CF_3_CO_2_H × 0.2 (C_2_H_5_)_3_N (*M* = 341); yield 179 mg (0.524 mmol, 92%). [α]_25_^D^ = +36 (*c* = 0.27, 0.1 M aq. HCl). ^1^H NMR (400 MHz, CD_3_CN) δ = 7.43–7.23 m (5HH^ar^), 5.07/5.03 (2H, AB-system, CH^A^H^B^O, *J* = 12 Hz), 4.79 dm (1H, ^2^ *J*_HF_ = 51 Hz, CH-F), 4.00 t (1H, *J* = 6.5 Hz, H-2), 3.05 m (2H, C^6^H_2_N), 2.10–1.87 m (4H, C^5^H_2_ + C^3^H_2_) ppm. ^13^C­{^1^H} NMR (101 MHz, D_2_O) δ = 165.2 br. (COOH), 160.2 (OCON), 138.9 (C_q_), 131.3 (2 × CH), 130.9 (CH), 130.2 (CH), 93.4 d (^1^ *J*_CF_ = 165 Hz, C^4^H–F), 69.6 (CH_2_O), 54.4 (C^2^HNH), 38.8 (NC^6^H_2_), 38.7 d (^2^ *J*_CF_ = 15 Hz, C^5^H_2_/C^3^H_2_), 34.3 d (^2^ *J*_CF_ = 20 Hz, C^3^H_2_/C^5^H_2_) ppm. ^19^F NMR (376 MHz, D_2_O): δ = −182.1 m. HRMS (ESI-TOF) *m*/*z*: [M + Na]^+^ calcd for C_14_H_19_FN_2_O_4_Na 321.1221; found 321.1220.

### Compound **10**

Epimer 2: (2*S*,4*R*)-H_2_N­(CH_2_)_2_CHFCH_2_CH­(NHCO_2_CH_2_C_6_H_5_)­CO_2_H. Compound **9**–epimer 2 (133 mg, 0.293 mmol) was dissolved in DCM (1.5 mL), and then TFA (0.38 mL) and Et_3_SiH (0.18 mL) were added, and the reaction mixture was stirred at room temperature for 3.5 h. TLC on the RP-C18 plate displayed one single spot of the product. For details of TLC analysis, see the preparation of compound **10**–epimer 1 above. Volatile materials were removed in vacuo, and the residue [taken up in minimal amount of CH_3_CN with 10% v/v of 1 M aq. Et_3_N-HCO_2_H buffer (pH ∼ 6)] was applied onto the column with RP-C18 SiO_2_ (50 g) equilibrated with 50 mM Et_3_N-HCO_2_H buffer (pH ∼ 6) with 10% (v/v) CD_3_CN. Elution with 10–40% v/v CD_3_CN in aq. buffer (see above) followed by collecting the fractions containing compound **10**–epimer 2 (TLC control with ninhydrin detection) and lyophilization afforded the product (colorless powder). It was dissolved in water (20 mL) and lyophilized (2 times) to remove Et_3_N and HCO_2_H as full as possible. According to ^1^H and ^19^F NMR spectra, the residue had the following composition: H_2_N­(CH_2_)_2_CHFCH_2_CH­(NHCOOCH_2_C_6_H_5_)­COOH × 1.0 CF_3_CO_2_H × 0.6 (C_2_H_5_)_3_N (*M* = 472); yield 115 mg (0.243 mmol, 92%). [α]_25_^D^ = +3.3 (*c* = 0.20, 0.1 M aq. HCl). ^1^H NMR (400 MHz, CD_3_CN): δ = 7.43–7.25 m (5H, H^ar^), 5.08/5.03 (2H, AB-system, CH^A^H^B^O, *J* = 12 Hz), 4.71 dm (1H, ^2^ *J*_HF_ = 48 Hz, CH-F), 4.10 m (1H, H-2), 3.04 t (22H, *J* = 6.9 Hz, C^6^H_2_N), 2.15 m (1H, *J* = 14.8, 10.9, and 3.8 Hz, C^5^H/C^3^H), 2.03–1.77 m (3H, C^5^H + C^3^H) ppm. ^13^C­{^1^H} NMR (101 MHz, D_2_O) δ = 165.6 br. (COOH), 160.5 (OCON), 138.9 (C_q_), 131.3 (2 × CH), 130.9 (CH), 130.2 (CH), 96.6 d (^1^ *J*_CF_ = 166 Hz, C^4^H–F), 69.7 (CH_2_O), 53.9 (C^2^HNH), 38.9 d (*J* = 4 Hz, NC^6^H_2_), 38.7 d (^2^ *J*_CF_ = 21 Hz, C^5^H_2_/C^3^H_2_), 34.4 d (^2^ *J*_CF_ = 20 Hz, C^3^H_2_/C^5^H_2_) ppm. ^19^F NMR (376 MHz, D_2_O): δ = −183.1 m. HRMS (ESI-TOF) *m*/*z*: [M + H]^+^ calcd for C_14_H_20_FN_2_O_4_ 299.1402; found 299.1400.

### Compound **11**

Epimer 1: (2*S*,4*S*)-H_2_N­(CH_2_)_2_CHFCH_2_CH­(NH_2_)­CO_2_H. Pd/C (120 mg, MERCK, oxidized form, 10% Pd) was suspended in 15 mL of aq. THF (10% v/v THF) in a Schlenk flask, flushed with argon and then with H_2_, and vigorously stirred under hydrogen for 15 min. Compound **10**–epimer 1 (116 mg, 0.34 mmol; see above) was dissolved in water, and this solution was injected into the flask with the stirred suspension of prereduced Pd/C. When the reaction was completed (for conditions of TLC analysis on RP-C18, see syntheses above), the catalyst was filtered off through Celite, the filter cake was washed with water, and the filtrate was lyophilized. The residue was dissolved in water (15 mL) and lyophilized once more to afford 70 mg of a voluminous colorless cottonlike “solid” material. According to ^1^H and ^19^F NMR spectra, the residue after the final lyophilization had the following composition: H_2_N­(CH_2_)_2_CHFCH_2_CH­(NH_2_)­COOH × 0.33 CF_3_CO_2_H (*M* = 202); quantitative yield. A sample was applied onto a strong cation exchanger (IR-120, NH_4_^+^ form); the column was washed with water and then with a gradient of NH_3_ in water (0.5–5%). Fractions containing amino acid (TLC on regular SiO_2_ with BuOH/AcOH/H_2_O = 3/1/1 mixture, developed with ninhydrin) were pooled, lyophilized, dissolved in pure water, and lyophilized two more times. The residue was dissolved in water, carefully neutralized with 0.1 M aq. HCl to pH = 4, and lyophilized to obtain monohydrochloride. Mp 219–220 °C (decomp.). [α]_25_^D^ = +16 (*c* = 0.76, H_2_O). ^1^H NMR (400 MHz, D_2_O) δ = 5.00 dm (1H, ^2^ *J*_HF_ = 50 Hz, CH-F), 3.82 t (1H, *J* = 6.5 Hz, H-2), 3.19 t (2H, *J* = 7.2 Hz, C^6^H_2_N), 2.35–1.96 m (4H, C^5^H_2_ + C^3^H_2_) ppm. ^13^C­{^1^H} NMR (101 MHz, D_2_O) δ = 175.3 (COOH), 91.6 (^1^ *J*_CF_ = 165 Hz, C^4^H–F), 52.8 (C^2^HNH), 36.4 (*J* = 20 Hz, C^5^H_2_/C^3^H_2_), 36.2 (^3^ *J*_CF_ = 4 Hz, NC^6^H_2_), 32.3 (^2^ *J*_CF_ = 20 Hz, C^3^H_2_/C^5^H_2_) ppm. ^19^F NMR (376 MHz, D_2_O): δ = −183.5 m. HRMS (ESI-TOF) *m*/*z*: [M + H]^+^ calcd for C_6_H_14_FN_2_O_2_ [M + H]^+^ 165.1034; found 165.1033.

### Compound **11**

Epimer 2: (2*S*,4*R*)-H_2_N­(CH_2_)_2_CHFCH_2_CH­(NH_2_)­CO_2_H. It was obtained and isolated according to the procedure given above for epimer 1. The starting compound was compound **10**–epimer 2 (59 mg, 0.125 mmol); catalyst: 100 mg of prereduced Pd/C (10% Pd, oxidized form). According to the ^1^H and ^19^F NMR spectra, the product (residue after final lyophilization) had the following composition: H_2_N­(CH_2_)_2_CHFCH_2_CH­(NH_2_)­COOH × 0.90 CF_3_CO_2_H × 0.11 Et_3_N (M = 278); yield 33 mg (95%) of a colorless hygroscopic foam. [α]_25_^D^ = −40 (*c* = 0.14, H_2_O). ^1^H NMR (400 MHz, D_2_O) δ = 4.71 dm (1H, ^2^ *J*_HF_ = 52 Hz, CH-F), 3.96 dd (1H, *J* = 7.1 and 3.8 Hz, H-2), 3.20 t (2H, *J* = 7.1 Hz, C^6^H_2_NH), 2.40–2.15 m (2H, C^5^H/C^3^H), 2.14–1.94 m (2H, C^5^H + C^3^H) ppm. ^13^C­{^1^H} NMR (101 MHz, CD_3_CN) δ = 173.6 (COOH), 90.7 d (^1^ *J*_CF_ = 166 Hz, C^4^H–F), 52.0 d (^3^ *J*_CF_ = 1.8 Hz, C^2^HNH), 36.2 d (^3^ *J* = 4 Hz, NC^6^H_2_), 35.1 d (^2^ *J*_CF_ = 20 Hz, C^5^H_2_/C^3^H_2_), 32.0 d (^2^ *J*_CF_ = 20 Hz, C^3^H_2_/C^5^H_2_) ppm. ^19^F NMR (376 MHz, CD_3_CN): δ = −183.6 m_c_. HRMS (ESI-TOF) *m*/*z*: [M + H]^+^ calcd for C_6_H_14_FN_2_O_2_ 165.1034; found 165.1033.

### Compound **12**

Epimer 1: (2*S*,4*S*)-BocNH­(CH_2_)_2_CHFCH_2_CH (NH_2_)­CO_2_Bu^*t*^. Compound **9**–epimer 1 (181 mg, 0.399 mmol) was dissolved in THF (15 mL) and hydrogenated with stirring over 94 mg of prereduced Pd/C (10% Pd, oxidized form). In several hours, the reaction was completed; TLC, hexane/EtOAc = 7/3; the spot of the product has a lower *R*_f_. The catalyst was removed by filtration of centrifugation and washed with THF, and the filtrate was evaporated to afford 130 mg (100%) of compound **12**–epimer 1 as a glassy foam, which was used in the next step immediately. ^1^H NMR (400 MHz, CD_3_CN) δ = 5.46 br. s (1H, NHCO), 4.76 dm (1H, ^2^ *J*_HF_ = 48 Hz, CH-F), 3.39 t (1H, *J* = 6.5 Hz, H-2), 3.21–3.09 m (2H, C^6^H_2_N), 1.92–1.85 m (4H, NH_2_ + C^5^H_2_/C^3^H_2_), 1.82–1.72 m (2H, C^3^H_2_/C^5^H_2_), 1.46 s (9H, Me_3_C), 1.41 s (9H, Me_3_C) ppm. ^13^C–^1^H correlation NMR (101/400 MHz, gHSQCAD, CD_3_CN) δ = 90.1 d (^1^ *J*_CF_ = 184 Hz, C^4^H–F), 51.9 (C^2^HNH), 39.2 d (^2^ *J*_CF_ = 18 Hz, C^5^H_2_/C^3^H_2_), 36.2 d (^3^ *J* = 4 Hz, NC^6^H_2_), 35.1 d (^2^ *J*_CF_ = 18 Hz, C^3^H_2_/C^5^H_2_), 27.6 (3 × Me), 27.2 (3 × Me) ppm. ^19^F NMR (376 MHz, D_2_O): δ = −183.1 m_c_. (320.4)

### Compound **12**

Epimer 2: (2*S*,4*R*)-BocNH­(CH_2_)_2_CHFCH_2_CH­(NH_2_)­CO_2_Bu^*t*^. Compound **9**–epimer 2 (144 mg, 0.317 mmol) was dissolved in THF (10 mL) and hydrogenated with stirring over 88 mg of prereduced Pd/C (10% Pd, oxidized form). In several hours, the reaction was completed; TLC, hexane/EtOAc = 7/3; the spot of the product has a lower *R*_f_. The catalyst was removed by filtration of centrifugation and washed with THF, and the filtrate was evaporated to afford 101 mg (100%) of compound **12**–epimer 2 as a glasslike foam, which was used in the next step immediately. ^1^H NMR (400 MHz, CD_3_CN) δ = 5.39 br. s (1H, NHCO), 4.84 dm (1H, ^2^ *J*_HF_ = 48 Hz, CH-F), 3.36 dd (1H, *J* = 10.3 and 3.6 Hz, H-2), 3.16 m_c_ (2H, C^6^H_2_N), 2.01 m (1H, C^5^H/C^3^H), 1.94 br. s (2H, NH_2_), 1.83–1.70 m (2H, C^5^H_2_/C^3^H_2_), 1.64–1.48 m (1H, C^3^H + C^5^H), 1.46 s (9H, Me_3_C), 1.42 s (9H, Me_3_C) ppm. ^13^C–^1^H correlation NMR (101/400 MHz, gHSQCAD, CD_3_CN) δ = 89.6 d (^1^ *J*_CF_ = 165 Hz, C^4^H–F), 51.7 (C^2^HNH), 39.9 d (^2^ *J*_CF_ = 23 Hz, C^5^H_2_/C^3^H_2_), 36.4 (NC^6^H_2_), 35.4 (C^3^H_2_/C^5^H_2_), 27.6 (3 × Me), 27.2 (3 × Me) ppm. ^19^F NMR (376 MHz, CD_3_CN): δ = −184.7 m_c_.

### Compound **13**

Epimer 1: (2*S*,4*S*)-BocNH­(CH_2_)_2_CHFCH_2_CH­(NHFmoc)­CO_2_Bu^*t*^. Compound **12**–epimer 1 (130 mg, 0.406 mmol) was dissolved in THF (1.5 mL) and CH_3_CN (1.5 mmol), and Fmoc-OSu (200 mg, 0.593 mmol) was added followed by 1.1 mL of 5% aq. solution of NaHCO_3_. The reaction mixture was vigorously stirred overnight at room temperature. The precipitate appeared. The reaction mixture was acidified to pH 2–3 with 1 M aq. KHSO_4_and diluted with EtOAc (50 mL). The aq. layer was separated, and the organic layer was dried and evaporated. The residue was subjected to flash chromatography on SiO_2_ (10 g cartridge). 9-Methylene-9*H*-fluorene has the highest *R*_f_, Fmoc-OSu has the lowest *R*_f_, and compound **13**–epimer 1 has an intermediate *R*_f_. Elution with an EtOAc gradient in cyclohexane (5–90%) afforded 169 mg (94%) of compound **13**–epimer 1 as a colorless solid. Recrystallization from acetonitrile of a sample (34 mg) gave 25 mg (74%) of prisms (plates) with mp 171–173 °C. X-ray analysis: C_30_H_39_FN_2_O_6_; see the Supporting Information. [α]_25_^D^ = +7.7 (*c* = 0.86, CHCl_3_). ^1^H NMR (400 MHz, CDCl_3_) δ = 7.78 d (2H, *J* = 7.5 Hz, H^ar^), 7.61 d (2H, *J* = 7.5 Hz, H^ar^), 7.41 t (2H, *J* = 7.5 Hz, H^ar^), 7.38–7.29 m (2H, H^ar^), 5.64 d (1H, *J* = 6.8 Hz, NH), 4.74 dm (1H, ^1^ *J*_HF_ = 52 Hz, CH-F), 4.70 m (1H, NH), 4.41 d (2H, *J* = 7.1 Hz, CHCH_2_O), 4.23 t (1H, *J* = 6.9 Hz, CHCH_2_O), 3.27 m (2H, C^6^H_2_N), 2.26–2.05 m (2H, C^5^H/C^3^H), 1.92–1.75 m (2H, C^3^H_2_/C^5^H_2_), 1.48 s (CMe_3_), 1.44 s (CMe_3_) ppm. ^13^C­{^1^H} NMR (101 MHz, CDCl_3_) δ = 170.6 (COO), 156.0/155.8 (2 × NCOO), 143.9/143.8 (2 × *C*_q_), 141.3 (C_q_), 127.8 (CH), 127.1 (CH), 125.1 (CH), 120.0 (CH), 89.4 d (^1^ *J*_CF_ = 167 Hz, C^4^H–F), 82.7 (C_q_-O), 67.0 (CH_2_O), 51.7 (C^2^HN), 47.2 (CH), 37.6 d (^2^ *J*_CF_ = 21 Hz, C^5^H_2_/C^3^H_2_), 37.0 (NC^6^H_2_), 35.2 d (^2^ *J*_CF_ = 20 Hz, C^5^H_2_/C^3^H_2_), 28.4 (CMe_3_), 27.9 (CMe_3_) ppm. ^19^F NMR (376 MHz, D_2_O): δ = −185.0 ddq (*J* = 50, 32, and 16 Hz).

### Compound **13**

Epimer 2: (2*S*,4*R*)-BocNH­(CH_2_)_2_CHFCH_2_CH­(NHFmoc)­CO_2_Bu^*t*^. Compound **12**–epimer 2 (101 mg, 0.316 mmol) was dissolved in THF (1.5 mL) and CH_3_CN (1.5 mmol), and Fmoc-OSu (150 mg, 0.445 mmol) was added followed by 1.1 mL of 5% aq. solution of NaHCO_3_. The reaction mixture was vigorously stirred overnight at room temperature. The reaction mixture was acidified to pH 2–3 with 1 M aq. KHSO_4_ and diluted with EtOAc (50 mL). The aq. layer was separated, and the organic layer was dried and evaporated. The residue was subjected to flash chromatography on SiO_2_ (10 g cartridge). 9-Methylene-9*H*-fluorene has the highest *R*_f_, Fmoc-OSu has the lowest *R*_f_, and compound **13**–epimer 2 has an intermediate *R*_f_. Elution with an EtOAc gradient in cyclohexane (5–90%) afforded 133 mg (95%) of compound **13**–epimer 2 (a glasslike mass), which was used in the next step as such.

### Compound **14**

Epimer 1: (2*S*,4*S*)-NH_2_(CH_2_)_2_CHFCH_2_CH­(NHFmoc)­CO_2_H. Compound **13**–epimer 1 (175 mg, 0.396 mmol) in 2.0 mL of DCM was treated with 0.5 mL of *i*Pr_3_SiH and 1.0 mL of TFA. The reaction mixture was stirred at room temperature for 1.5 h, and then 0.5 mL of *i*Pr_3_SiH and 0.8 mL of TFA were added. The reaction mixture was stirred at room temperature overnight. All volatile materials were removed in vacuo; the residue was taken up in aq. MeCN (1:1; ∼3 mL) and applied onto a prepacked column with RP-C_18_ (25–30 g SiO_2_) equilibrated with a CH_3_CN/H_2_O (1:4) mixture with 0.1% TFA. The product was eluted with a gradient (25–33% v/v) of acetonitrile in water (+0.1% v/v TFA in both components). Pure fractions with the product were pooled and lyophilized to afford compound **14**–epimer 1 (TFA salt) as a colorless powder (156 mg (79%)). [α]_25_^D^ = −99 (*c* = 0.33, MeOH). ^1^H NMR (400 MHz, CD_3_CN) δ = 7.85 d (2H, *J* = 7.5 Hz, H^ar^), 7.69 dd (2H, *J* = 7.7 and 4.0 Hz, H^ar^), 7.49–7.41 m (2H, H^ar^), 7.36 td (2H, *J* = 7.4 and 1.1 Hz, H^ar^), 7.11 s (1H, NHCO), 6.24 br. d (1H, *J* = 7.6 Hz, NHCO), 4.86 dm (1H, ^2^ *J*_HF_ = 49 Hz, CH-F), 4.41–4.34 m (2H, CHCH_2_O), 4.29 m (1H, CHCH_2_O), 4.26 m (1H, CHN), 3.12 m (2H, C^6^H_2_N), 2.21–2.09 m (2H, C^5^H_2_/C^3^H_2_), 2.08–1.98 m (2H, C^3^H_2_/C^5^H_2_) ppm. ^13^C­{^1^H} NMR (101 MHz, CD_3_CN) δ = 173.6 (COO), 157.1 (NCOO), 145.1 (C_q_), 142.2 (C_q_), 128.8 (CH), 128.2 (CH), 126.3 (CH), 121.1 (CH), 90.9 d (^1^ *J*_CF_ = 167 Hz, C^4^H–F), 67.6 (CH_2_O), 52.0 (C^2^HN), 48.1 (CH), 37.7­(NC^6^H_2_), 37.6 d (^2^ *J*_CF_ = 18 Hz, C^5^H_2_/C^3^H_2_), 32.9 d (^2^ *J*_CF_ = 20 Hz, C^3^H_2_/C^5^H_2_) ppm. ^19^F NMR (376 MHz, D_2_O) δ = −183.5 m_c_ (*J* = 49 and 25 Hz). HRMS (ESI-TOF) *m*/*z*: [M + H]^+^ calcd for C_21_H_24_FN_2_O_4_ 387.1715; found 387.1713.

### Compound **14**

Epimer 2: (2*S*,4*R*)-NH_2_(CH_2_)_2_CHFCH_2_CH­(NHFmoc)­CO_2_H. Compound **13**–epimer 2 (162 mg, 0.367 mmol) in 2.0 mL DCM was treated with 0.5 mL of *i*Pr_3_SiH and 1.0 mL of TFA. The reaction mixture was stirred at room temperature for 1.5 h, and then 0.5 mL of *i*Pr_3_SiH and 0.8 mL of TFA were added. The reaction mixture was stirred at room temperature overnight. All volatile materials were removed in vacuo, and the residue was taken up in aq. MeCN (1:1; ∼3 mL) and applied onto a prepacked column with RP-C_18_ (25–30 g SiO_2_) equilibrated with a CH_3_CN/H_2_O (1:4) mixture with 0.1% TFA. The product was eluted with a gradient (25–33% v/v) of acetonitrile in water (+0.1% v/v TFA in both components). Pure fractions with the product were pooled and lyophilized to afford compound **14**–epimer 1 (TFA salt) as colorless flakes (142 mg (77%)). [α]_25_^D^ = −8.0 (*c* = 0.31, 50% v/v aq. MeOH). ^1^H NMR (400 MHz, CD_3_CN) δ = 7.82 d (2H, *J* = 7.5 Hz, H^ar^), 7.68–7.63 m (2H, H^ar^), 7.59 s (1H, NH), 7.41 t (2H, *J* = 7.5 Hz, H^ar^), 7.37–7.29 m (2H, H^ar^), 4.68 dm (1H, ^1^ *J*_HF_ = 49 Hz, CH-F), 4.40–4.31 m (2H, CHCH_2_O), 4.25 m (2H, CHCH_2_O + CHN), 3.06 t (2H, *J* = 7.7.Hz, C^6^H_2_N), 2.19 m (1H, *J* = 14 Hz, C^5^H/C^3^H), 1.98–1.91 m (2H, C^3^H_2_/C^5^H_2_), 1.85 m (1H, *J* = 14 Hz, C^5^H/C^3^H) ppm. ^13^C­{^1^H}­NMR (101 MHz, CD_3_CN) δ = 175.2 (COO), 158.0 (NCOO), 144.9 (C_q_), 142.1 (C_q_), 128.9 (CH), 128.3 (CH), 126.2 (CH), 121.1 (CH), 90.3 d (^1^ *J*_CF_ = 167 Hz, C^4^H–F), 67.7 (CH_2_O), 51.4 (C^2^HN), 47.9 (CH), 37.2 (NC^6^H_2_), 37.1 d (^2^ *J*_CF_ = 21 Hz, C^5^H_2_/C^3^H_2_), 33.0 d (^2^ *J*_CF_ = 20 Hz, C^3^H_2_/C^5^H_2_). ^19^F NMR (376 MHz, D_2_O): δ = −187.6 ddddd (*J* = 50, 38, 32, 18, and 13 Hz). HRMS (ESI-TOF) *m*/*z*: [M + H]^+^ calcd for C_21_H_24_FN_2_O_4_ 387.1715; found 387.1710.

### Compound **15**

Epimer 1: (2*S*,4*S*)-BocNH­(CH_2_)_2_CHFCH_2_CH­(NHFmoc)­CO_2_H. Compound **14**–epimer 1 (TFA salt, 156 mg, 0.312 mmol) was suspended in 1:1 aq. dioxane (15 mL), and NaHCO_3_ (180 mg, 2.14 mmol) was added, followed by Boc_2_O (218 mg, 1.0 mmol), and the mixture was stirred for several hours at room temperature. The precipitate did not disappear; the pH of the reaction mixture was measured to be 7.3. Saturated aq. NaHCO_3_ (0.9 mL) was added; the pH became 8.3. Boc_2_O (150 mg, 0.668 mmol) was added to the reaction mixture, and the mixture was stirred overnight at room temperature. Yet, the precipitate did not disappear completely. Aq. CH_3_CN (1:1, 25 mL) was added to the reaction mixture, followed by 1.6 mL of sat. aq. NaHCO_3_ and Boc_2_O (200 mg, 0.917 mmol); the precipitate disappeared. The total volume of the reaction mixture was about 50 mL. The reaction mixture was lyophilized and subjected to flash chromatography on RP-C18 (35 g). Aq. buffer was prepared. 0.73 g of *N*,*N*-dimethyl-*N*-ethylamine (10 mmol) was dissolved in water (1.0 L), and 0.6 mL of AcOH was added (10 mmol), followed by AcOH (ca. 0.3 mL), until the pH became 3.5. The lyophilized reaction mixture was dissolved in a minimum amount of aq. MeCN, the pH was adjusted to 3 by careful addition of AcOH, and the solution was applied onto the prepacked column (with 1:1 acetonitrile–aq. buffer solution, pH = 3.5). Elution with a 1:1 to 1:2 gradient (aq. buffer-acetonitrile) afforded ca. 100 mL of the pooled fractions containing the product. This solution was lyophilized, and the residue (colorless powder) was dissolved in aq. MeCN, filtered, and lyophilized once more. The residue (225 mg) was distributed between EtOAc and 1 M aq. KHSO_4_; the organic layer was separated, dried, and evaporated; and the residue was recrystallized from EtOAc–cyclohexane to afford 130 mg (86%) of compound **15**–epimer 1 as a colorless foam, which decomposes with melting upon heating above 140 °C. [α]_25_^D^ = +11 (*c* = 1.2, CHCl_3_). ^1^H NMR (400 MHz, CD_3_OD) δ = 7.85 m (2H, H^ar^), 7.67 ddd (2H, *J* = 6.4, 5.0, and 2.6 Hz, H^ar^), 7.40–7.35 m (2H, H^ar^), 7.33–7.27 m (2H, H^ar^), 4.65 dm (1H, ^1^ *J*_HF_ = 52 Hz, CH-F), 4.33 m (2H, CHCH_2_O), 4.26–4.14 m (2H, CHCH_2_O + CHN), 3.17 t (2H, *J* = 6.9 Hz, C^6^H_2_N), 2.19 m (1H, C^5^H/C^3^H), 1.87–1.64 m (3H, C^3^H_2_/C^5^H_2_), 1.41 s (CMe_3_) ppm. ^13^C­{^1^H} NMR (101 MHz, CD_3_OD) δ = 180.3 (COO), 170.4 (NCOO), 158.4 (NCOO), 145.5/145.3 (C_q_), 142.6 (C_q_), 128.7 (CH), 128.2 (CH), 126.3 (CH), 120.9 (CH), 90.7 d (^1^ *J*_CF_ = 168 Hz, C^4^H–F), 80.1 (C_q_-O), 67.9 (CH_2_O), 54.6 (C^2^HN), 48.5 (CH), 39.6 d (^2^ *J*_CF_ = 21 Hz, C^5^H_2_/C^3^H_2_), 37.6 (NC^6^H_2_), 36.8 d (^2^ *J*_CF_ = 21 Hz, C^5^H_2_/C^3^H_2_), 28.8 (CMe_3_) ppm. C_26_H_31_FN_2_O_6_, 486.2166. ^19^F NMR (376 MHz, D_2_O) δ = −185.0 ddq (*J* = 50, 32, and 16 Hz). HRMS (ESI-TOF) *m*/*z*: [M + Na]^+^ calcd for C_26_H_31_FN_2_O_6_Na 509.2059; found 509.2054.

### Compound **15**

Epimer 2: (2*S*,4*R*)-BocNH­(CH_2_)_2_CHFCH_2_CH­(NHFmoc)­CO_2_H. Compound **14**–epimer 2 (TFA salt, 135 mg, 0.27 mmol) was dissolved in 1:1 aq. dioxane (10 mL), and NaHCO_3_ (140 mg, 1.67 mmol) was added, followed by Boc_2_O (218 mg, 1.00 mmol), and the mixture was stirred for several hours at room temperature. The precipitate disappeared; the pH of the reaction mixture was measured to be 7.6. Saturated aq. NaHCO_3_ (0.9 mL) was added; the pH became 8.3. Boc_2_O (120 mg, 0.526 mmol) was added to the reaction mixture, and the mixture was stirred overnight at room temperature. The reaction mixture was lyophilized and subjected to flash chromatography on RP-C18 (35 g). Aq. buffer was prepared. 0.73 g of *N*,*N*-dimethyl-*N*-ethylamine (10 mmol) was dissolved in water (1.0 L), and 0.6 mL AcOH was added (10 mmol), followed by AcOH (ca. 0.3 mL), until the pH became 3.5. The lyophilized reaction mixture was dissolved in a minimum amount of aq. MeCN, the pH was adjusted to 3 by careful addition of AcOH, and the solution was applied onto the prepacked column (with 1:1 acetonitrile–aq. buffer solution, pH = 3.5). Elution with a 1:1 to 1:2 gradient (aq. buffer–acetonitrile) afforded ca. 50–70 mL of the pooled fractions containing the product. This solution was lyophilized, and the residue (colorless powder) was dissolved in aq. MeCN, filtered, and lyophilized once more. The residue (141 mg) was distributed between EtOAc and 1 M aq. KHSO_4_; the organic layer was separated, dried, and evaporated; and the residue was recrystallized from EtOAc–cyclohexane to afford 113 mg (91%) of compound **15**–epimer 2 as a colorless amorphous solid, which decomposes with melting upon heating above 124 °C. [α]_25_^D^ = +4.7 (*c* = 1.3, CHCl_3_). ^1^H NMR (400 MHz, CD_3_OD) δ = 7.79 dt (2H, *J* = 7.6 and 0.9 Hz, H^ar^), 7.73–7.63 m (2H, H^ar^), 7.43–7.35 m (2H, H^ar^), 7.31 td (2H, *J* = 7.5 and 1.2 Hz, H^ar^), 4.71 dd (1H, *J*_HF_ = 49 and 6.1 Hz, CH-F), 4.34 dd (2H, *J* = 7.0 and 3.8 Hz, CHCH_2_O), 4.22 t (1H, *J* = 7.0 Hz, CHCH_2_O), 4.13 t (1H, *J* = 6.7 Hz, CHN), 3.18 m (2H, C^6^H_2_N), 2.13–1.99 m (2H, C^5^H_2_/C^3^H_2_), 1.83–1.70 m (2H, C^3^H_2_/C^5^H_2_), 1.39 s (CMe_3_) ppm. ^13^C­{^1^H}­NMR (101 MHz, CD_3_OD) δ = 178.1 (COO), 158.4/158.1 (NCOO), 145.5/145.2 (C_q_), 142.6 (C_q_), 128.8 (CH), 128.2 (CH), 126.3 (CH), 120.9 (CH), 91.1 d (^1^ *J*_CF_ = 167 Hz, C^4^H–F), 79.9 (C_q_-O), 67.9 (CH_2_O), 54.6 (C^2^HN), 48.4 (CH), 39.7 d (^2^ *J*_CF_ = 21 Hz, C^5^H_2_/C^3^H_2_), 37.6 (NC^6^H_2_), 36.2 d (^2^ *J*_CF_ = 21 Hz, C^3^H_2_/C^5^H_2_), 28.8 (CMe_3_) ppm. ^19^F NMR (376 MHz, D_2_O): δ = −183.5 ddt (*J* = 51, 33, and 19 Hz). HRMS (ESI-TOF) *m*/*z*: [M + Na]^+^ calcd for C_26_H_31_FN_2_O_6_Na 509.2059; found 509.2056.

## Supplementary Material



## Data Availability

The data underlying this study are available in the published article and its Supporting Information.

## Notes

LC-MS data, copies of ^1^H and ^13^C NMR spectra of compounds **2**–**7** and **8**–**15** (epimers 1 and 2), and details of X-ray analysis of compound **13**–epimer 1 (PDF)
