# Supplementary material for: Uniform Preparation of (4R)- and (4S)‑Fluoro‑l‑lysines from One Precursor: Retrosynthesis Software Approach and the Route of a Chemist
Source: J Org Chem. 2025 Nov 27;90(49):17478–90. doi: 10.1021/acs.joc.5c02332 (PMC12706783; doi:10.1021/acs.joc.5c02332)
Supplement: Supplementary file 1 [file jo5c02332_si_001.pdf]

Supplementary Information

Uniform preparation of 4*R*- and 4*S*-fluoro-L-lysines from one precursor: retrosynthesis software approach and the route of a chemist

Vladimir N. Belov[1,2,*], Christopher Golz[3]

[1] Department of NanoBiophotonics, Max Planck Institute for Multidisciplinary Sciences (MPI NAT), Am Fassberg 11, 37077 Göttingen, Germany

[2] Facility for Synthetic Chemistry, MPI NAT, Am Fassberg 11, 37077 Göttingen, Germany

[3] Institut für organische und biomolekulare Chemie der Georg-August-Universität, Tammannstrasse 2, 37077 Göttingen, Germany

*Corresponding author:

E-mail: vladimir.belov@mpinat.mpg.de

Tel.: +49 (0)551 201 2530

Fax: +49 (0)551 201 2506

[a]ORCID iD: orcid.org/0000-0002-7741-4653

[b]ORCID iD: orcid.org/0009-0001-8865-3254

Table of Contents

Copies of LC-MS traces and NMR spectra S3-S43

Crystallographic data (X-ray analysis) S44

Compound 2: (S)-CH_2=$CHCOCH_2CH(NHCO_2CH_2C_6H_5)CO_2CH_2C_6H_5$.

Sample : **vb21121**

Gradient: A 20.0 % B 80.0 % ----> A 100.0 % B 0.0 % T = 7 Min.

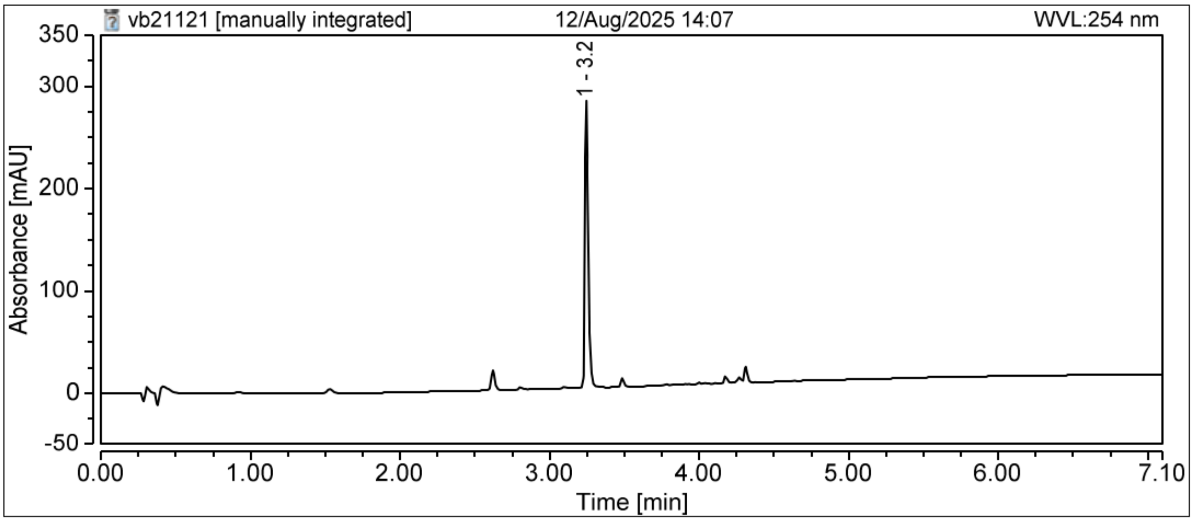

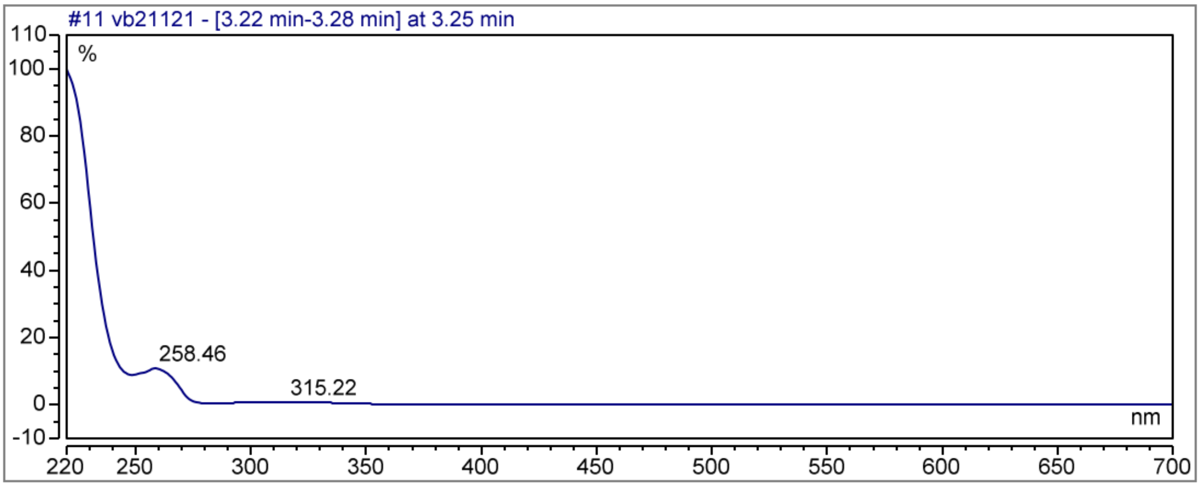

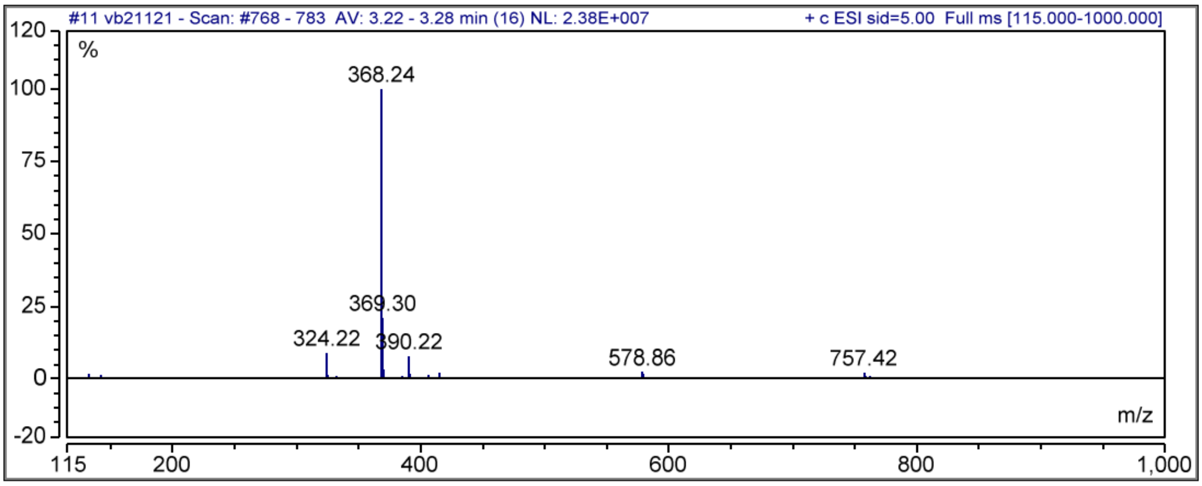

^{1}H NMR (400 MHz, Chloroform-*d*) δ 7.43 – 7.25 (m, 10H), 6.32 (ddd, *J* = 17.7, 10.2, 1.5 Hz, 1H), 6.23 (d, *J* = 17.6 Hz, 1H), 5.90 (dd, *J* = 10.2, 1.5 Hz, 1H), 5.82 (d, *J* = 8.7 Hz, 1H), 5.21 – 5.13 (m, 2H), 5.11 (d, *J* = 1.5 Hz, 2H), 4.70 (p, *J* = 4.1 Hz, 1H), 3.40 (dd, *J* = 18.2, 4.3 Hz, 1H), 3.18 (dd, *J* = 18.1, 4.2 Hz, 1H).

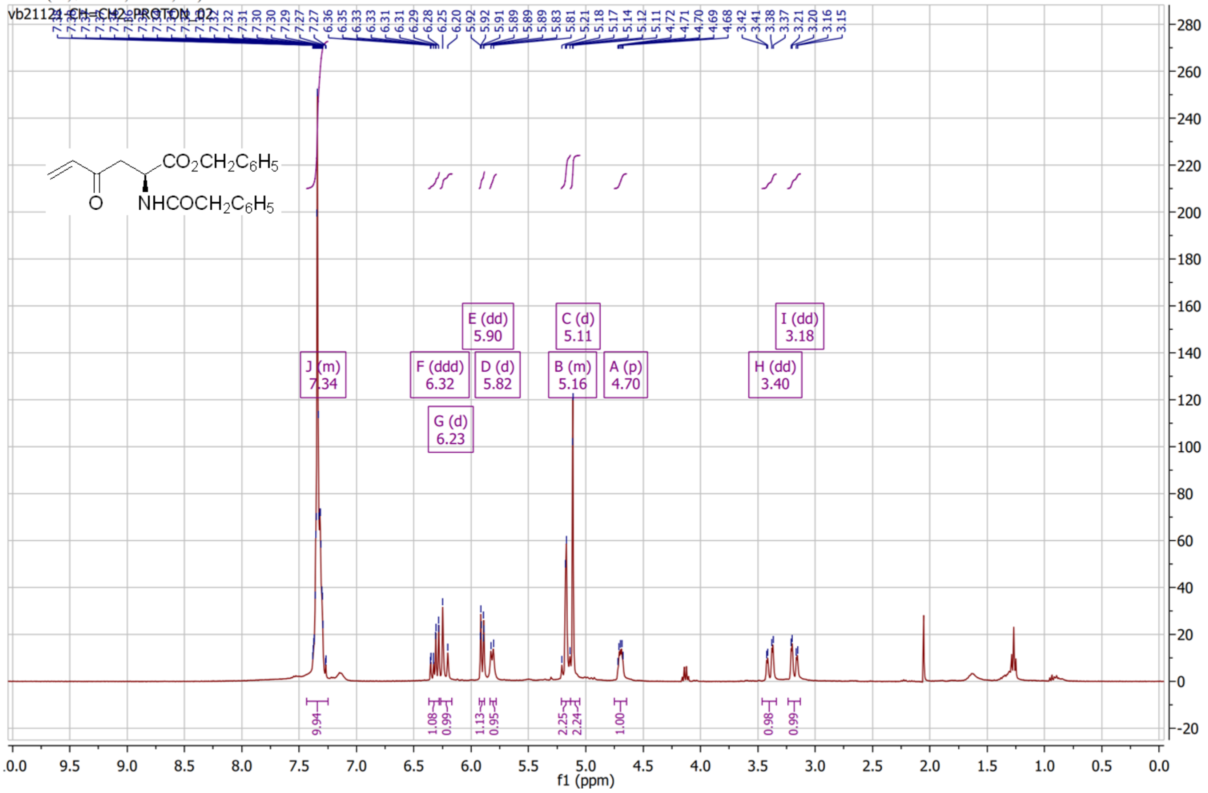

$^{13}C\{^{1}H\}$-NMR (101 MHz ^{13}C) spectrum in $CDCl_3$

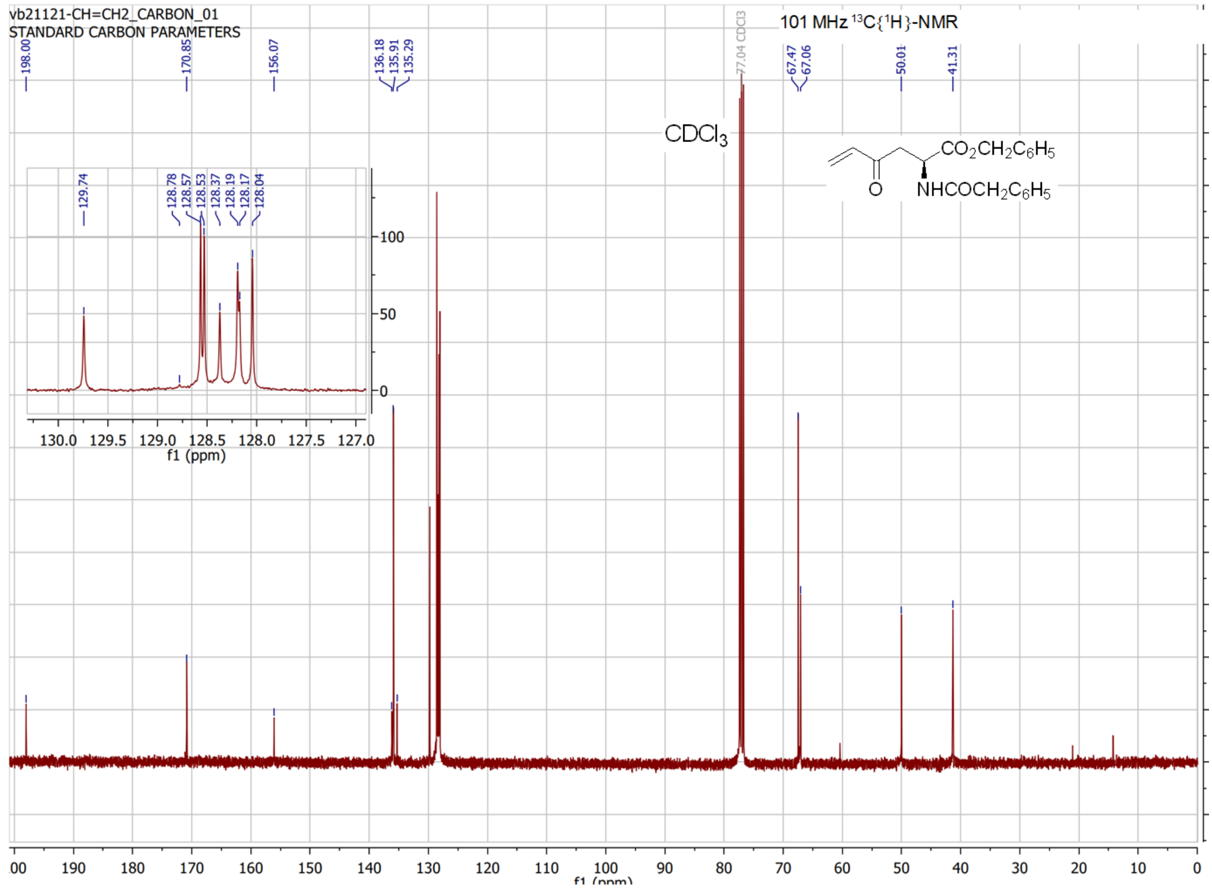

Compound 3: (S)-$N_3CH_2CH_2COCH_2CH(NHCO_2CH_2C_6H_5)CO_2CH_2C_6H_5$. NMR spectra in CD_3OD.

^{1}H NMR (400 MHz, Methanol-d_4) δ 7.40 – 7.21 (m, 10H), 5.19 – 5.09 (m, 2H), 5.08 (s, 2H), 4.65 (dd, J = 6.8, 5.4 Hz, 1H), 3.46 (td, J = 6.4, 2.6 Hz, 2H), 3.06 (dd, J = 17.7, 5.4 Hz, 1H), 2.97 (dd, J = 17.7, 6.7 Hz, 1H), 2.70 (td, J = 6.3, 1.4 Hz, 2H).

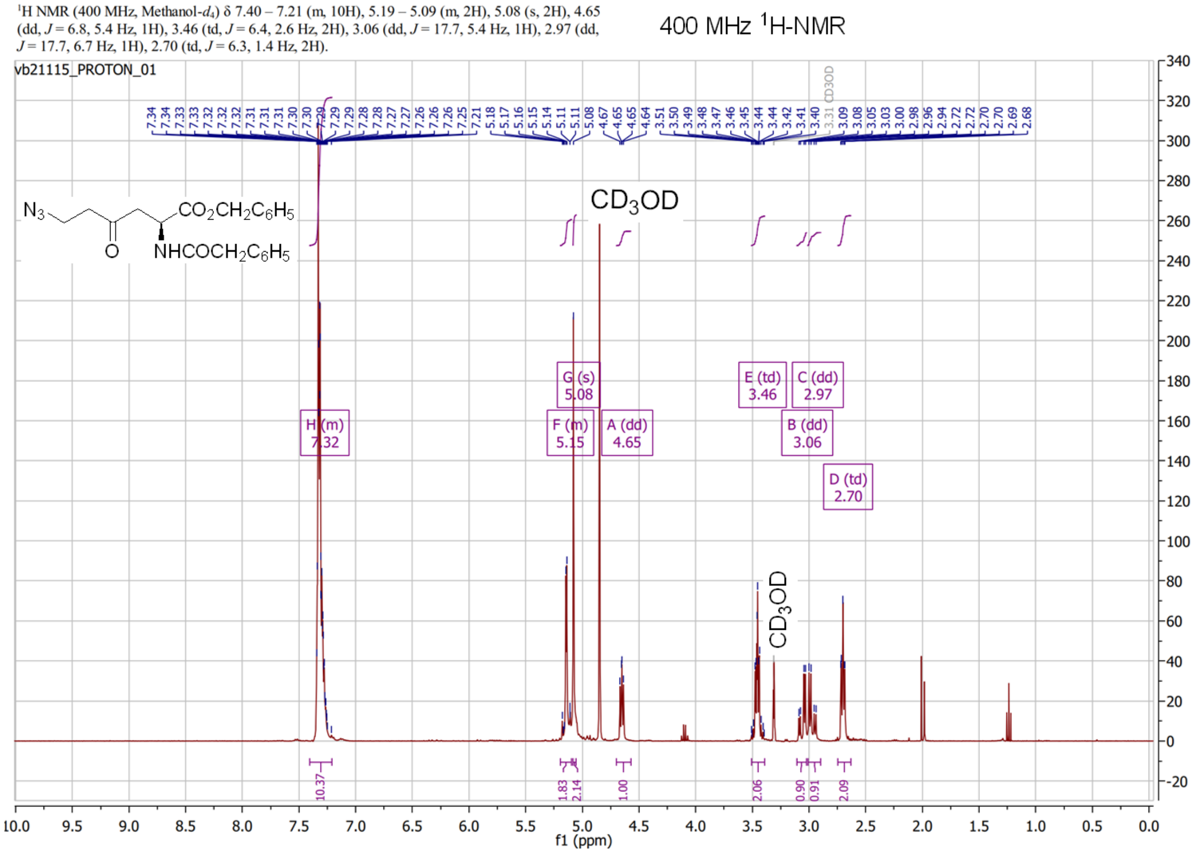

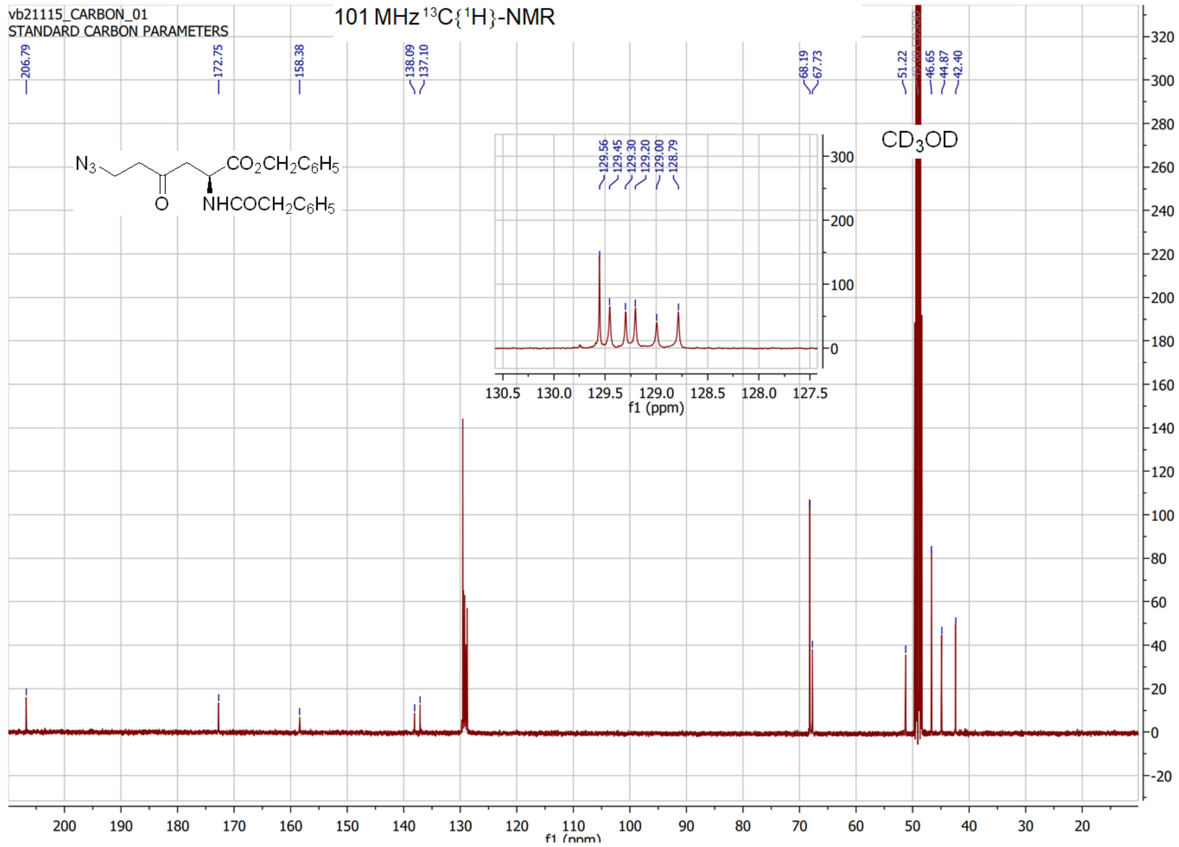

Compound 4: (S)-BocNHCH$_2$CH$_2$COCH$_2$CH(NHCO$_2$CH$_2$C$_6$H$_5$)CO$_2$CH$_2$C$_6$H$_5$.

Sample : vb21114

Gradient: A 20.0 % B 80.0 % **---->** A 100.0 % B 0.0 % T = 7 Min.

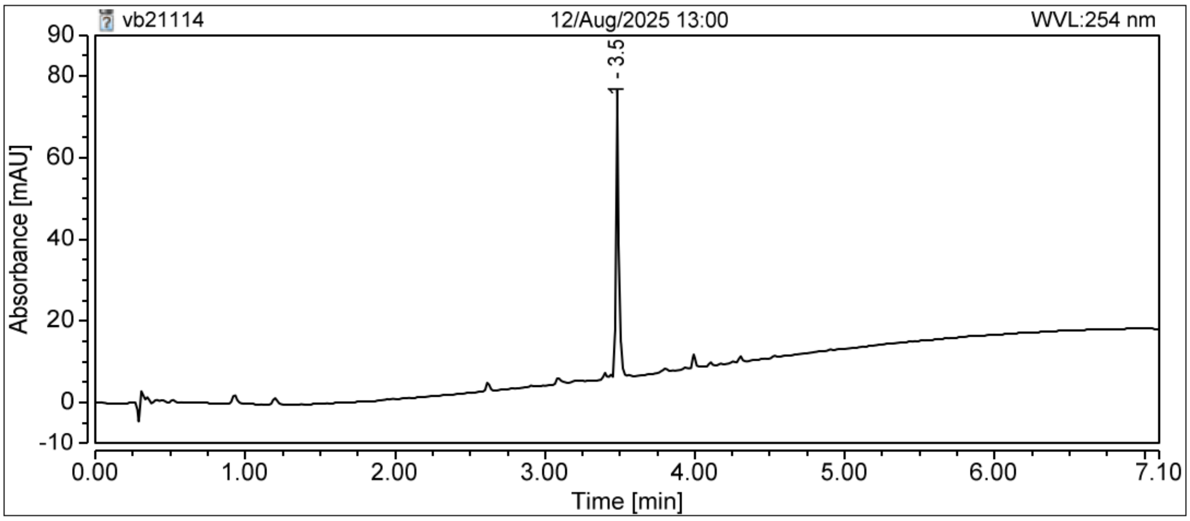

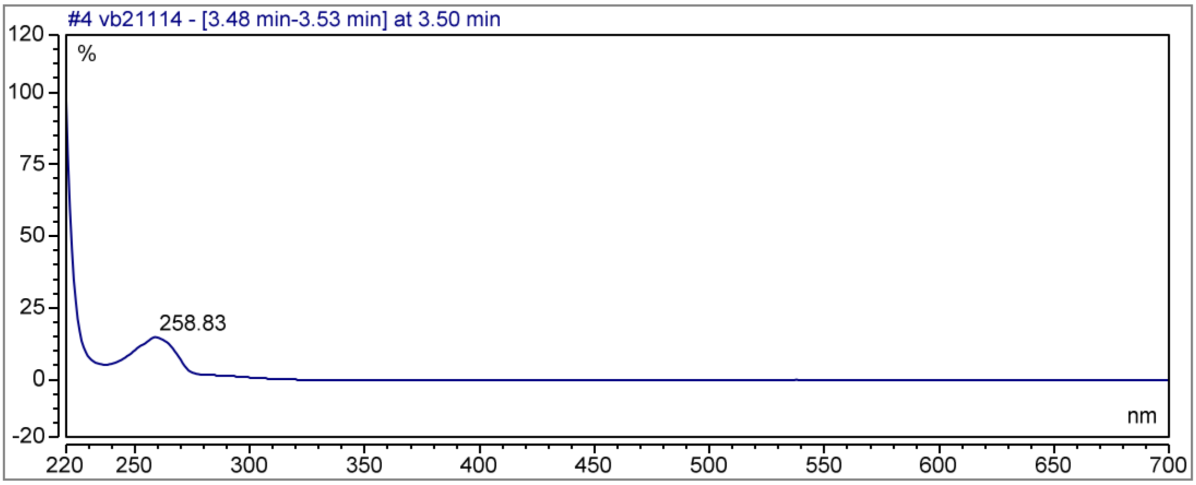

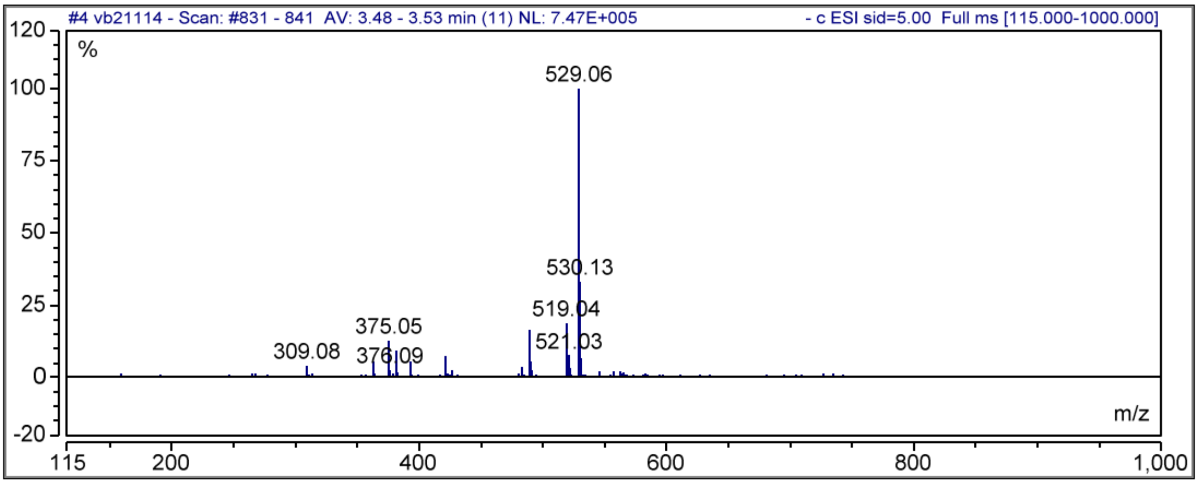

^{1}H-NMR (400 MHz) and ^{13}C{^{1}H}-NMR (101 MHz) spectra in CD_3OD.

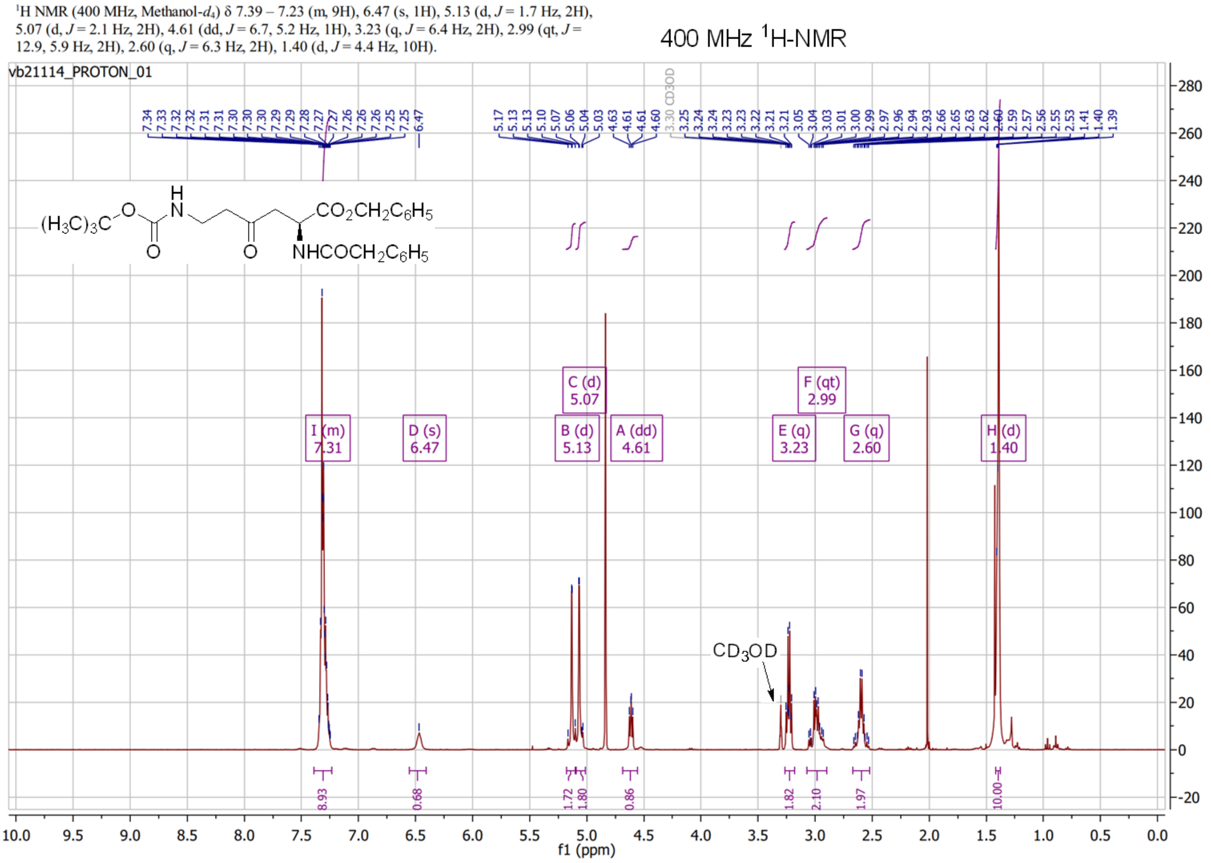

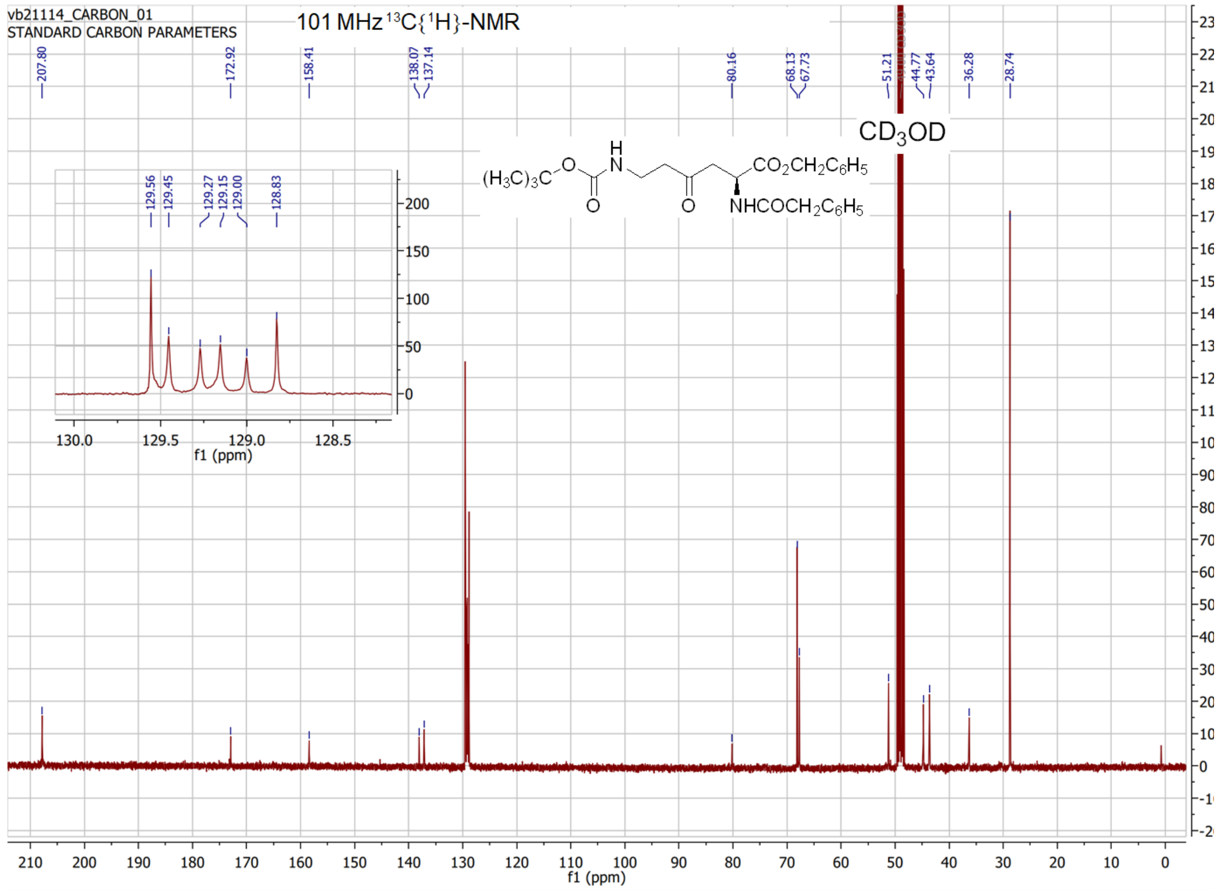

Compound 5: (S)-Boc$NHCH_2CH_2COCH_2CH(NH_2)CO_2H$.

Sample : **vb21116-2**

Gradient: A 2.0 % B 98.0 % ----> A 100.0 % B 0.0 % T = 7 Min.

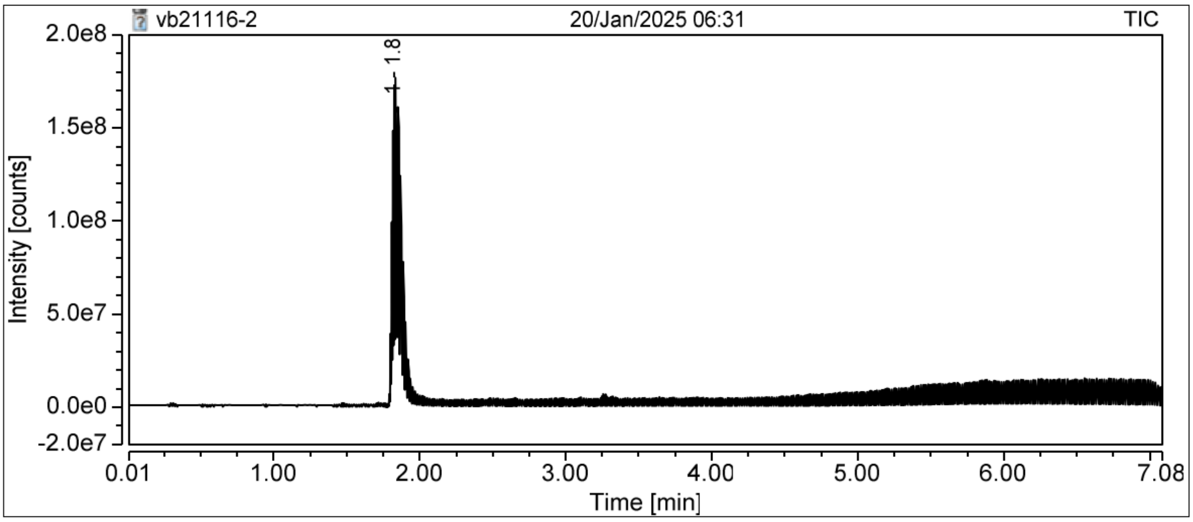

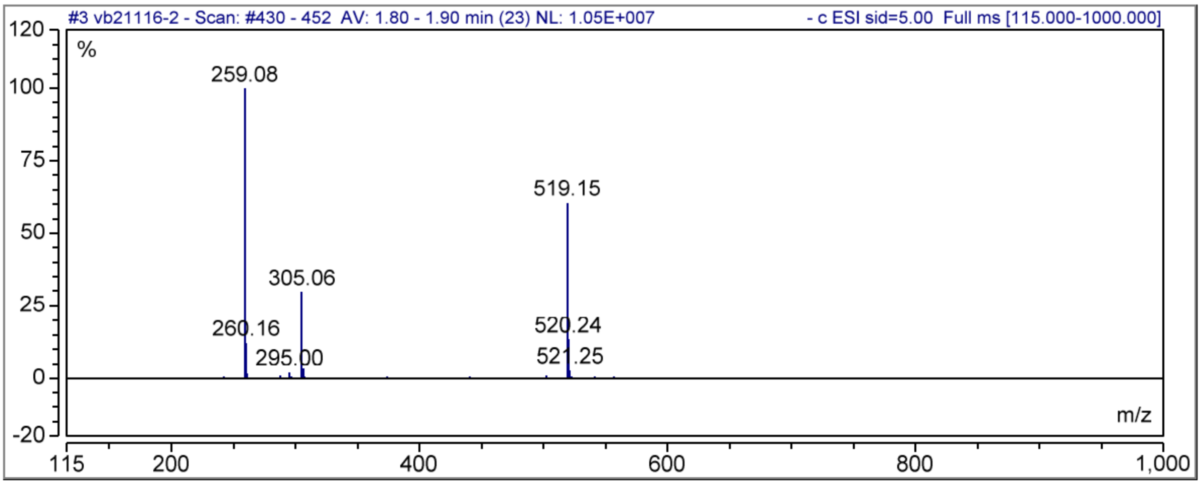

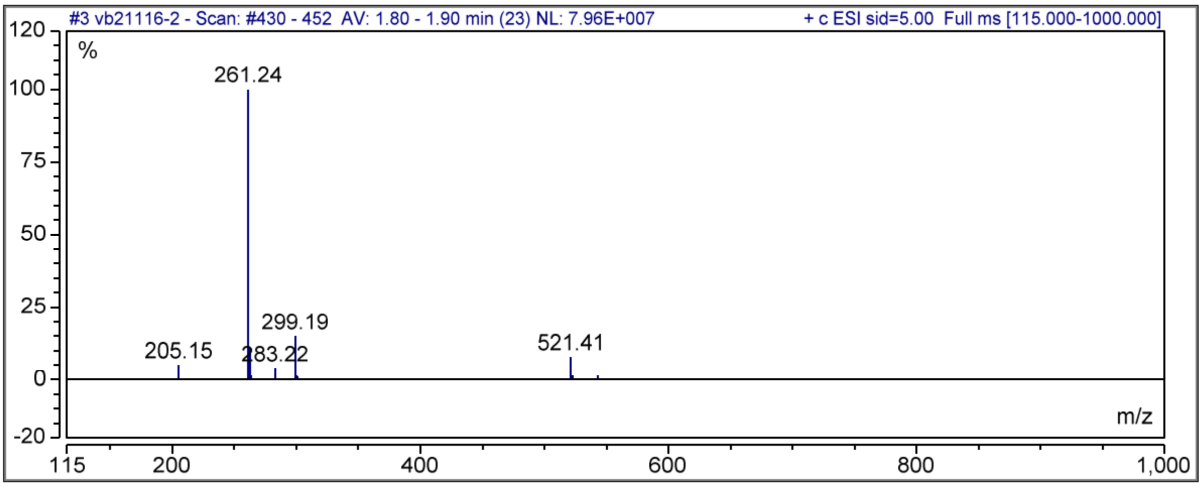

^{1}H-NMR (400 MHz) and ^{13}C{^{1}H}-NMR (101 MHz) spectra in D_2O.

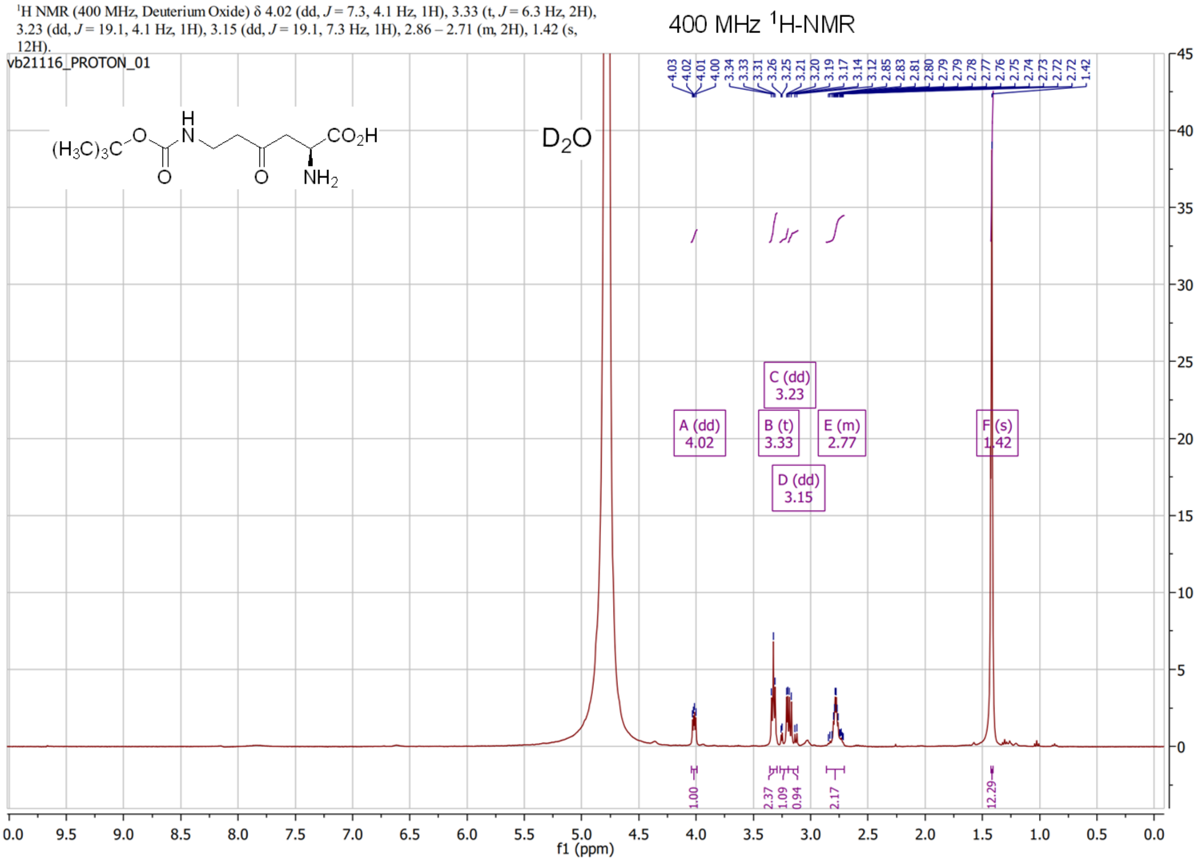

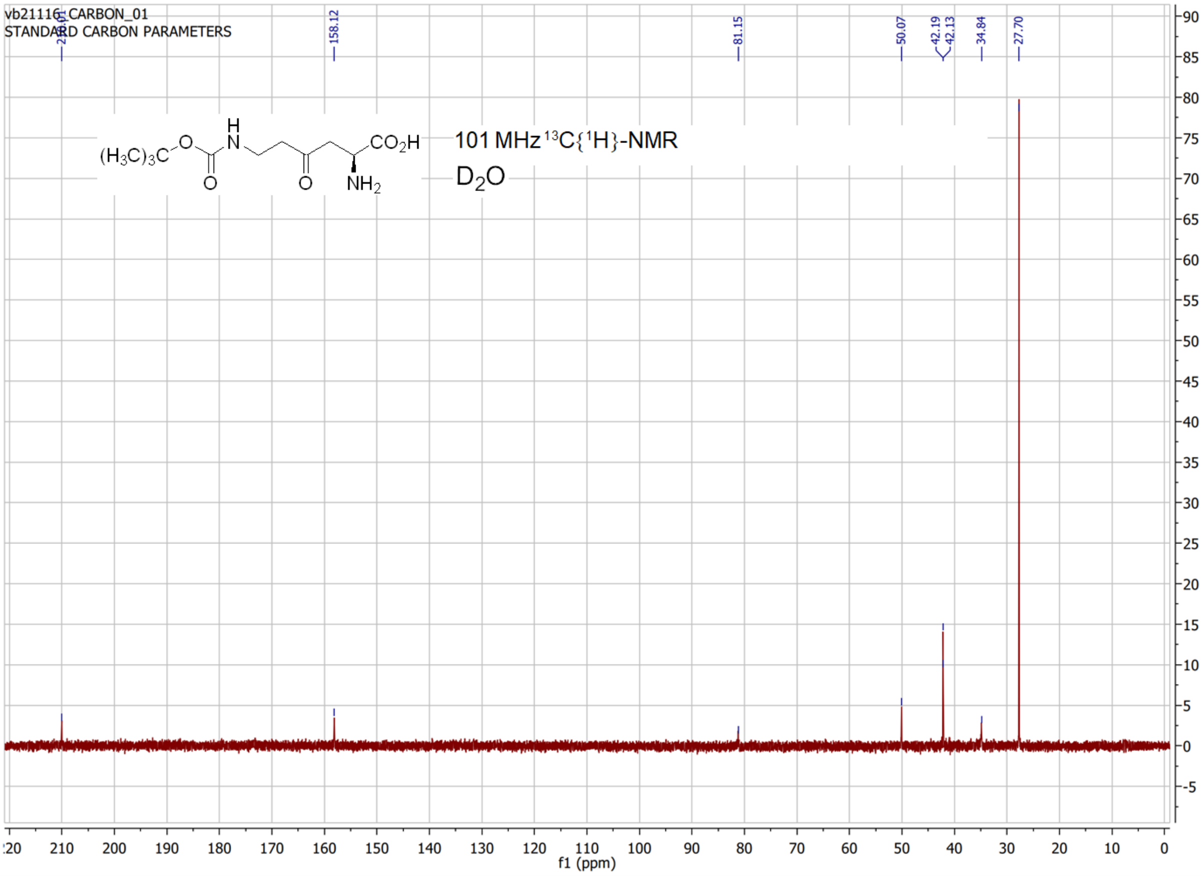

Compound 6: (*S*)-BocNHCH$_2$CH$_2$COCH$_2$CH(NHCO$_2$CH$_2$C$_6$H$_5$)CO$_2$H.

Sample : **vb21140**

Gradient: A 20.0 % B 80.0 % **---->** A 100.0 % B 0.0 % T = 7 Min.

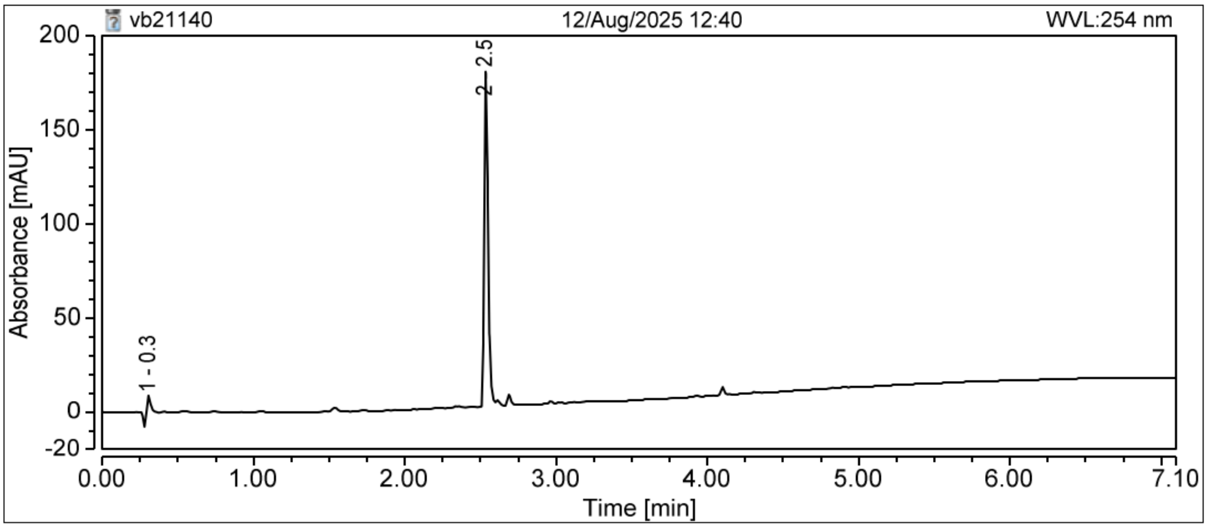

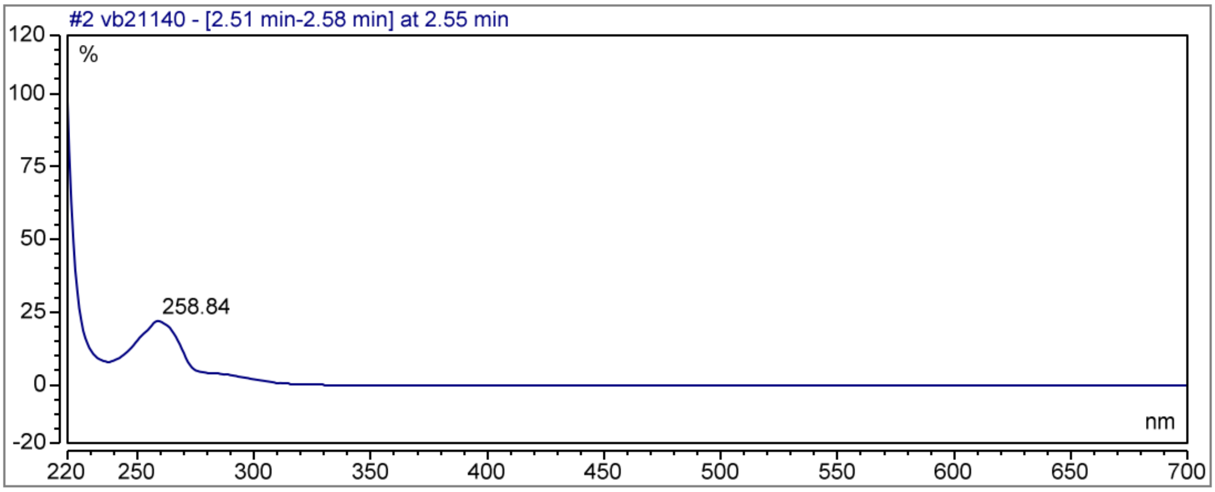

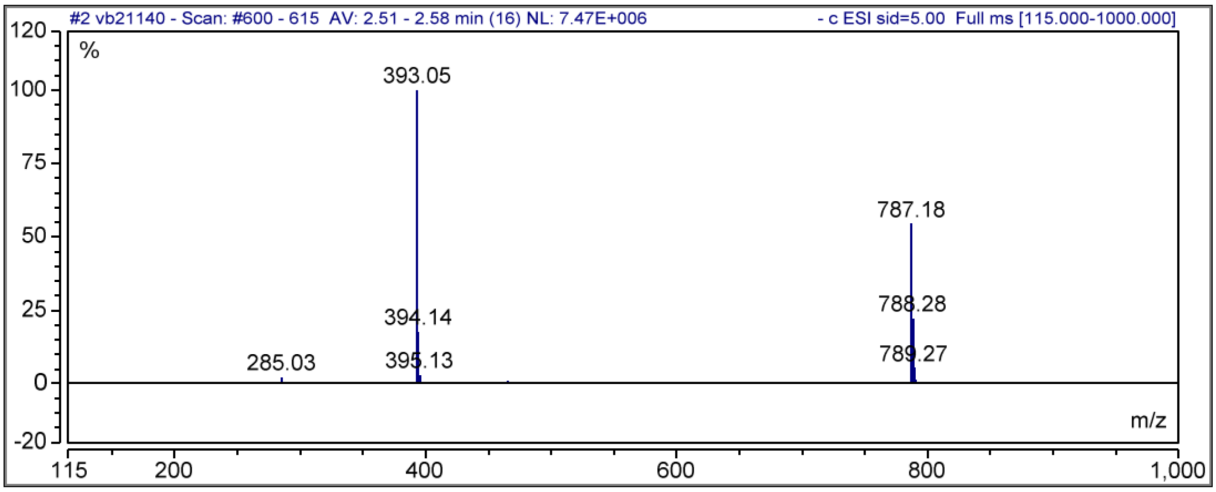

^{1}H-NMR (400 MHz) and $^{13}C\{^{1}H\}$-NMR (101 MHz) spectra in CD_3OD.

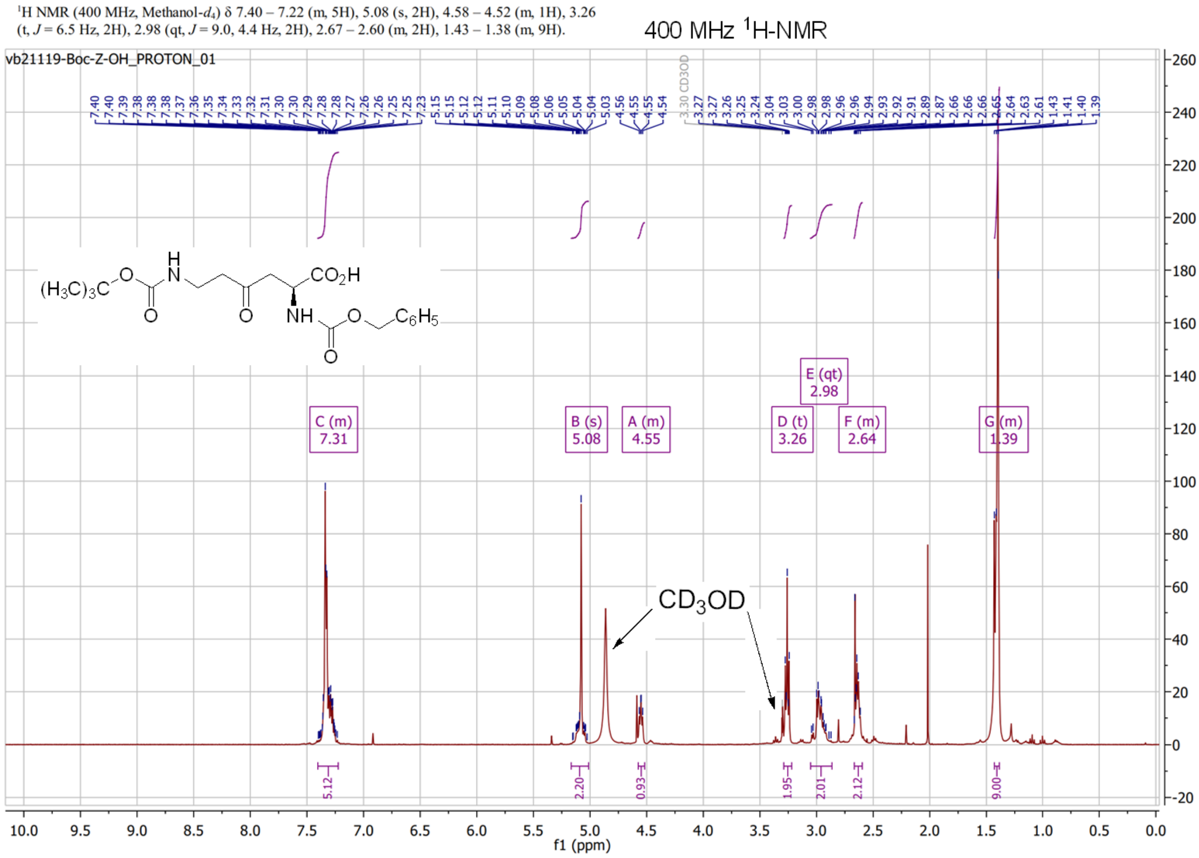

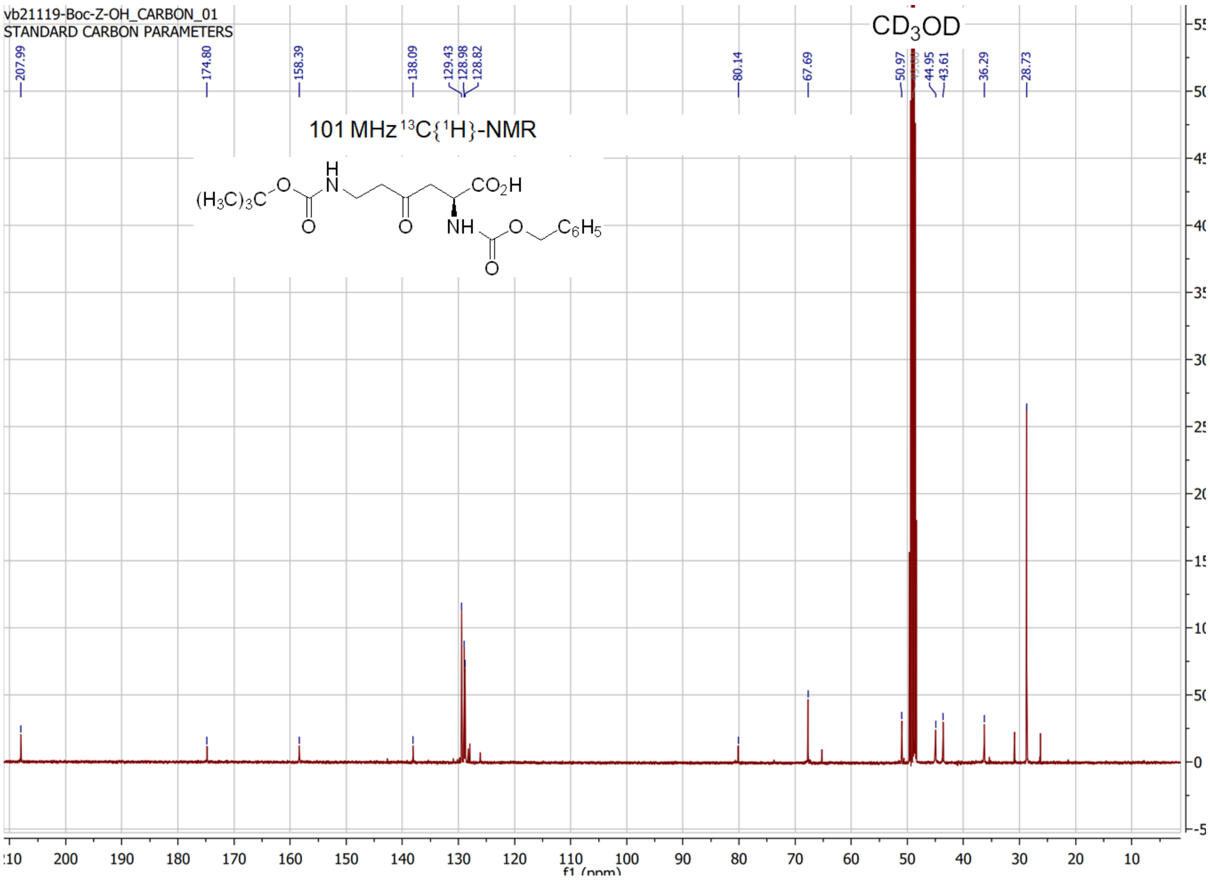

Compound 7: (*S*)-BocNHCH$_2$CH$_2$COCH$_2$CH(NHCO$_2$CH$_2$C$_6$H$_5$)CO$_2$Bu^t.

Sample : **vb21123-b**

Gradient: A 20.0 % B 80.0 % ----> A 100.0 % B 0.0 % T = 7 Min.

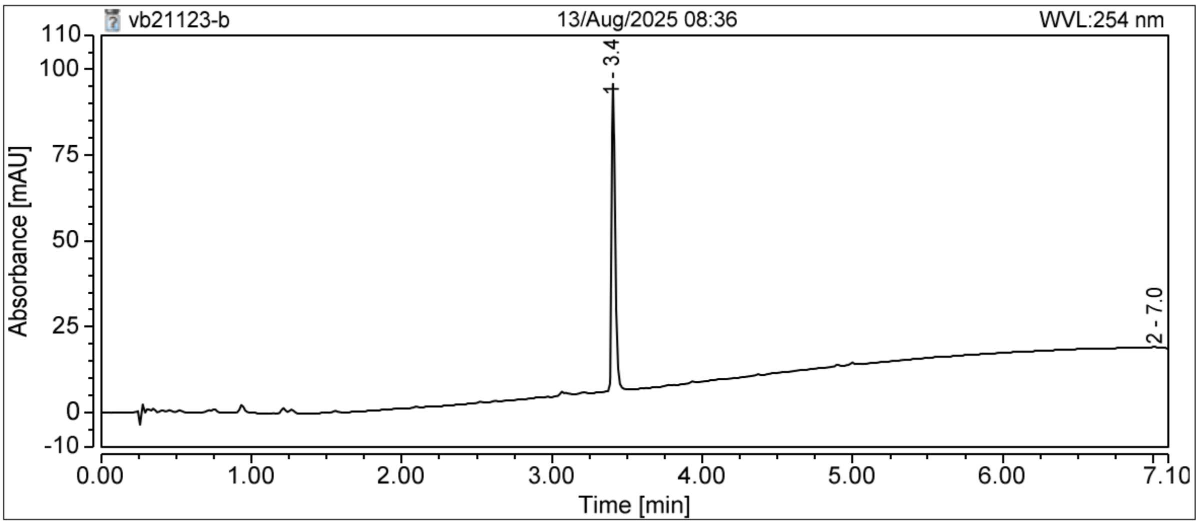

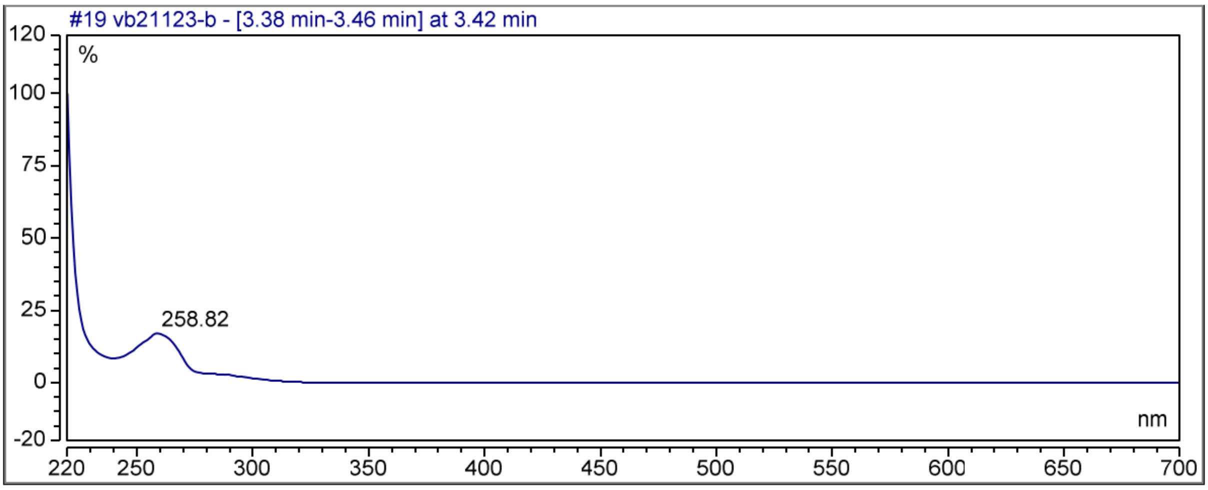

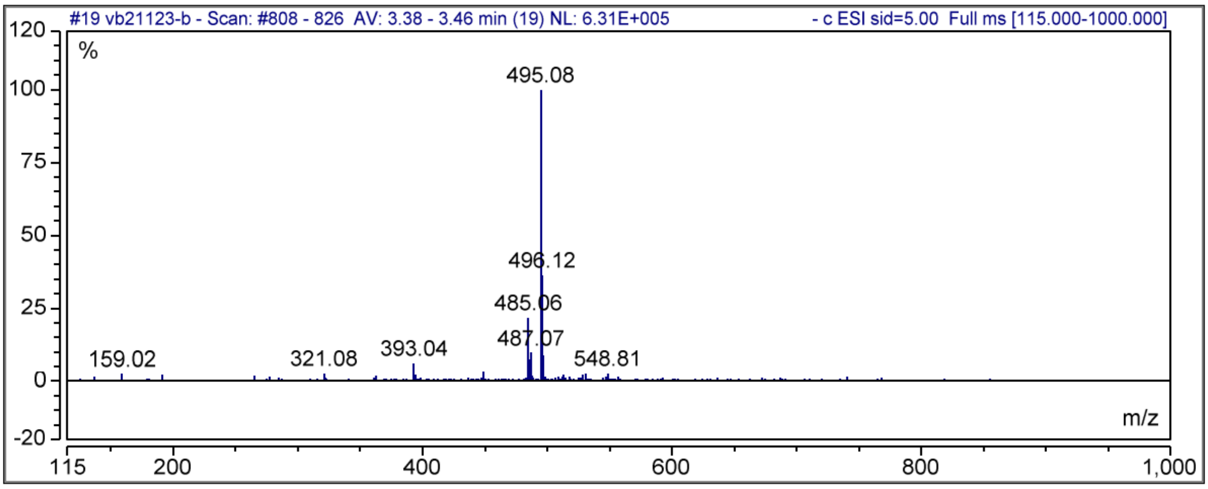

^{1}H-NMR (400 MHz) and $^{13}C\{^{1}H\}$-NMR (101 MHz) spectra in CD_3OD.

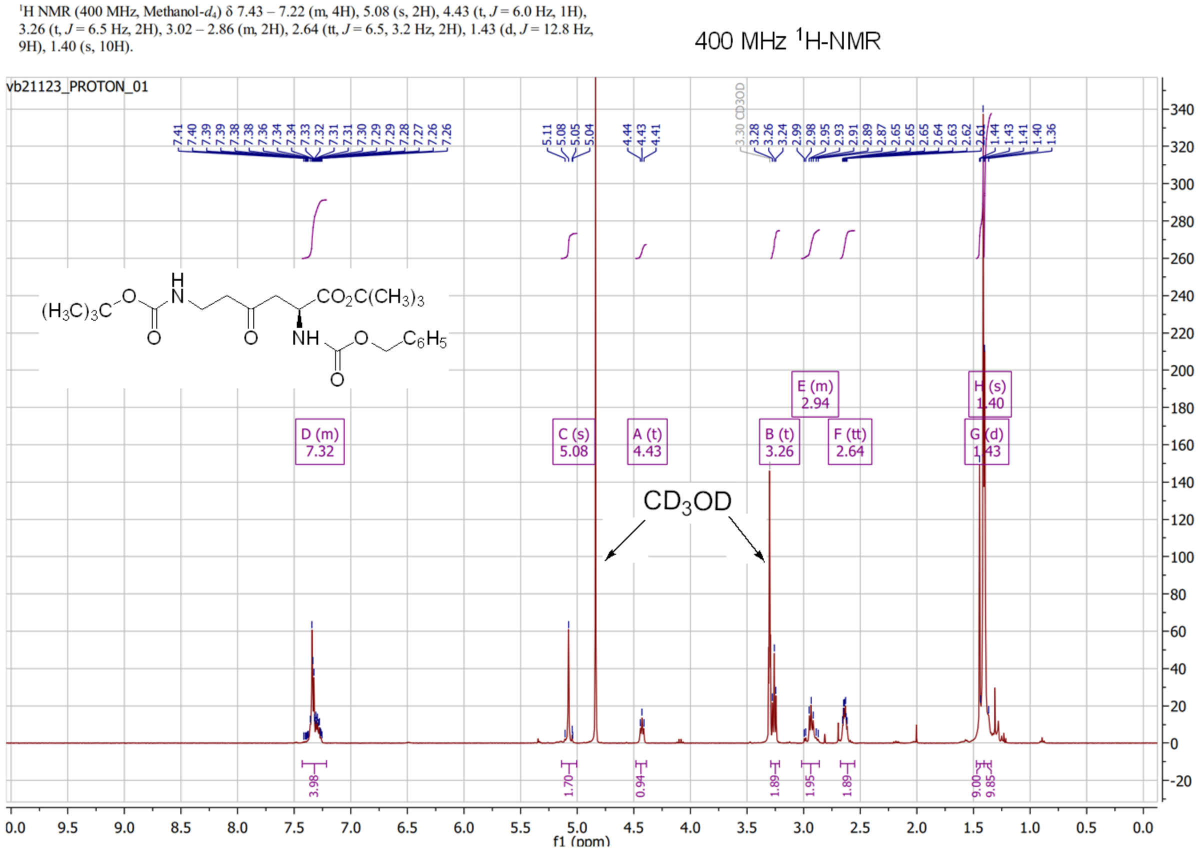

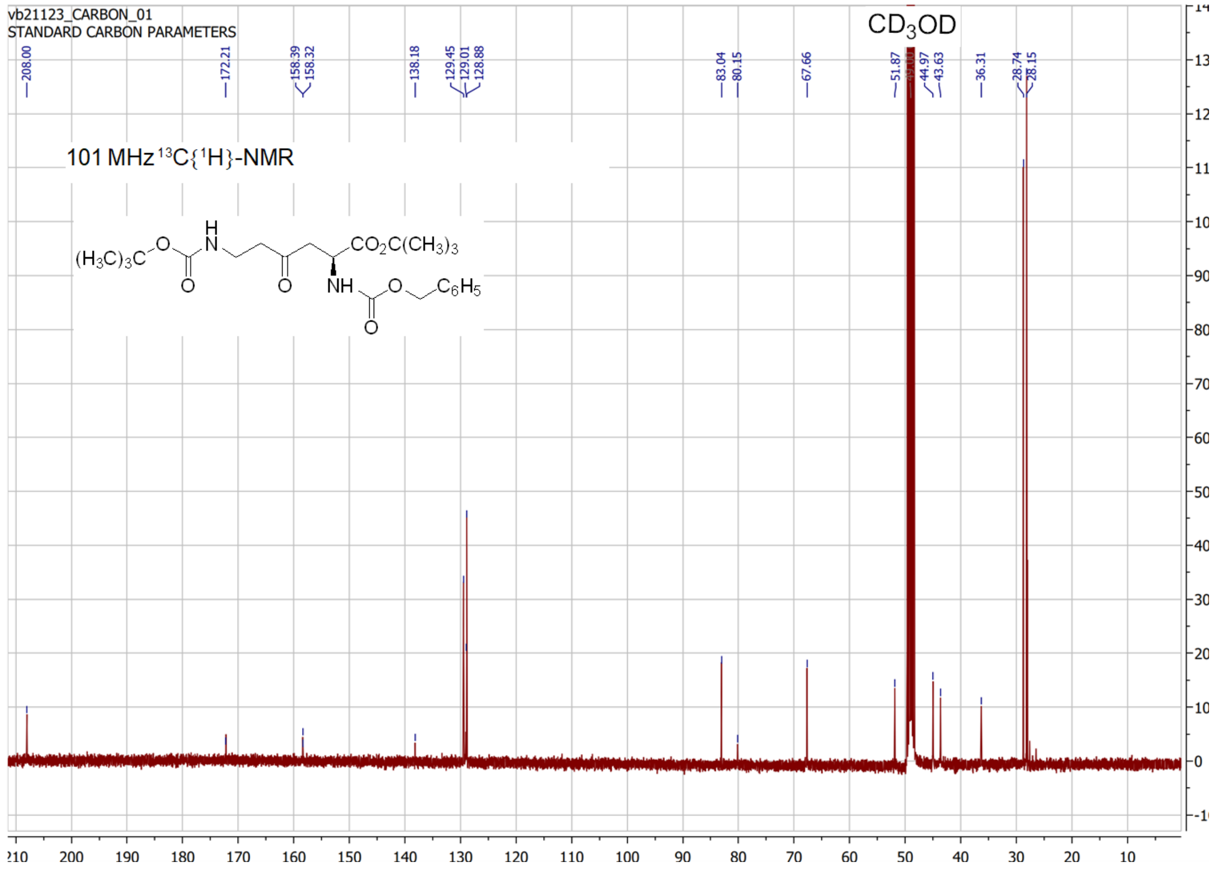

Compound 8 – epimer 1: (2*S*, 4*R*)-BocNH$(CH_2)_2$CH(OH)CH_2CH($NHCO_2CH_2C_6H_5$)CO_2Bu^t

Sample : **vb21124-1**

Gradient: A 20.0 % B 80.0 % ----> A 100.0 % B 0.0 % T = 7 Min.

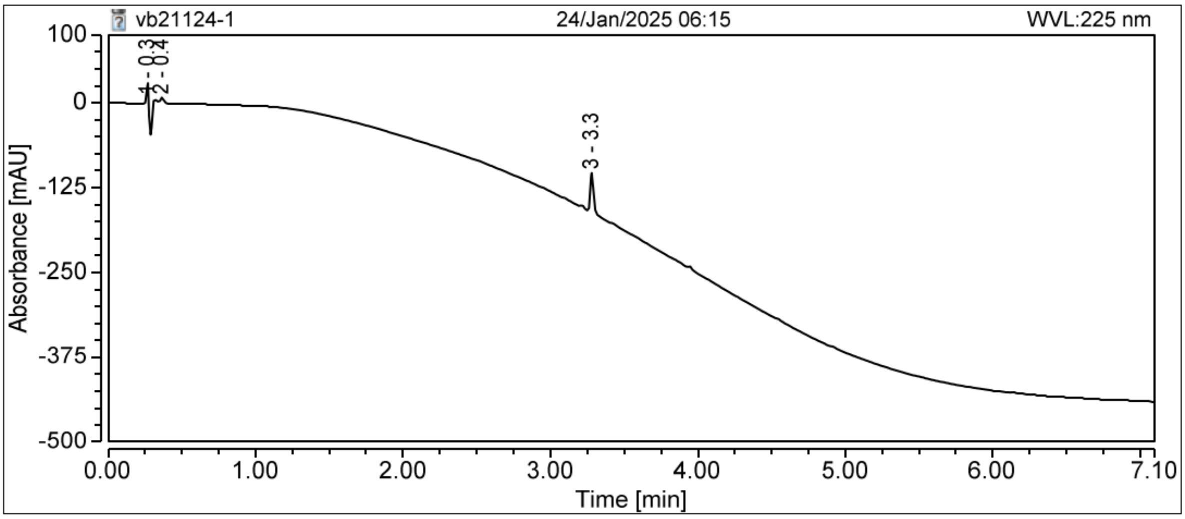

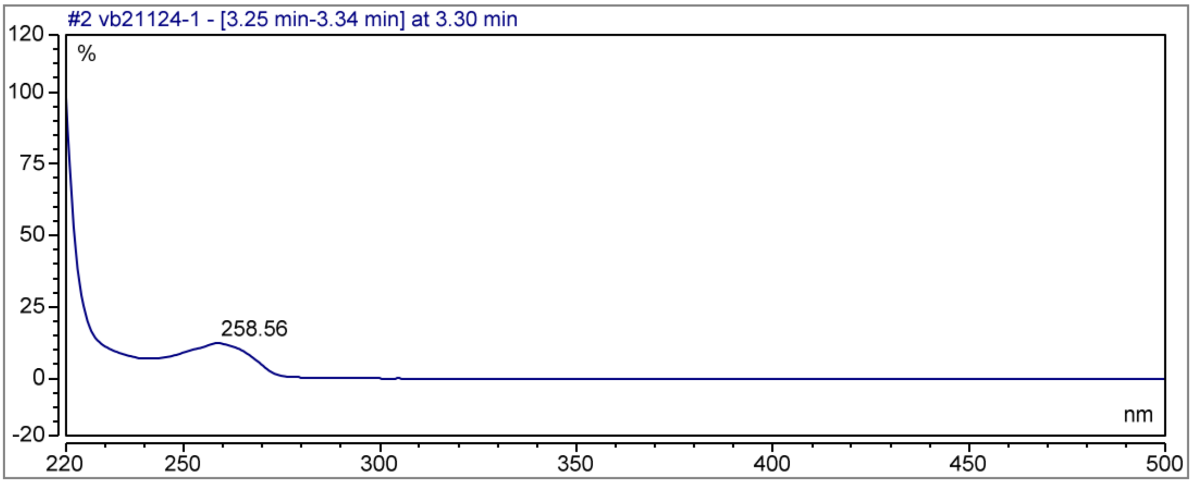

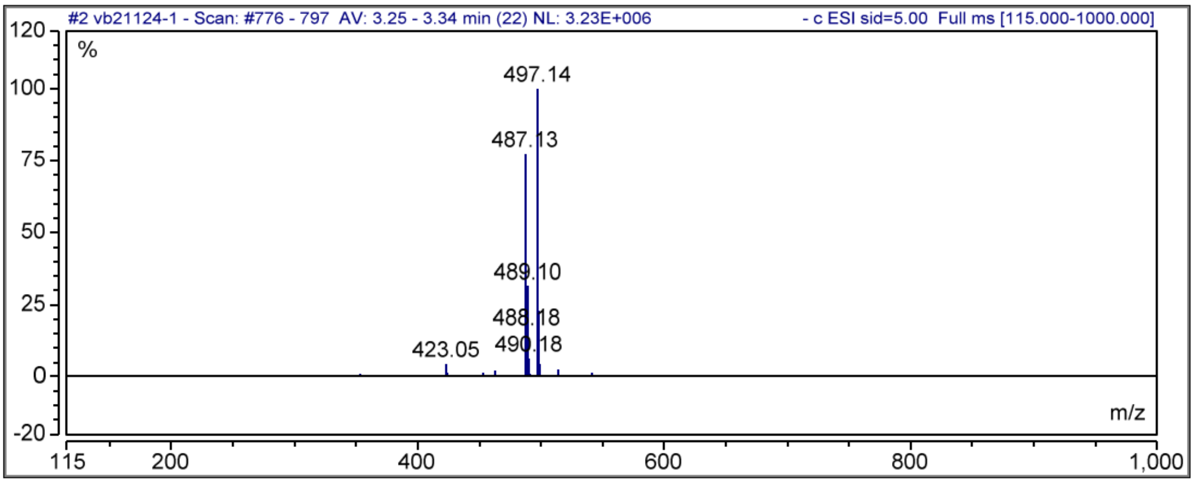

^{1}H-NMR (400 MHz) and ^{13}C{^{1}H}-NMR (101 MHz) spectra in CD_3OD

^{1}H NMR (400 MHz, Methanol-d_4) δ 7.44 – 7.22 (m, 4H), 5.09 (s, 2H), 4.29 (dd, J = 10.3, 3.8 Hz, 1H), 3.80 – 3.57 (m, 1H), 3.19 – 3.09 (m, 2H), 1.77 (dtd, J = 16.9, 14.0, 10.4 Hz, 2H), 1.65 – 1.51 (m, 2H), 1.45 (d, J = 1.9 Hz, 9H), 1.39 (d, J = 14.5 Hz, 9H).

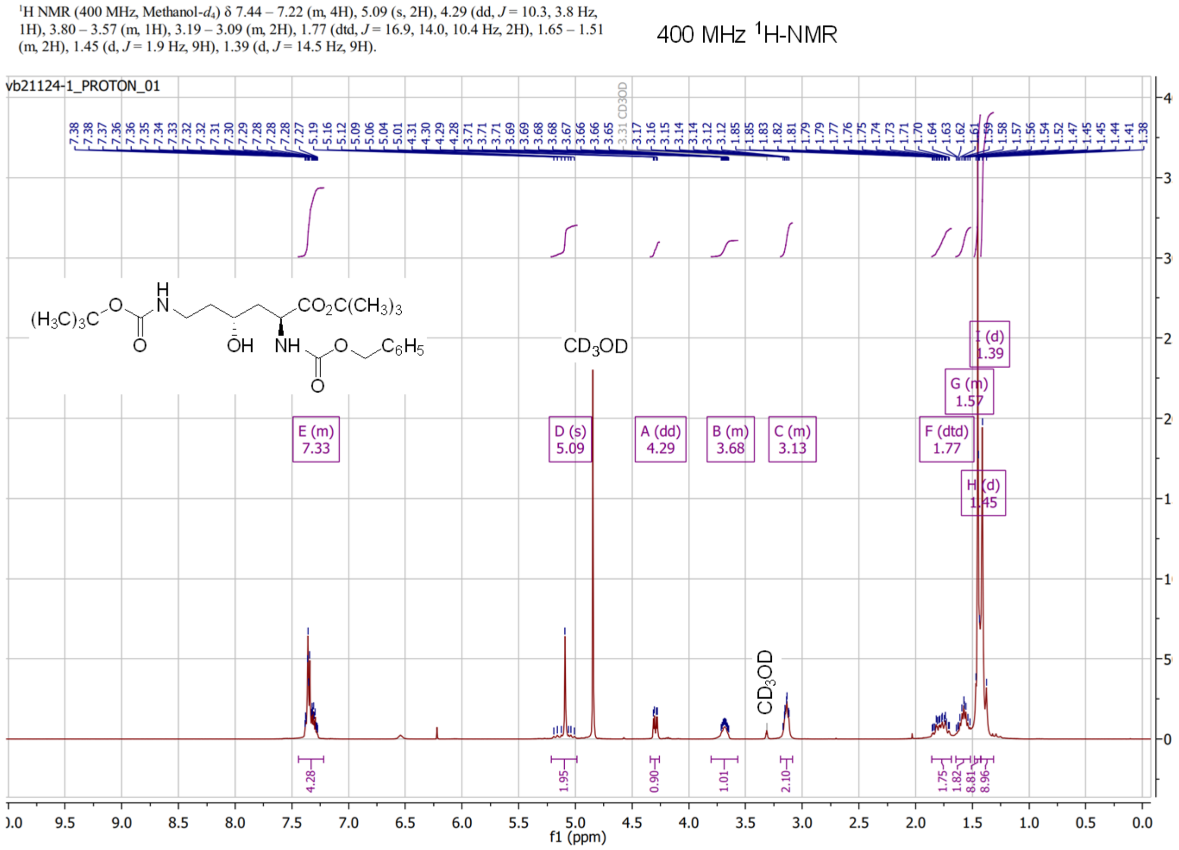

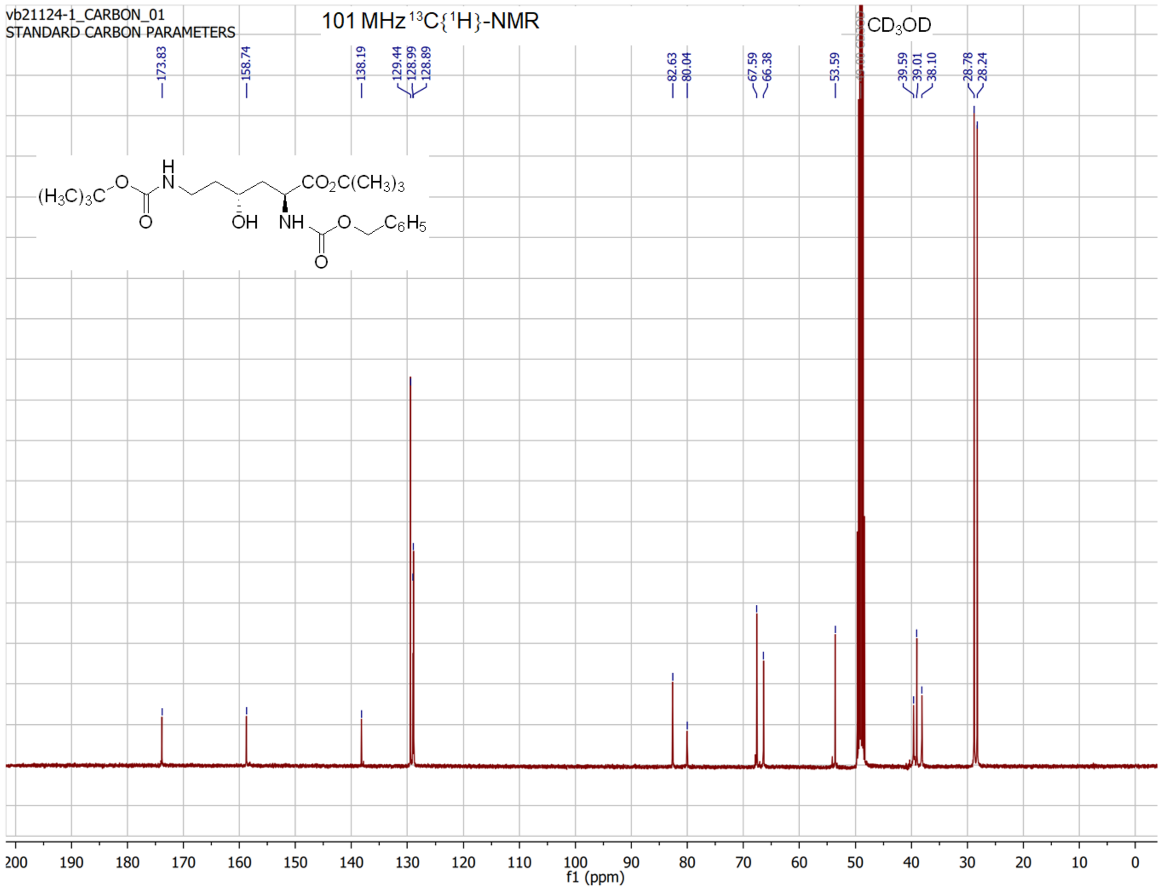

Compound 8 – epimer 2: (2*S*, 4*S*)-BocNH$(CH_2)_2$CH(OH)CH_2CH($NHCO_2CH_2C_6H_5$)CO_2Bu^t

Sample : **<u>vb21124-2</u>**

Gradient: A 20.0 % B 80.0 % ----> A 100.0 % B 0.0 % T = 7 Min.

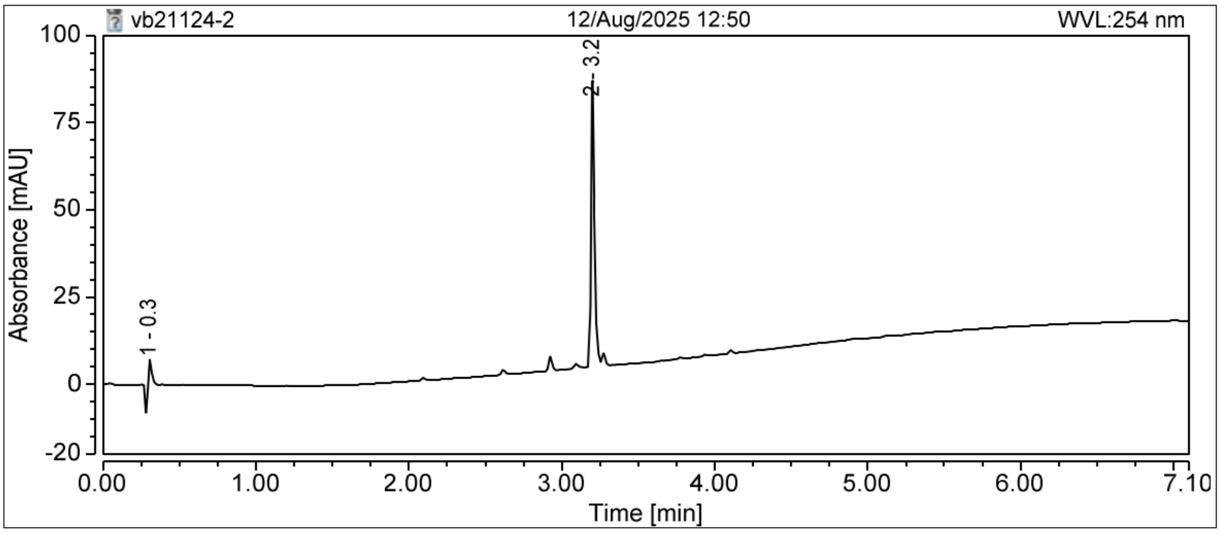

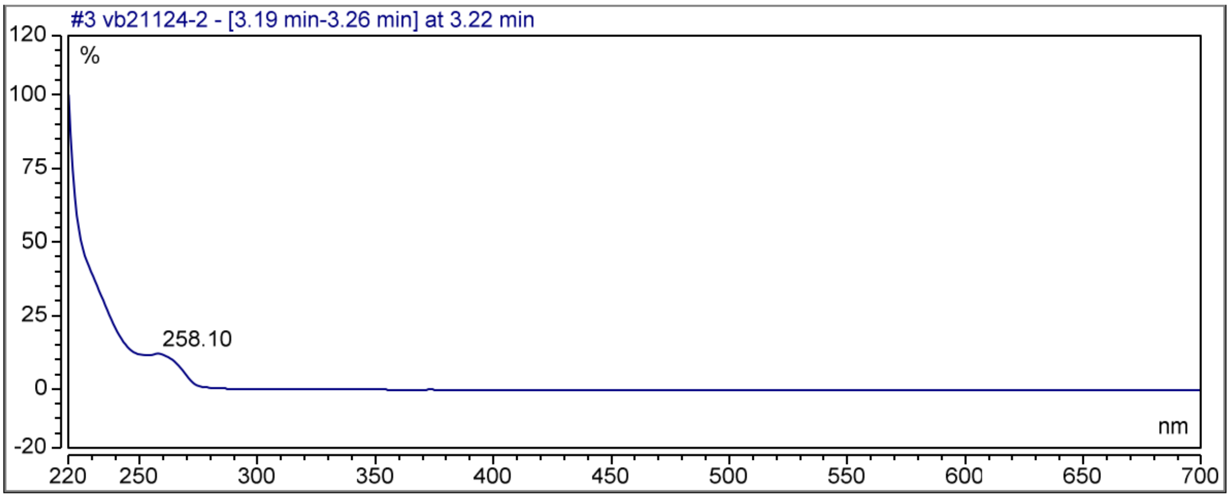

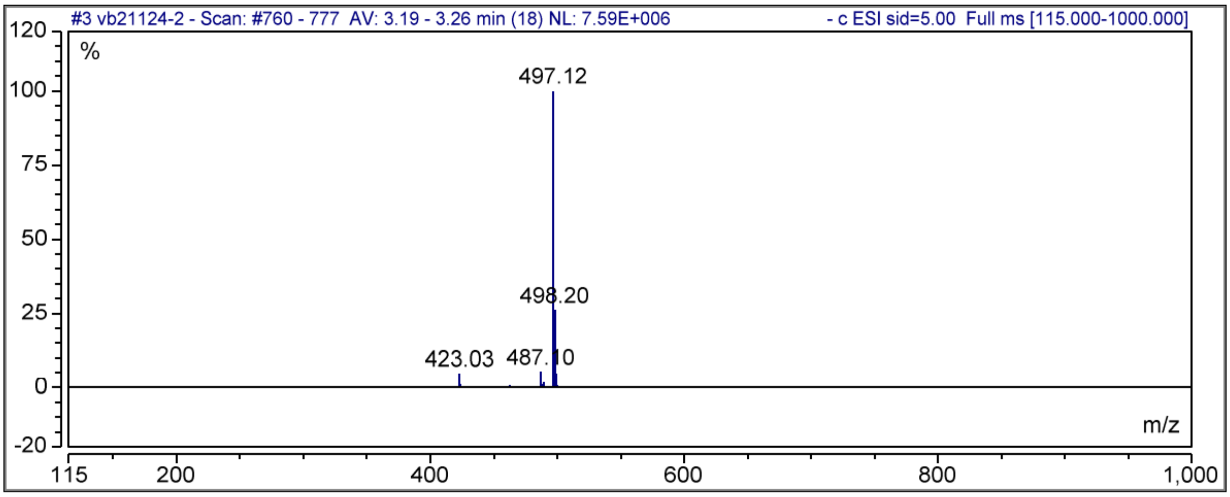

^{1}H-NMR (400 MHz) and ^{13}C{^{1}H}-NMR (101 MHz) spectra in CD_3OD

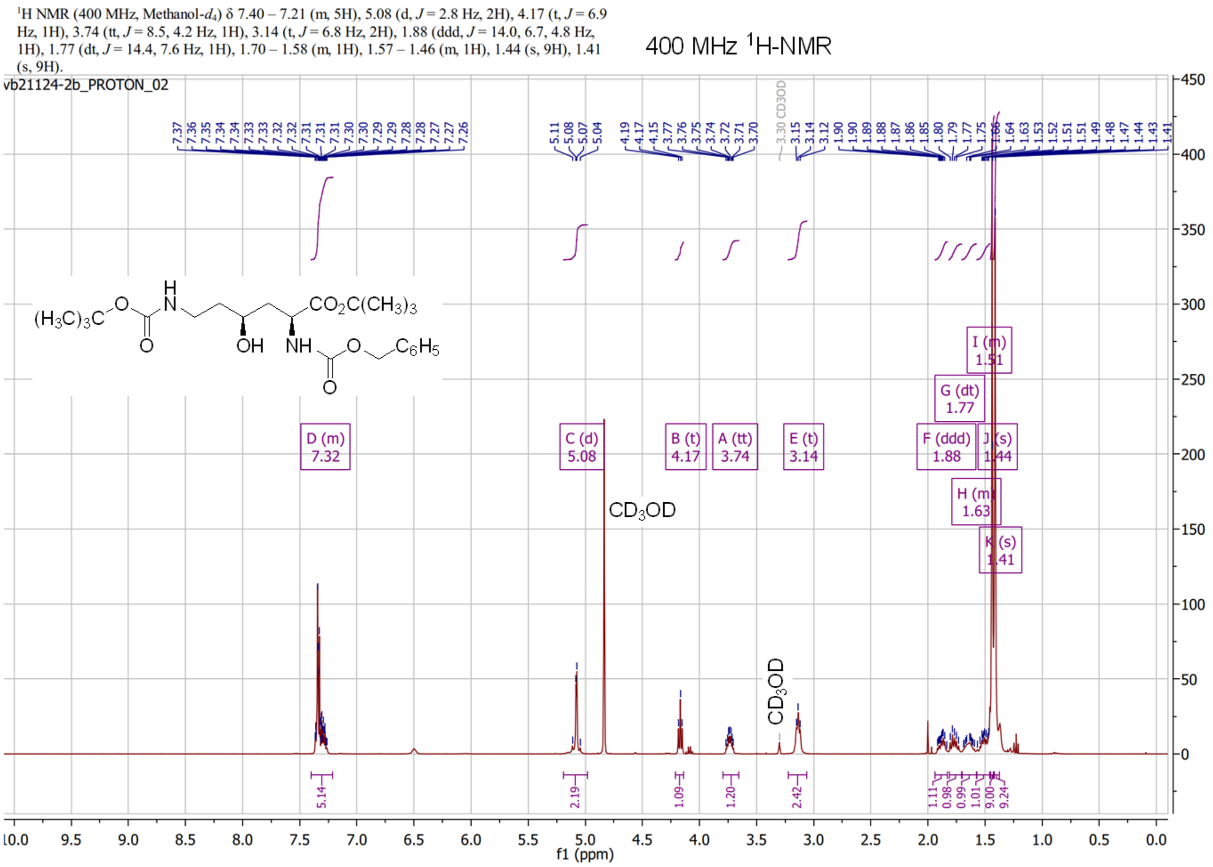

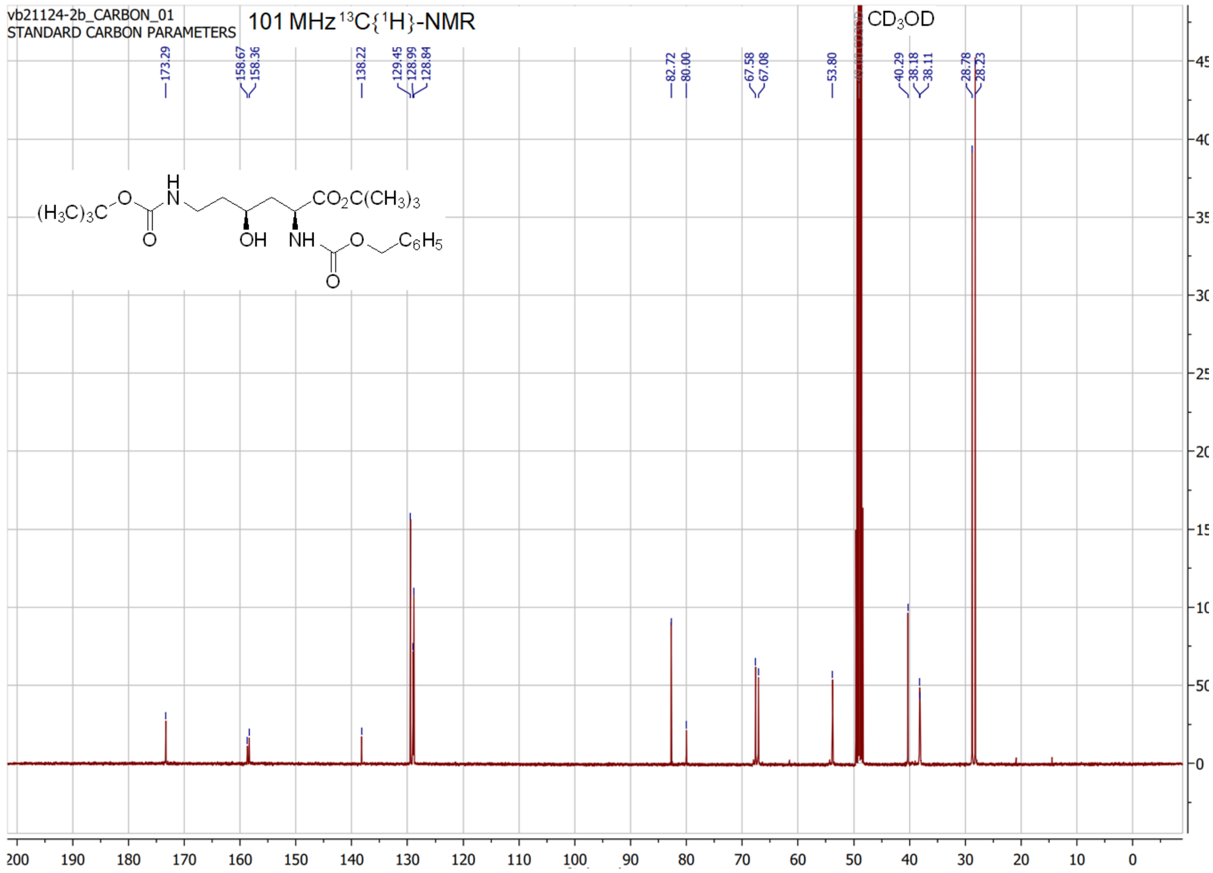

Compound 9 – epimer 1: (2*S*, 4*S*)-BocNH$(CH_2)_2$CHFCH$_2$CH(NHCO$_2$CH$_2$C$_6$H$_5$)CO$_2$Bu^t

Sample : vb21135-1F

Gradient: A 20.0 % B 80.0 % **---->** A 100.0 % B 0.0 % T = 7 Min.

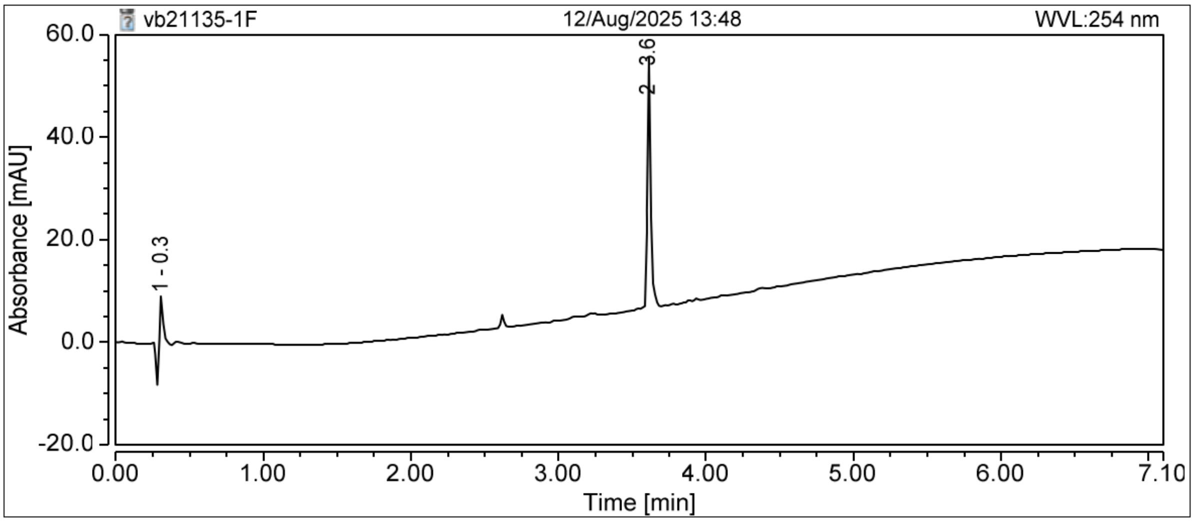

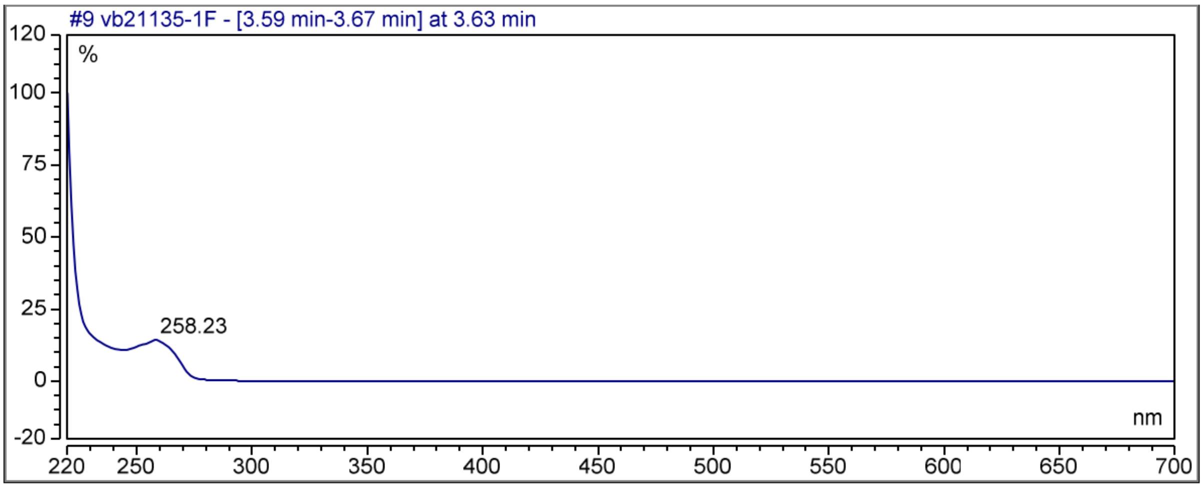

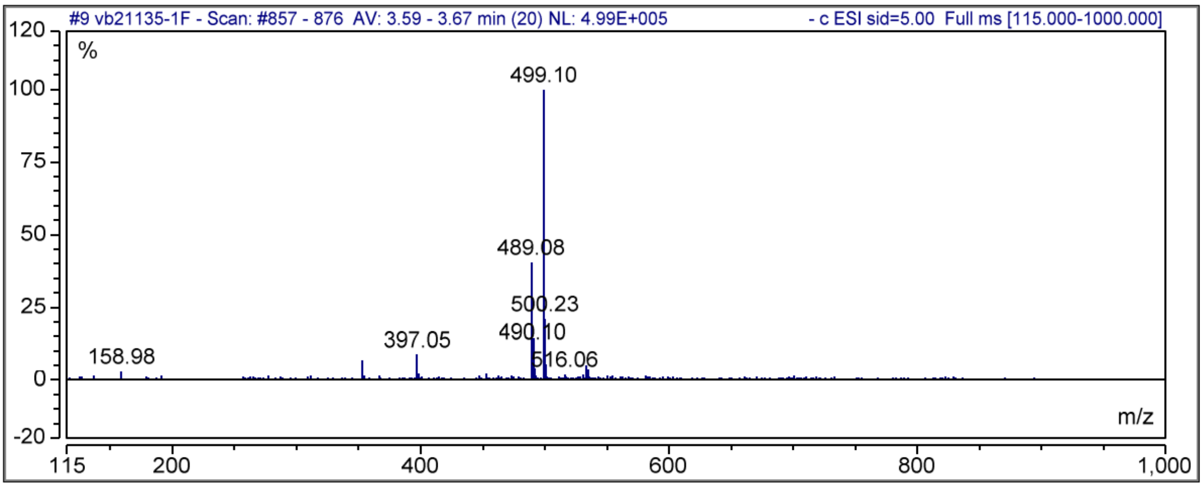

^{1}H-NMR (400 MHz) and ^{13}C{^{1}H}-NMR (101 MHz) spectra in CD_3CN

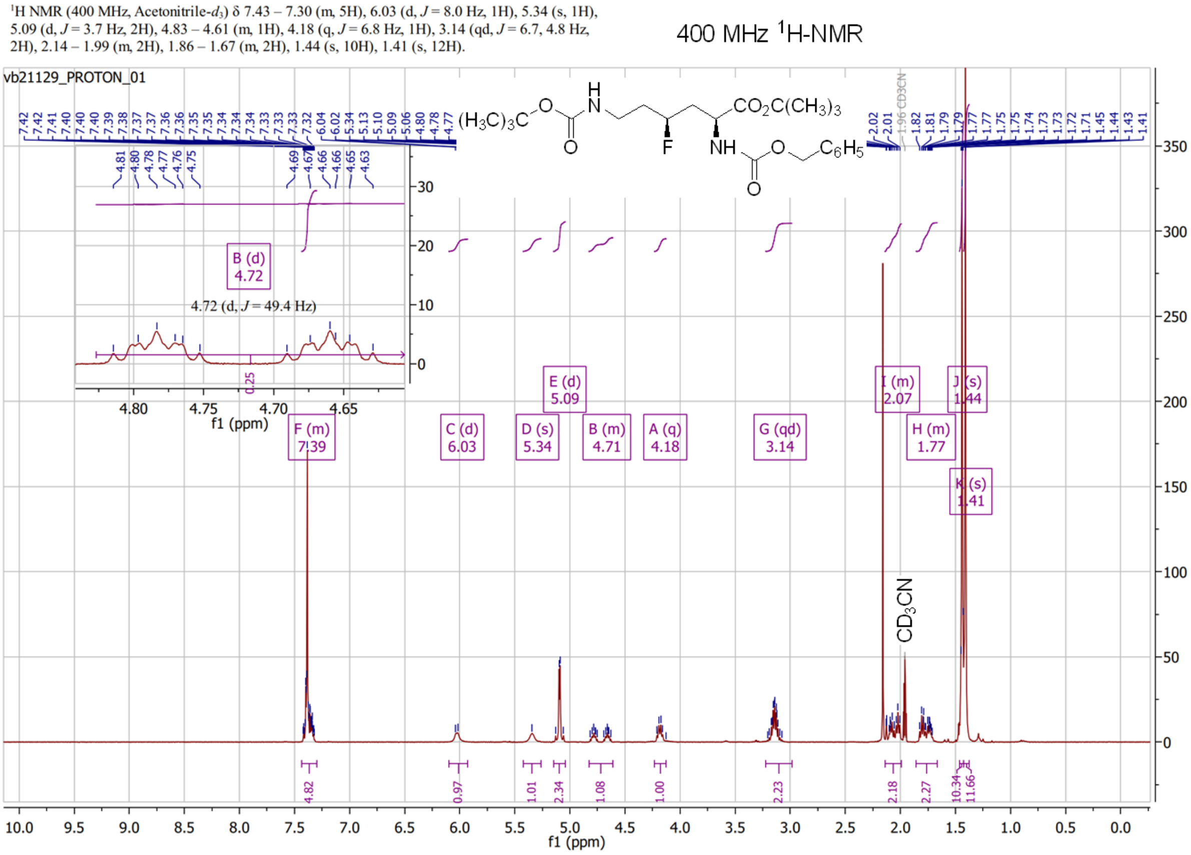

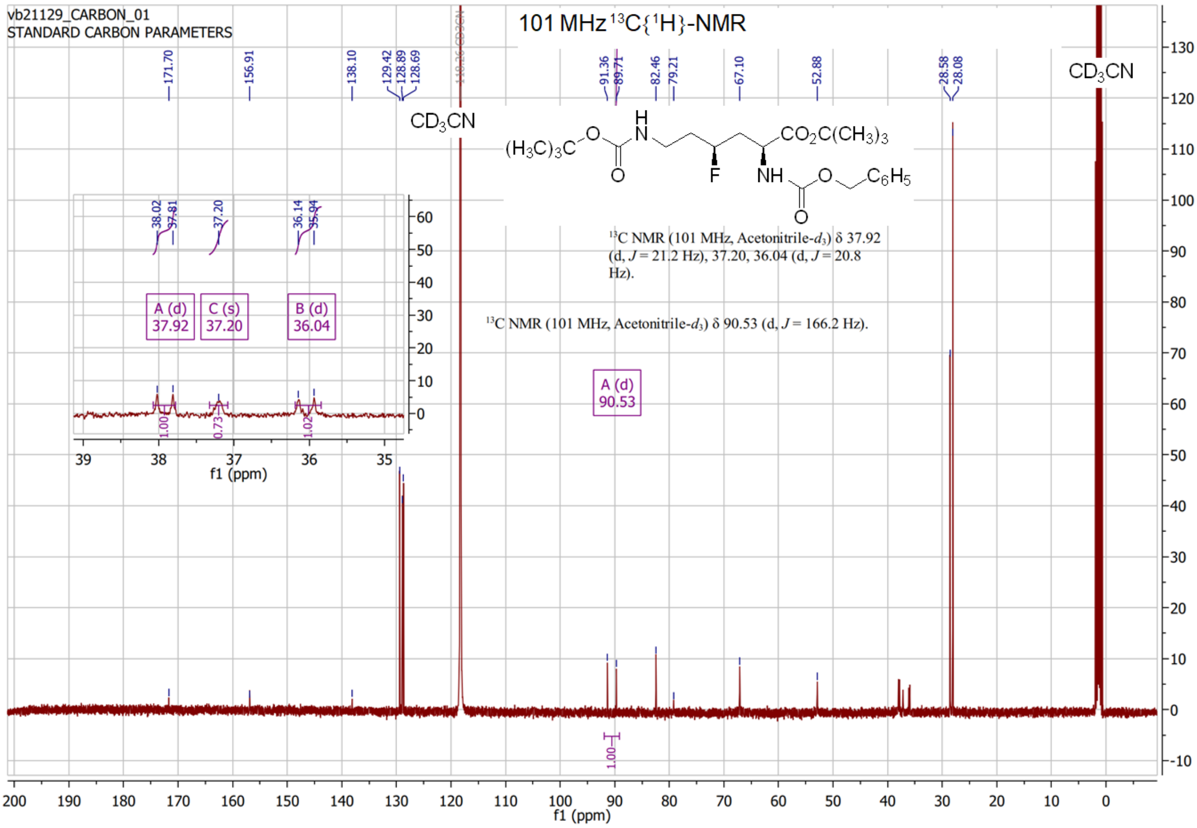

Compound 9 – epimer 2: (2*S*, 4*R*)-$BocNH(CH_2)_2CHFCH_2CH(NHCO_2CH_2C_6H_5)CO_2Bu^t$

Sample : **vb21147-2**

Gradient: A 20.0 % B 80.0 % ----> A 100.0 % B 0.0 % T = 7 Min.

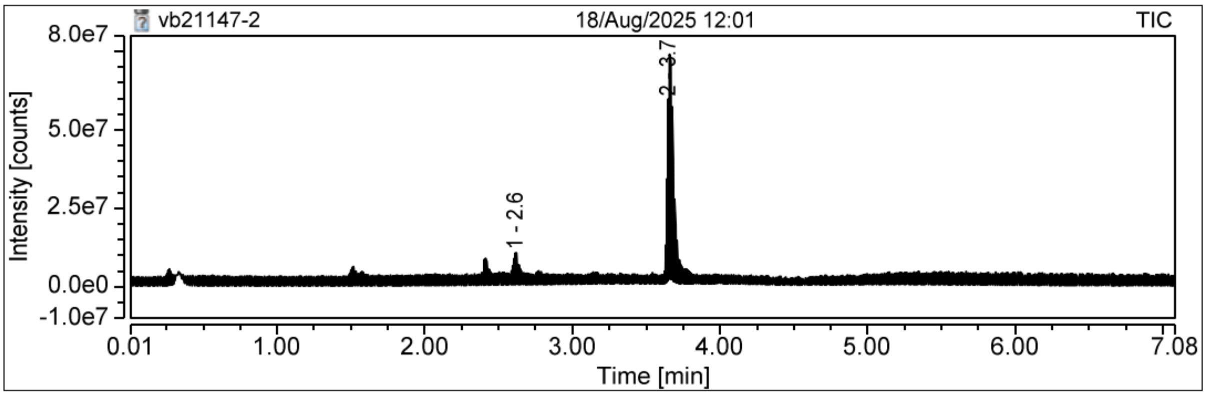

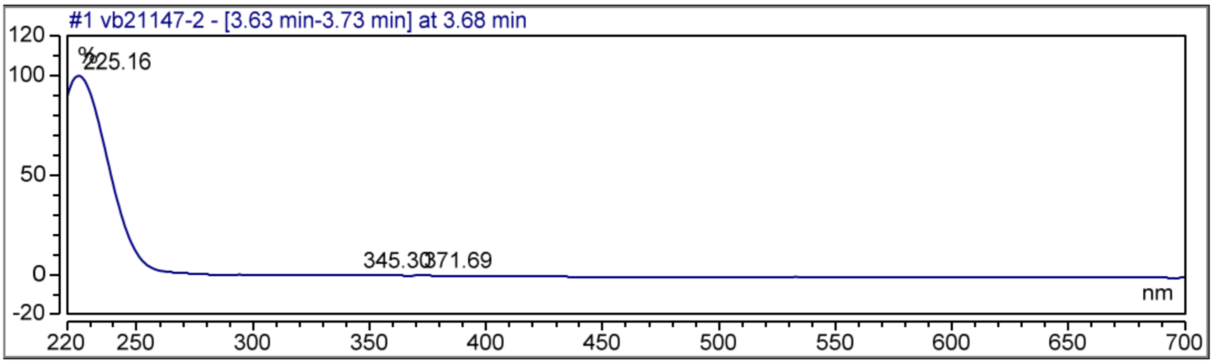

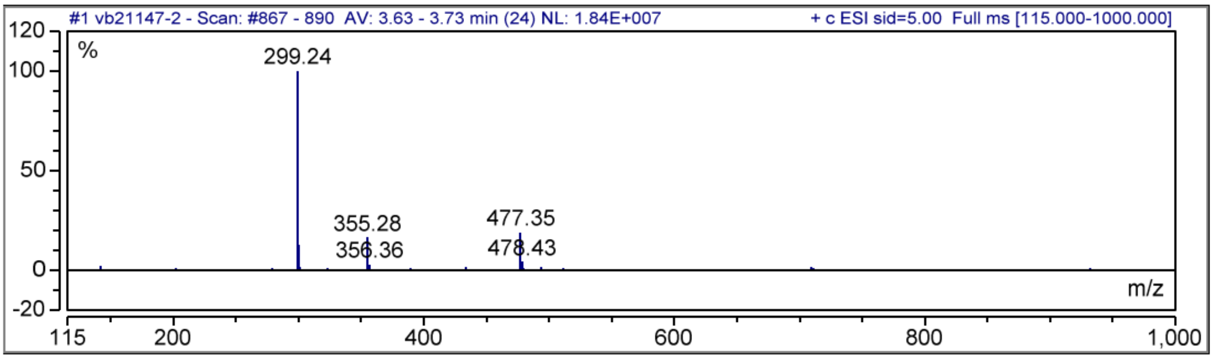

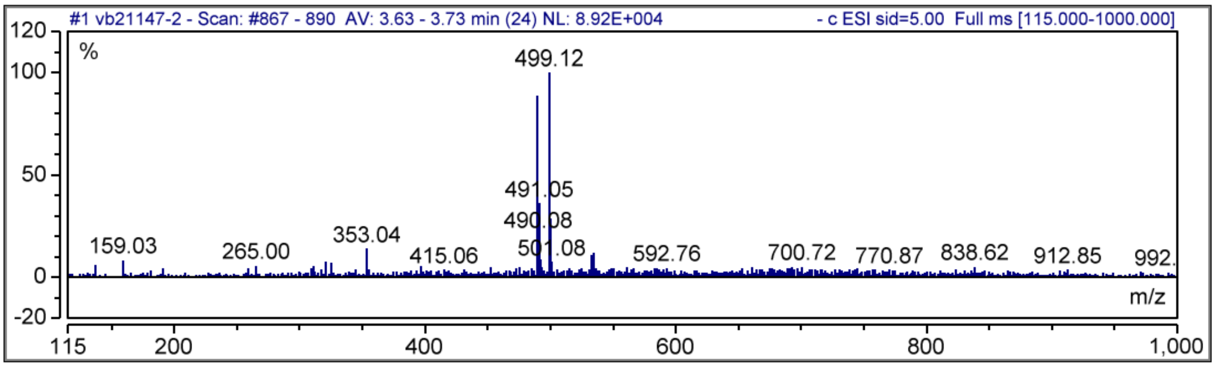

^{1}H-NMR (400 MHz) and ^{13}C{^{1}H}-NMR (101 MHz) spectra in CD_3CN

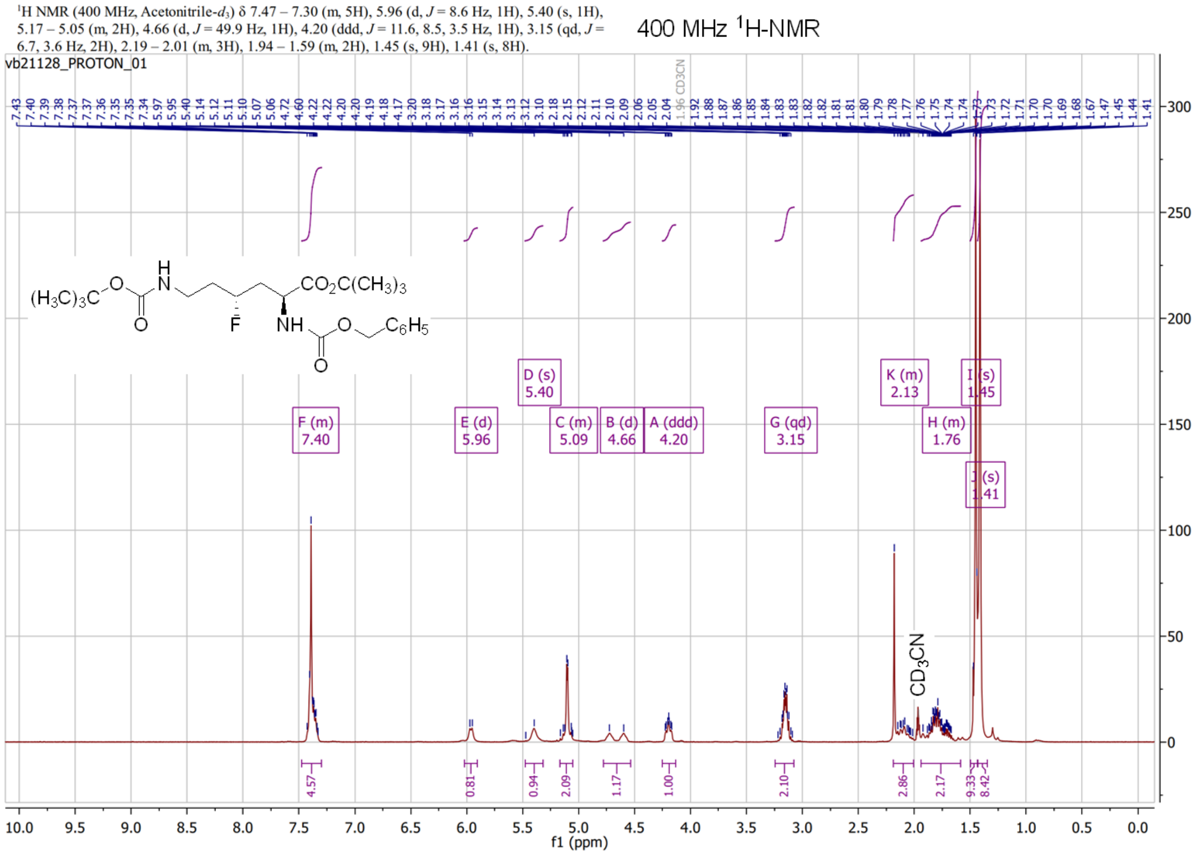

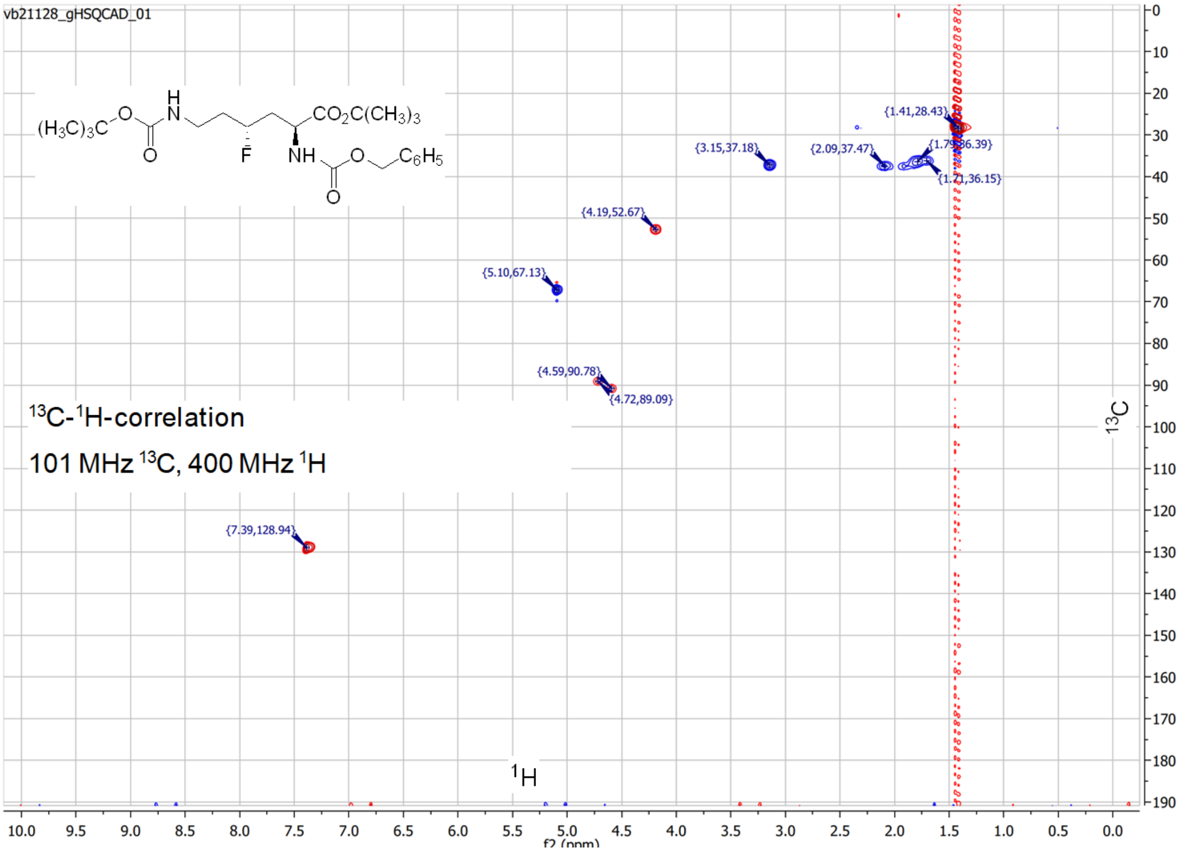

Compound 10 – epimer 1: (2*S*, 4*S*)-$H_2N(CH_2)_2CHFCH_2CH(NHCO_2CH_2C_6H_5)CO_2H*0.17(CH_3CH_2)_3N$

Sample : <u>vb21134-1b</u>

Gradient: A 2.0 % B 98.0 % ----> A 50.0 % B 50.0 % T = 7 Min.

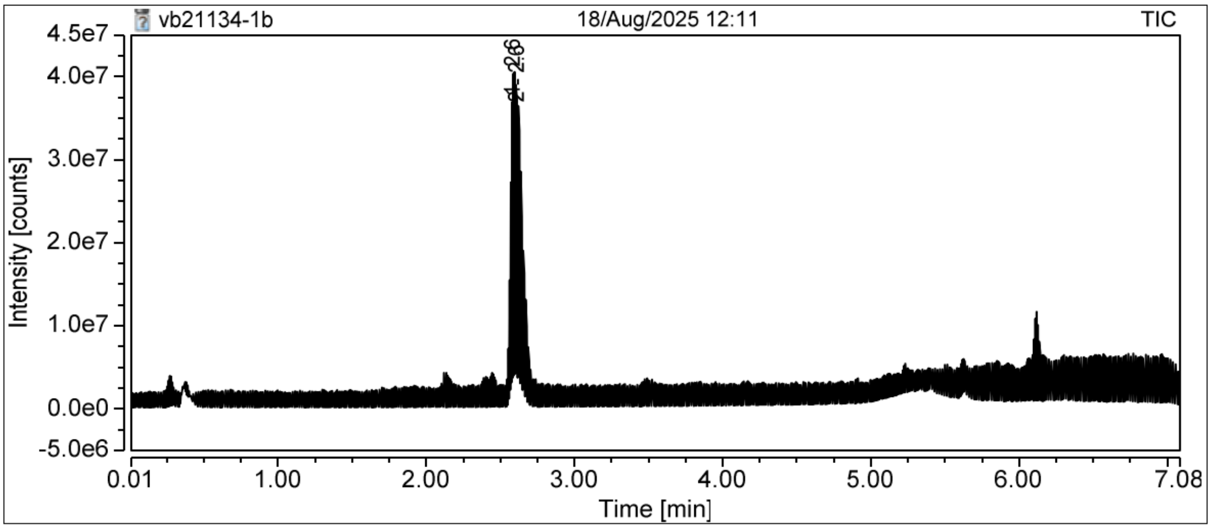

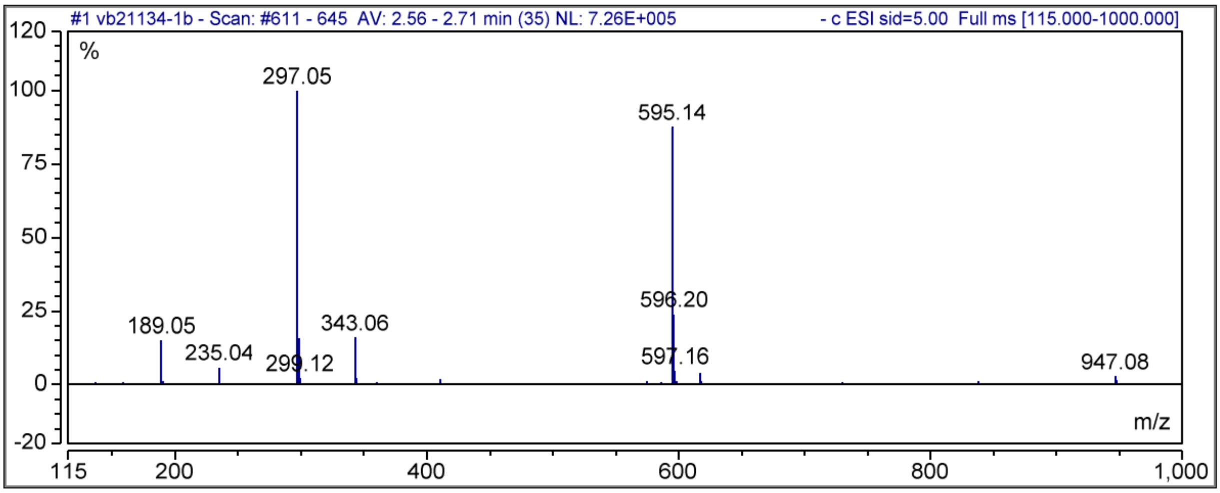

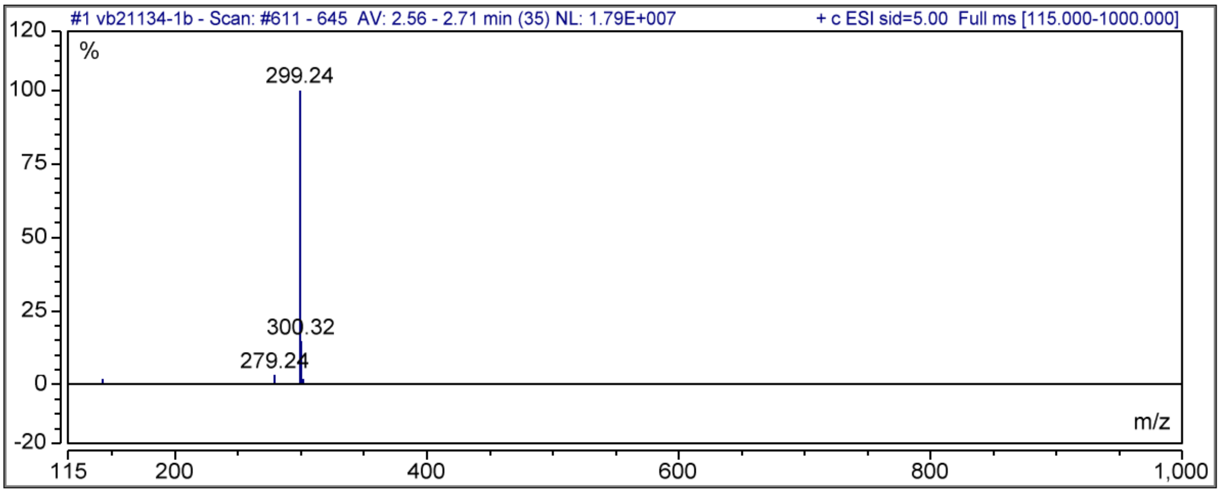

^{1}H-NMR (400 MHz) spectrum in CD_3CN, ^{13}C{^{1}H}-NMR (101 MHz) spectrum in D_2O

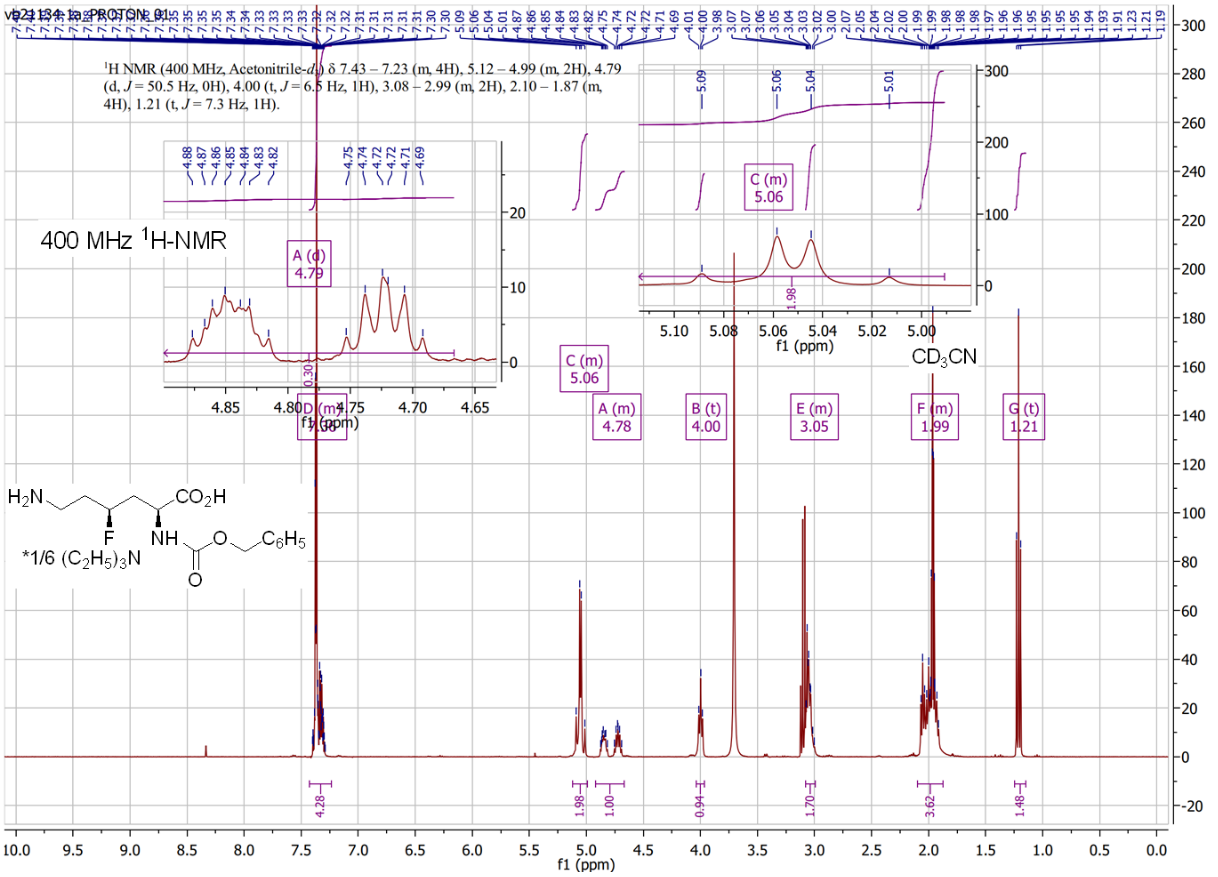

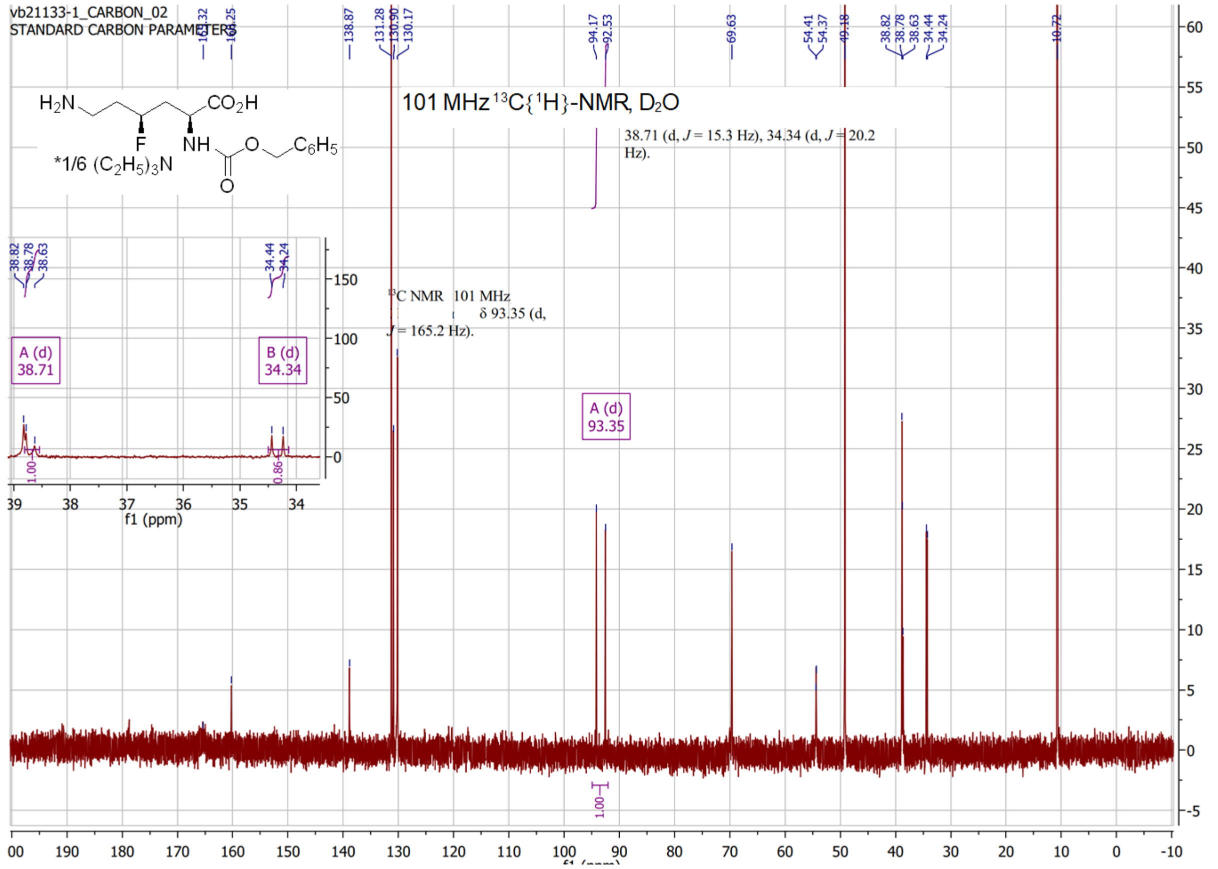

Compound 10 – epimer 2: (2*S*,4*R*)-$H_2N(CH_2)_2CHFCH_2CH(NHCO_2CH_2C_6H_5)CO_2H*0.6(CH_3CH_2)_3N$

Sample : vb21134-2b

Gradient: A 2.0 % B 98.0 % **---->** A 50.0 % B 50.0 % T = 7 Min.

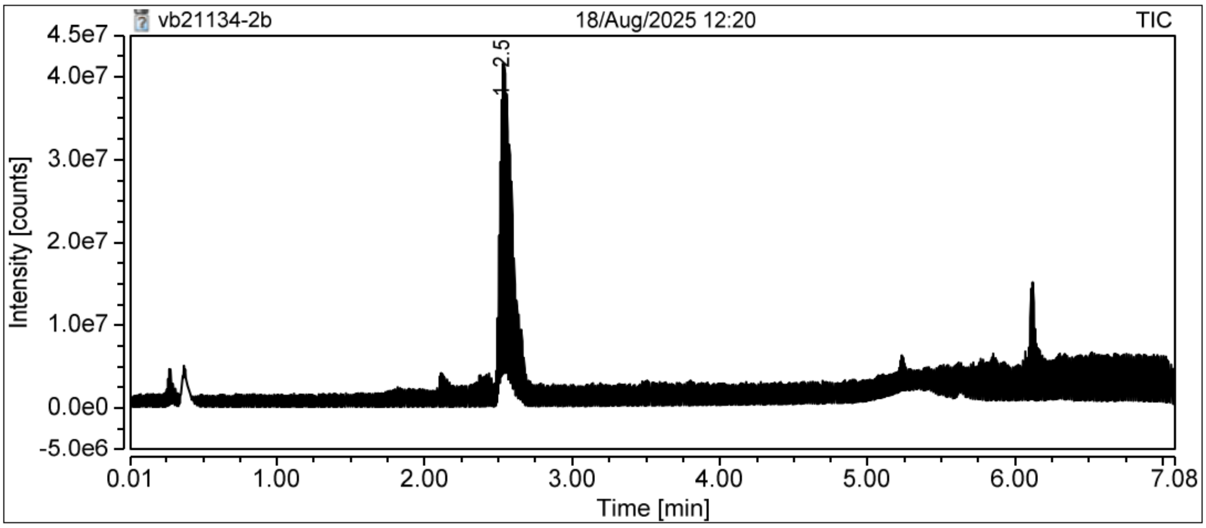

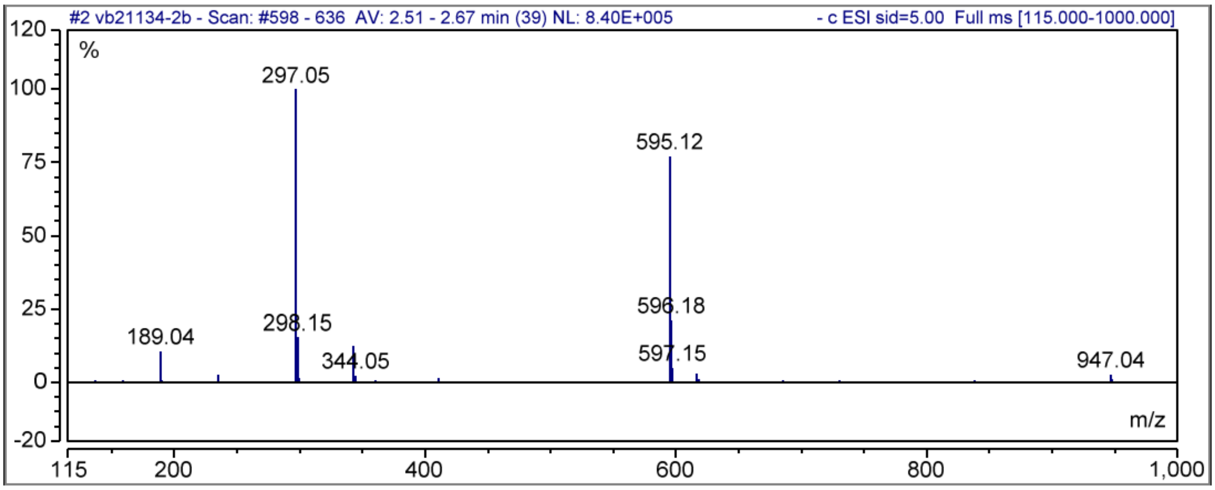

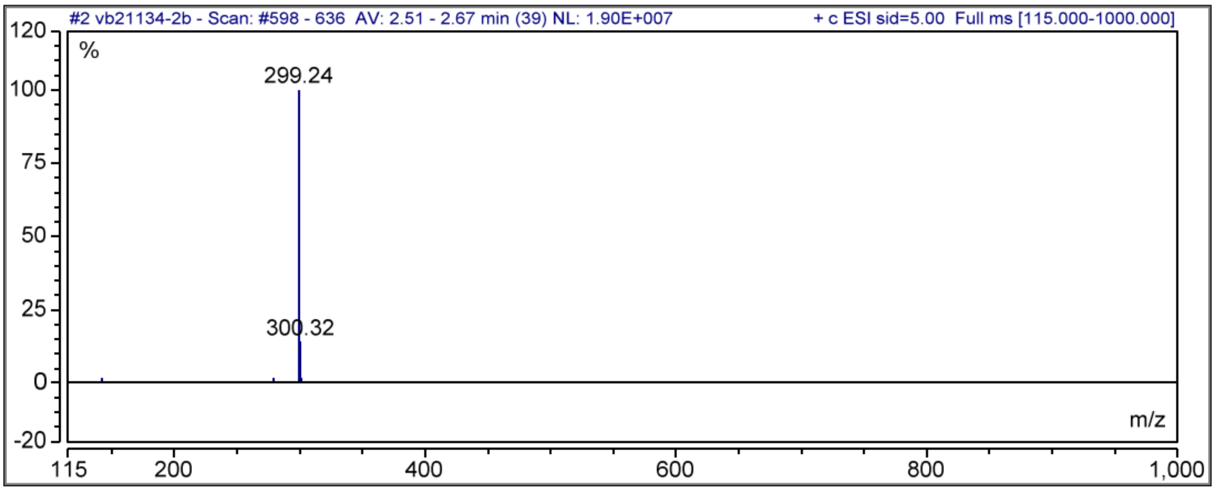

^{1}H-NMR (400 MHz) spectrum in CD_3CN+D_2O, $^{13}C\{^{1}H\}$-NMR (101 MHz) - in D_2O

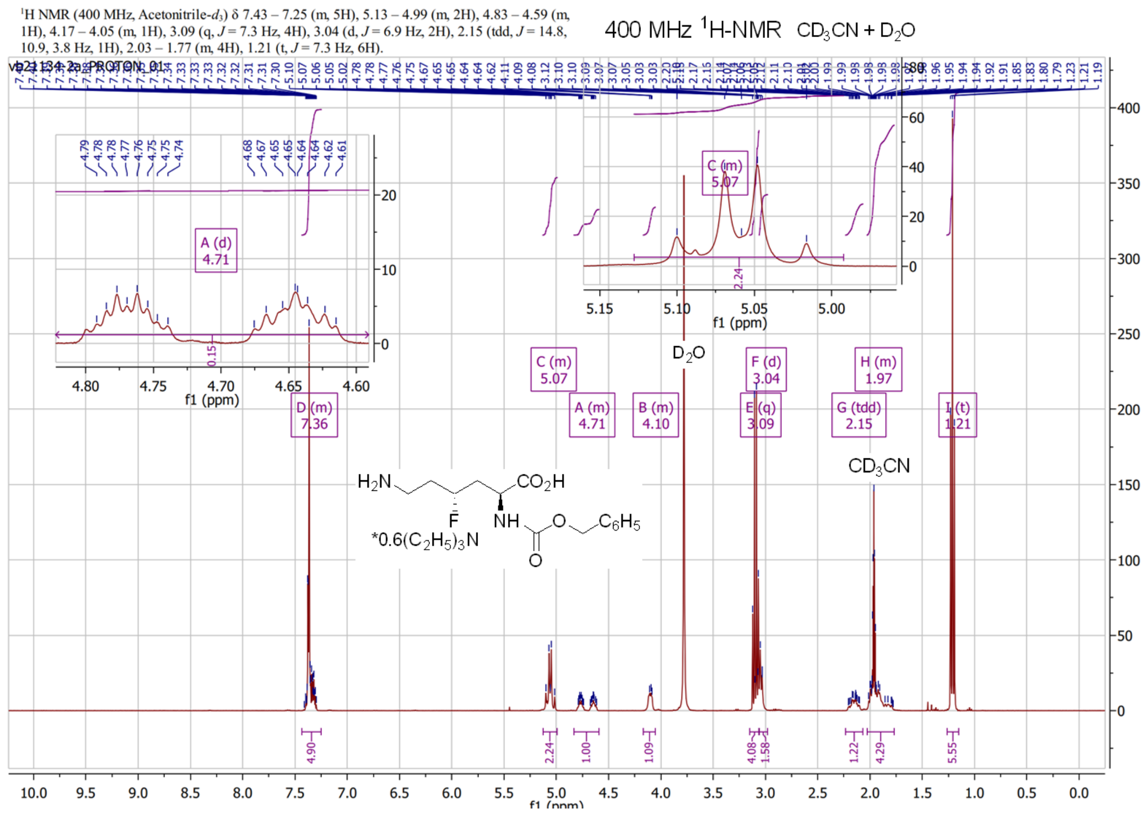

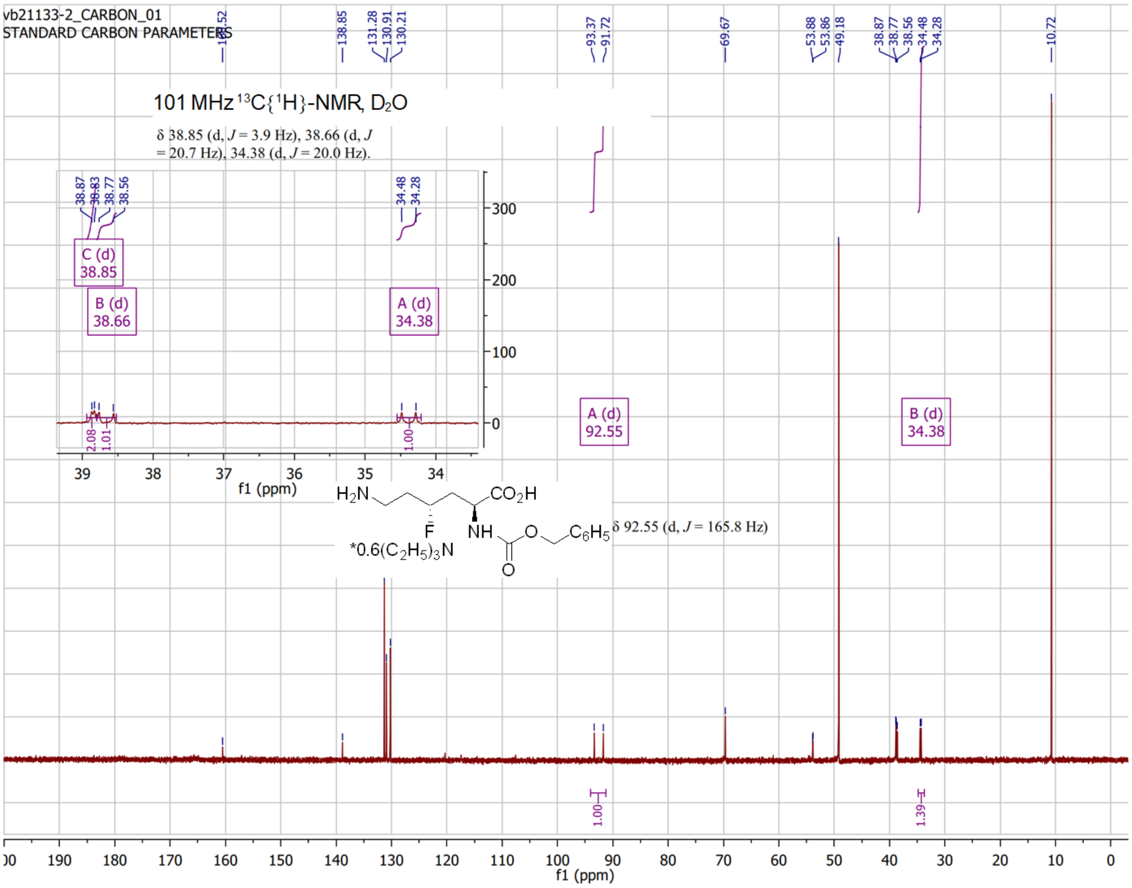

Compound 11 – epimer 1: (2*S*, 4*S*)-$H_2N(CH_2)_2CHFCH_2CH(NH_2)CO_2H$*⅓$CF_3COOH$

Sample : **vb21138-1**

Gradient: A 2.0 % B 98.0 % ----> A 50.0 % B 50.0 % T = 7 Min.

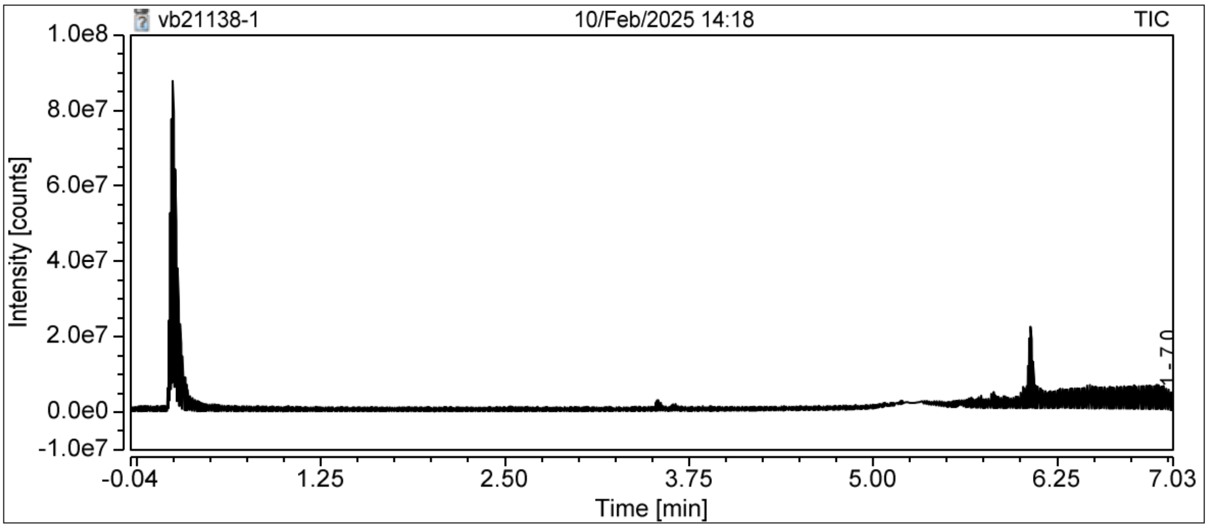

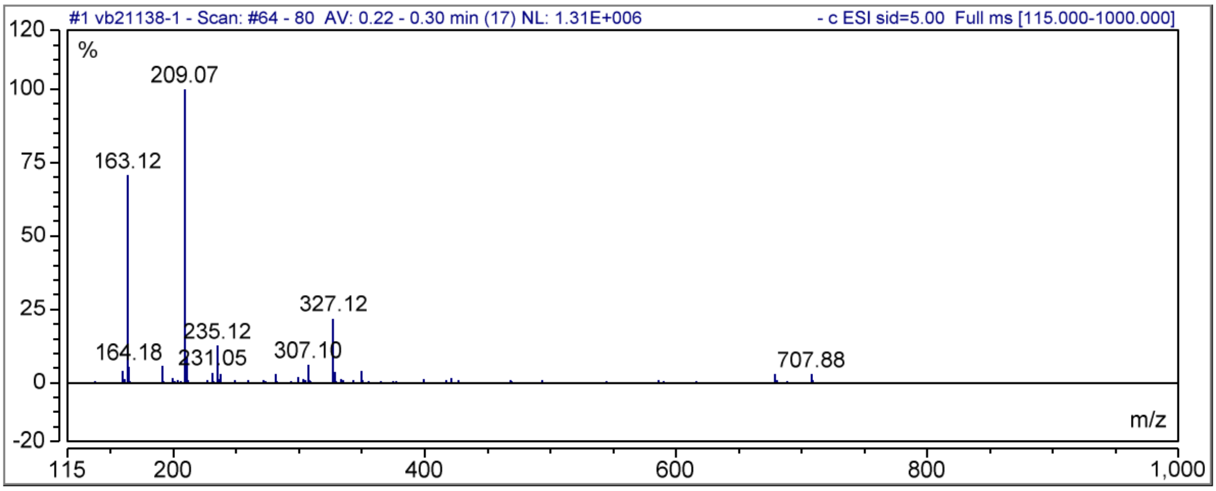

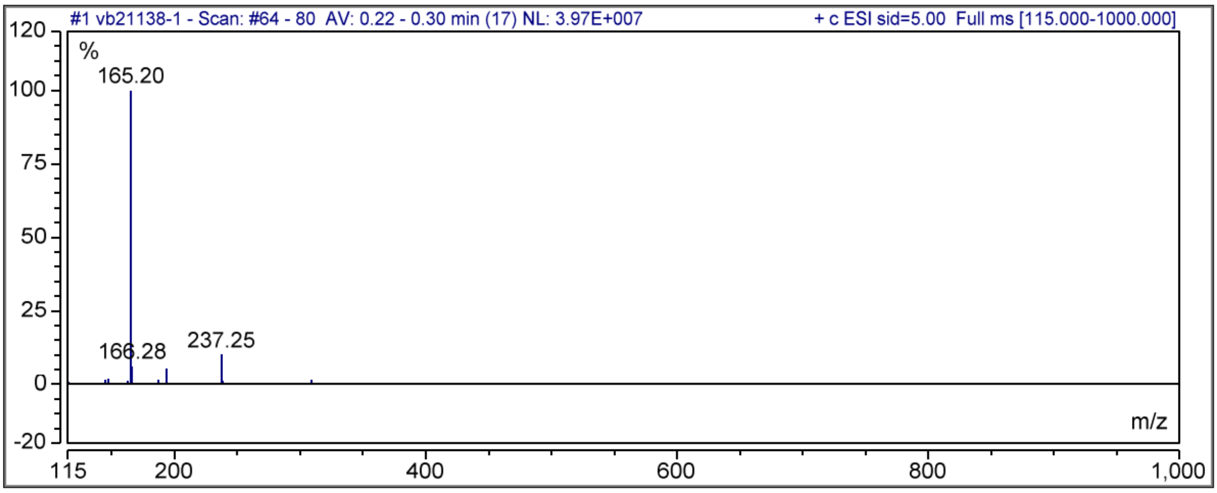

^{1}H-NMR (400 MHz), $^{13}C\{^{1}H\}$-NMR (101 MHz) and ^{19}F-NMR (376 MHz) spectra in D_2O

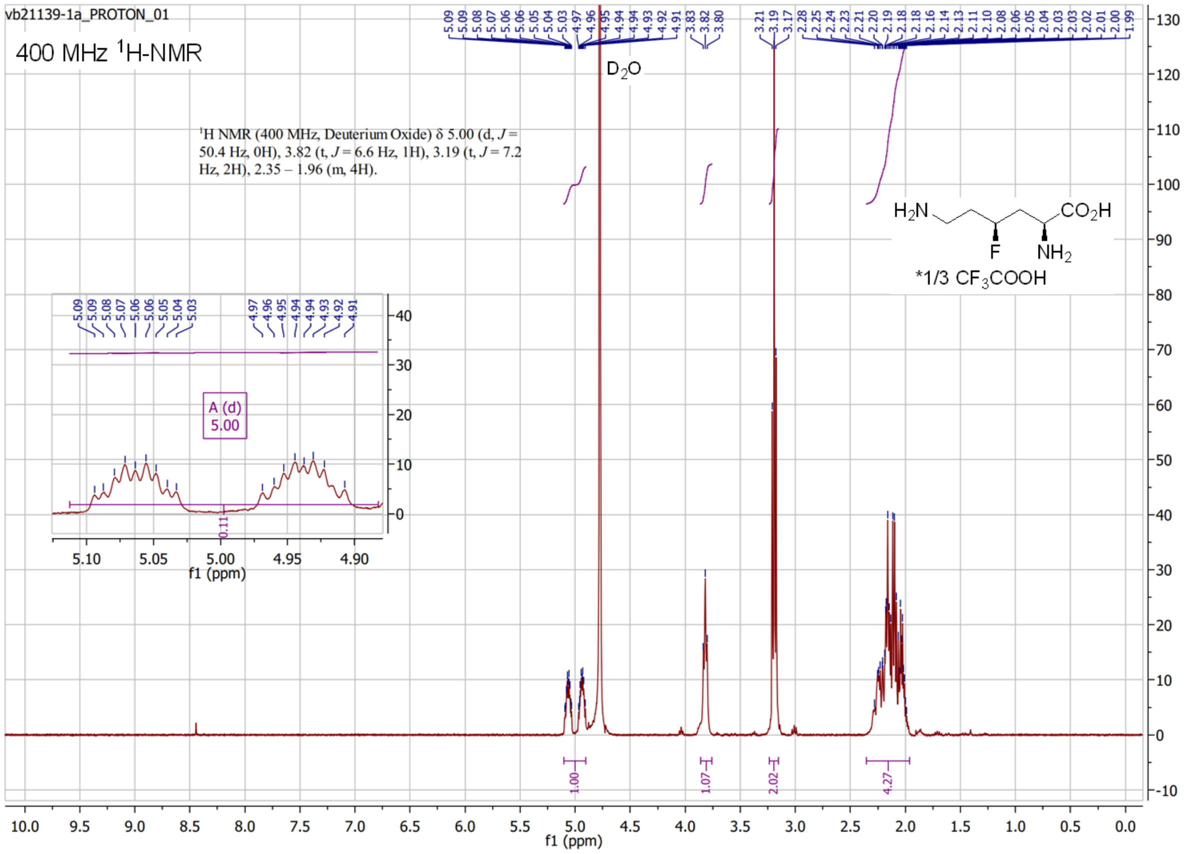

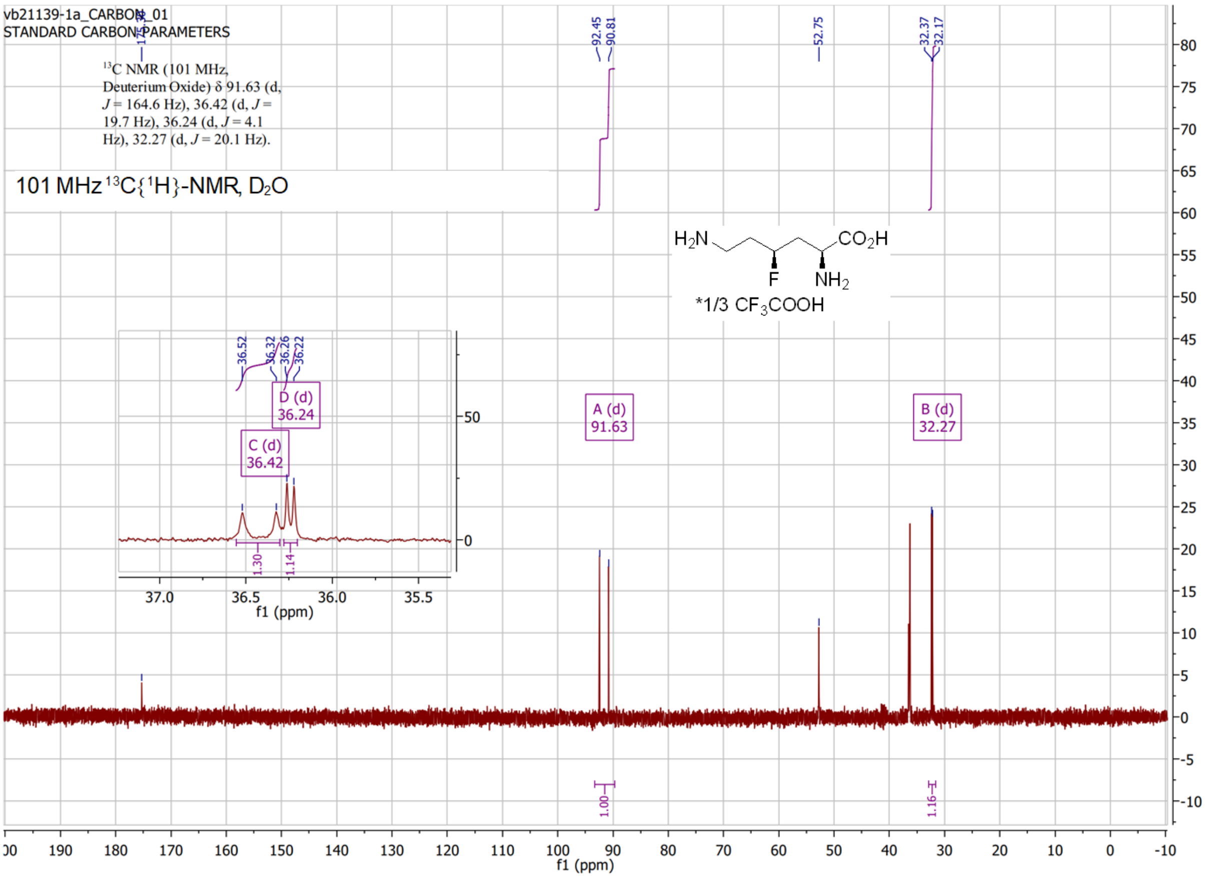

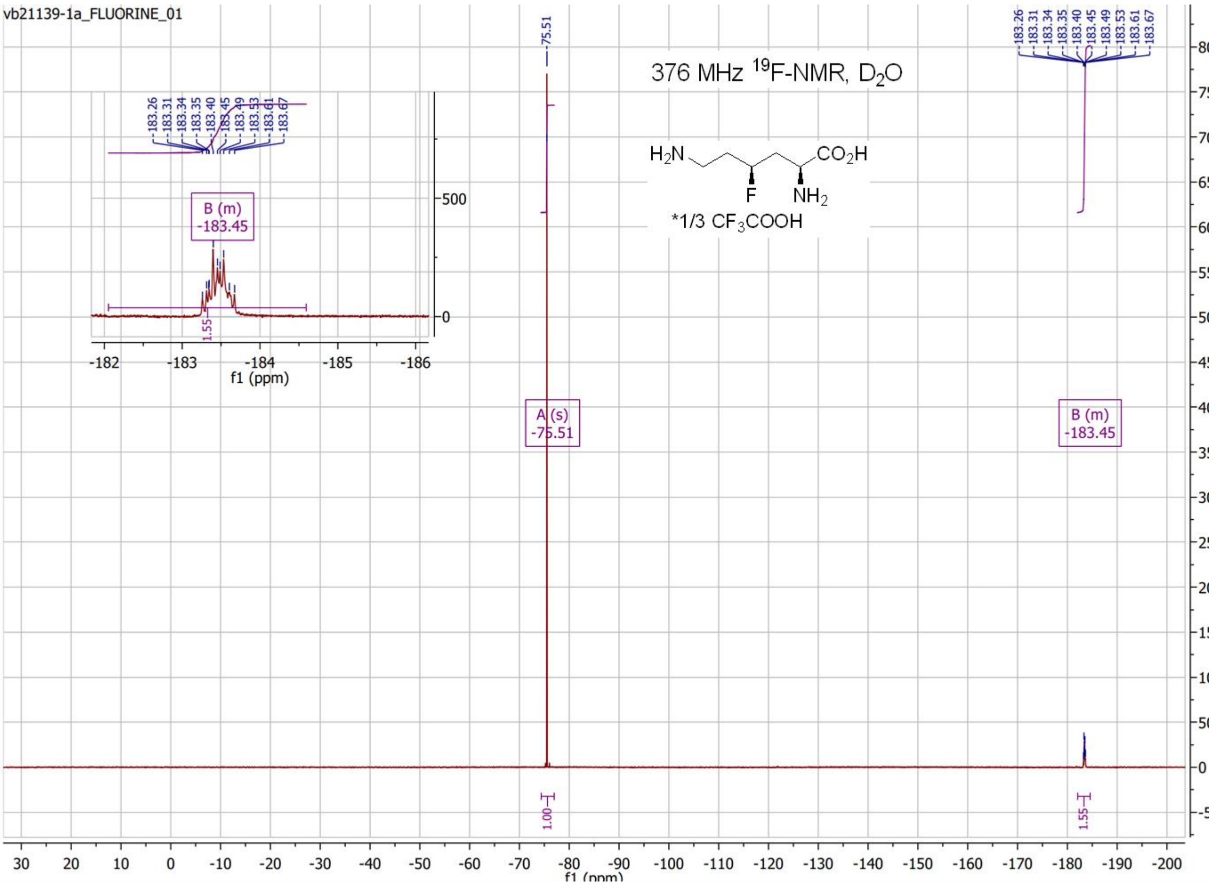
vb21139-1a_FLUORINE_01
376 MHz 19F-NMR, D2O
*1/3 CF3COOH
A (s) -75.51
B (m) -183.45
f1 (ppm)

Compound 11 – epimer 2: (2*S*, 4*R*)-$H_2N(CH_2)_2CHFCH_2CH(NH_2)CO_2H$*0.9$CF_3COOH$*0.1$(C_2H_5)_3N$

Sample : **vb21138-2**

Gradient: A 2.0 % B 98.0 % ----> A 50.0 % B 50.0 % T = 7 Min.

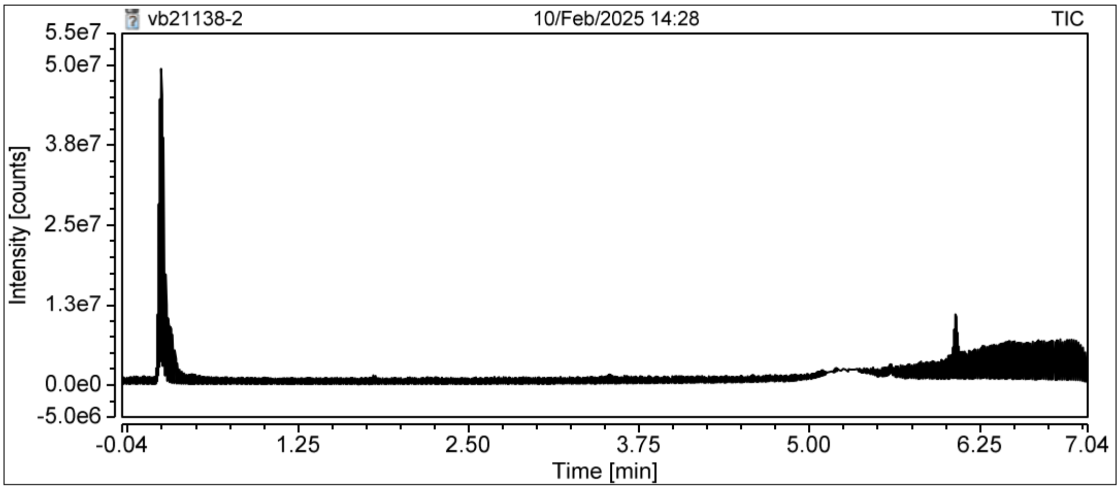

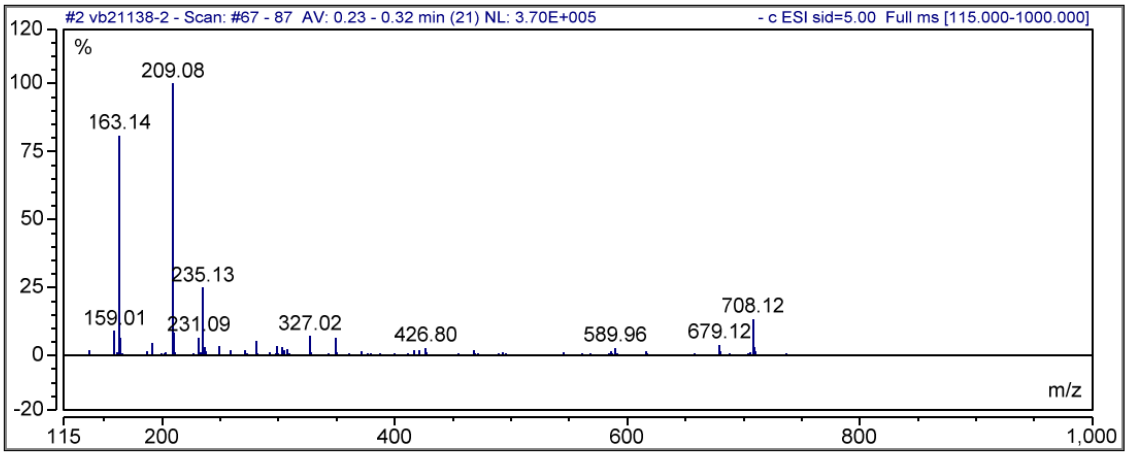

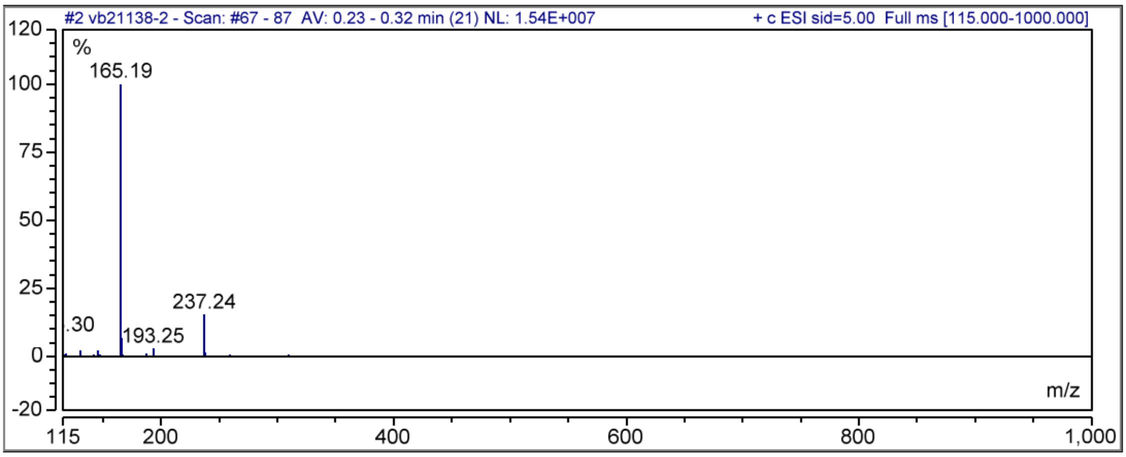

^{1}H-NMR (400 MHz) spectrum in D_2O, $^{13}C\{^{1}H\}$- (101 MHz) and ^{19}F-NMR spectra in CD_3CN

^{1}H NMR (400 MHz, Deuterium Oxide) δ 4.91 (dddd, *J* = 50.8, 12.4, 6.2, 3.1 Hz, 1H), 3.97 (dd, *J* = 7.1, 3.9 Hz, 1H), 3.21 (d, *J* = 7.2 Hz, 2H), 2.41 – 2.20 (m, 2H), 2.20 – 1.97 (m, 2H), 1.28 (t, *J* = 7.3 Hz, 1H).

400 MHz ^{1}H-NMR

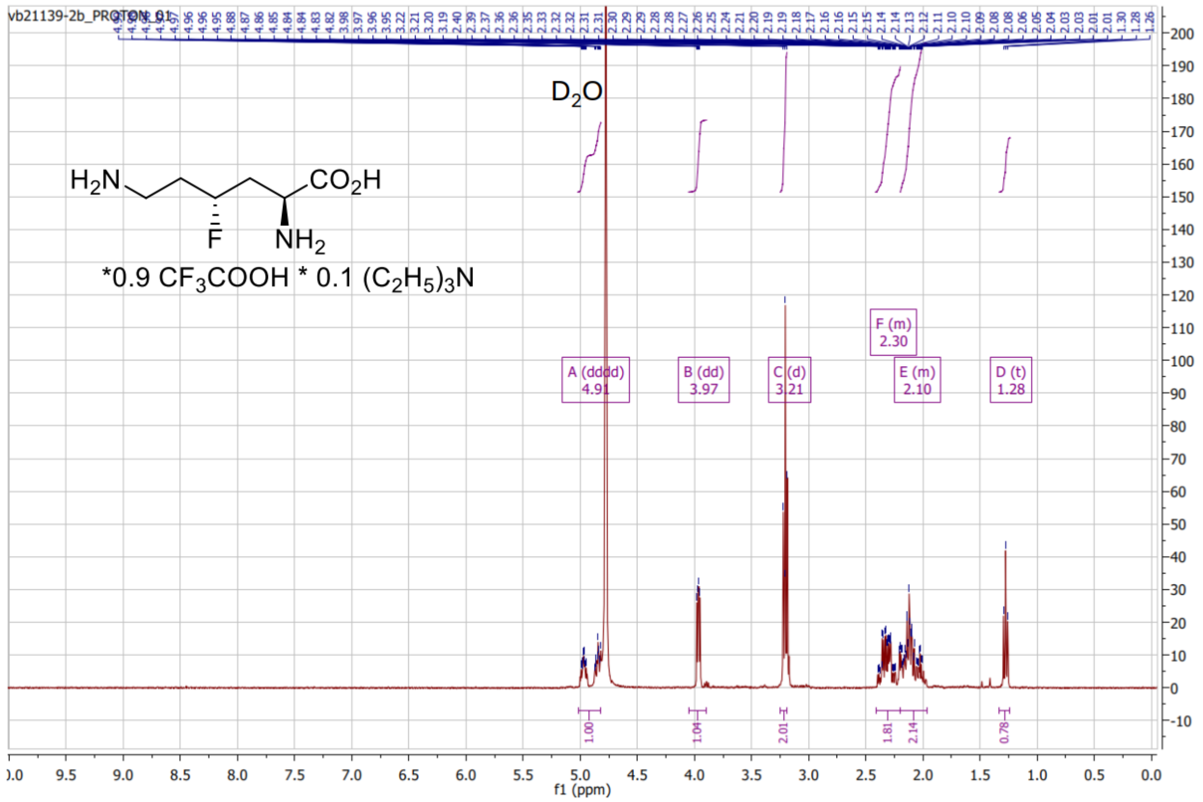

^{13}C NMR (101 MHz, Acetonitrile-d_3) δ 90.36 (d, *J* = 164.2 Hz), 52.00 (d, *J* = 2.6 Hz), 36.90 (d, *J* = 19.8 Hz), 32.42 (d, *J* = 20.2 Hz).

101 MHz $^{13}C\{^{1}H\}$-NMR, CD_3CN

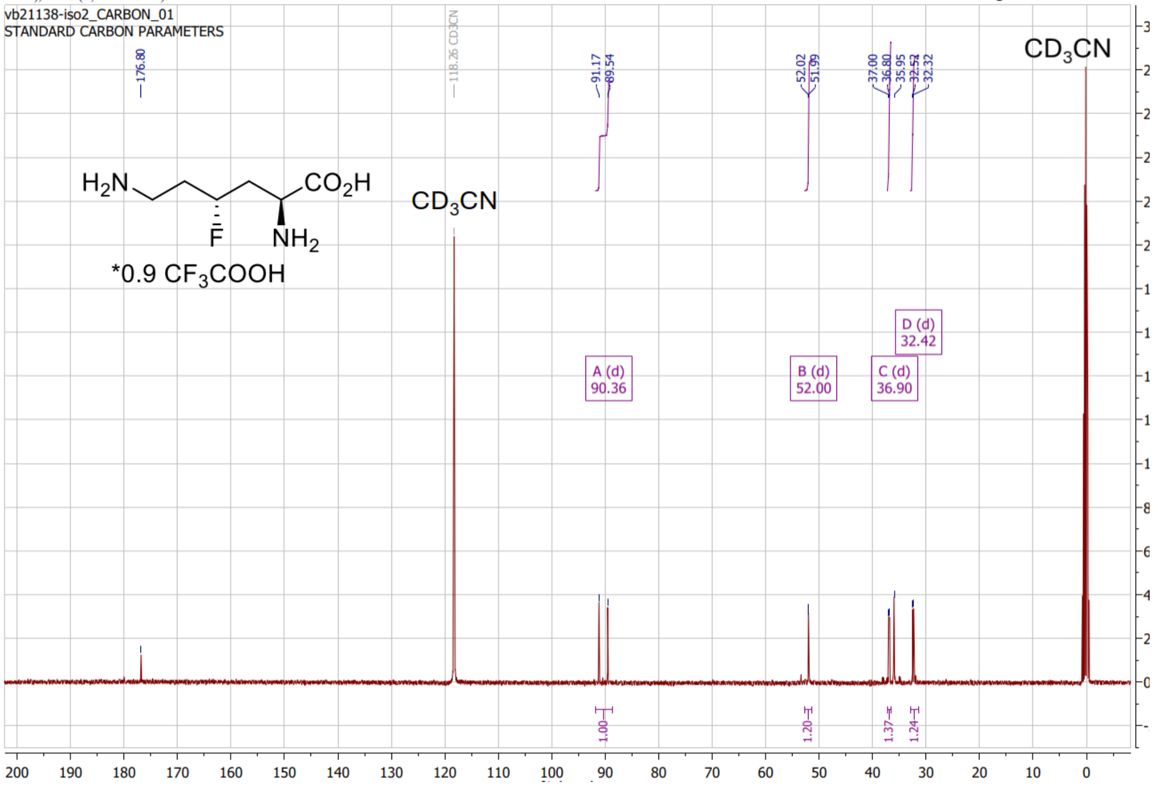

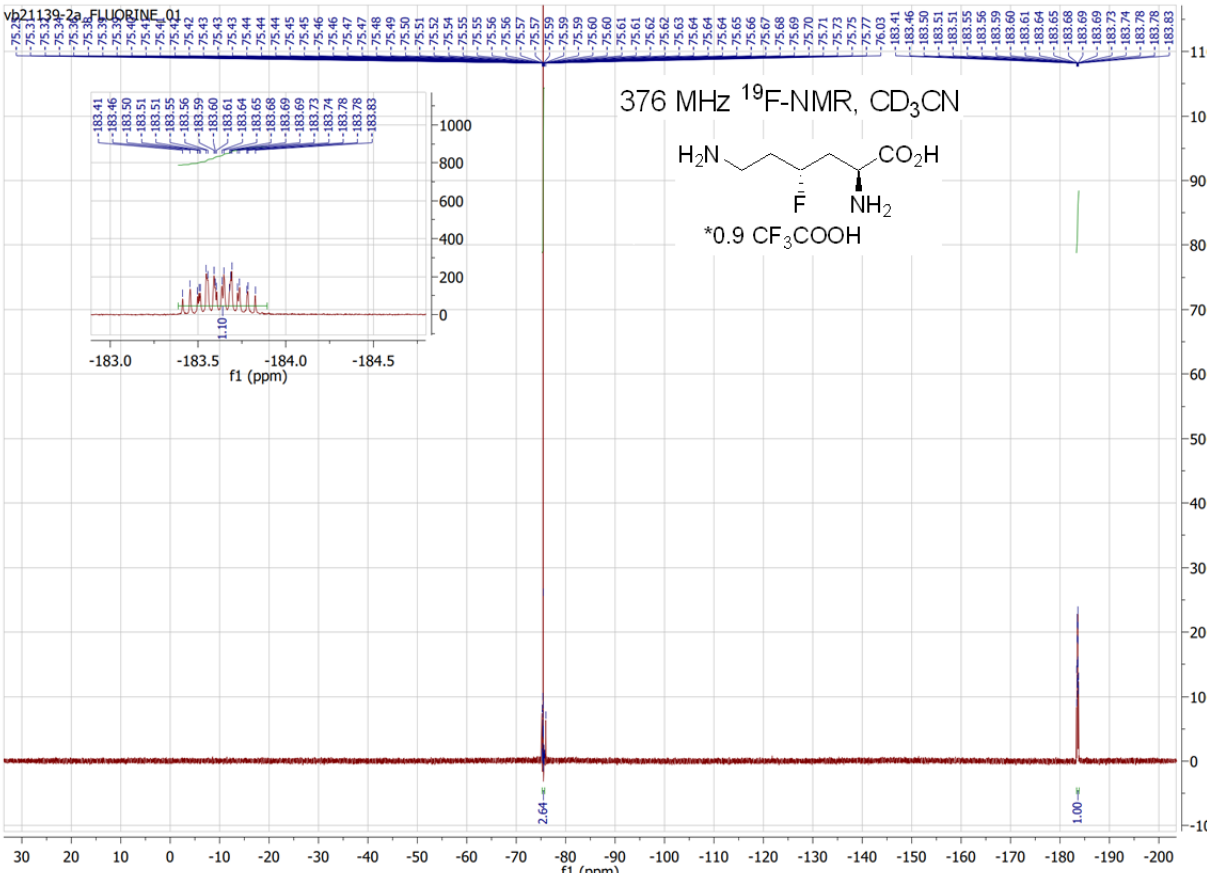

vb21139-2a_FLUORINE_01
376 MHz ^{19}F-NMR, CD_3CN
*0.9 CF_3COOH
f1 (ppm)

Compound 12 - epimer 1, (2*S*,4*S*)-BocNH$(CH_2)_2$CHFCH$_2$CH(NH_2)CO$_2$Bu^t, ^{1}H-NMR (400 MHz) and ^{13}C{^{1}H}-NMR (101 MHz) spectra in CD$_3$CN

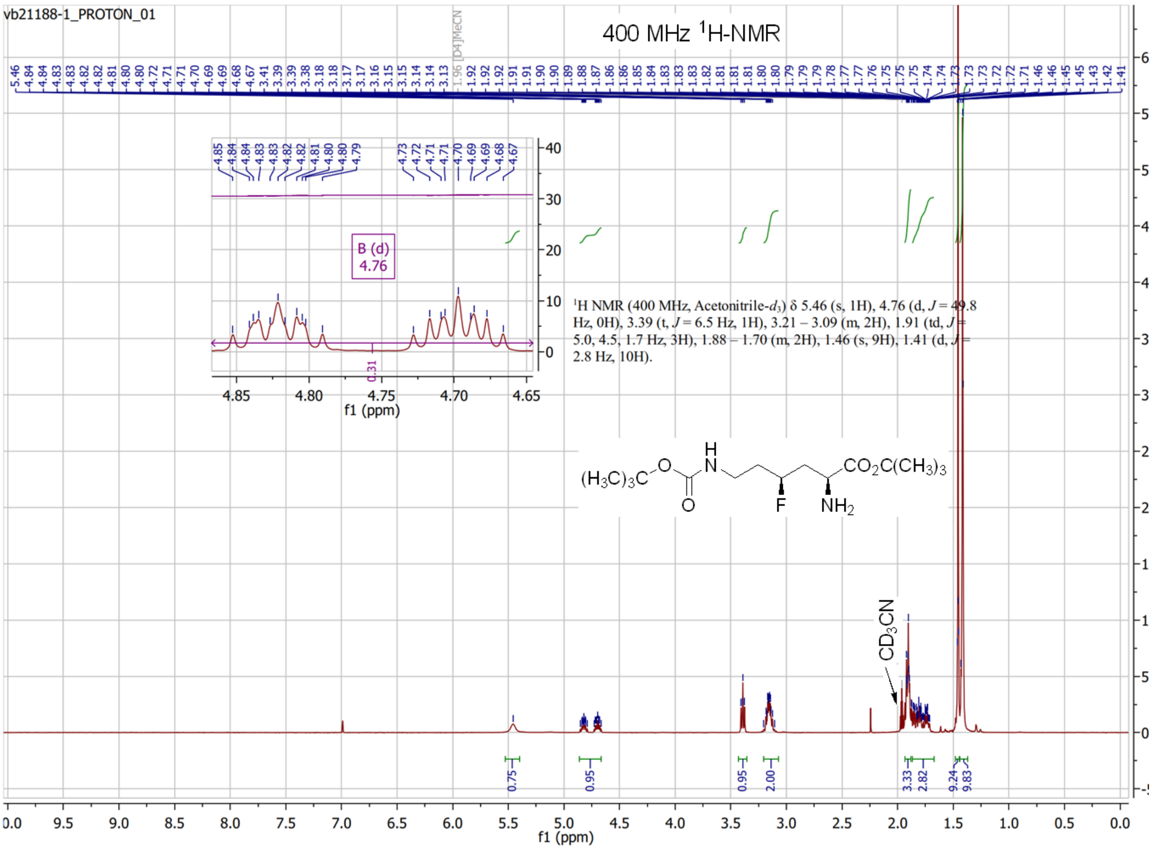

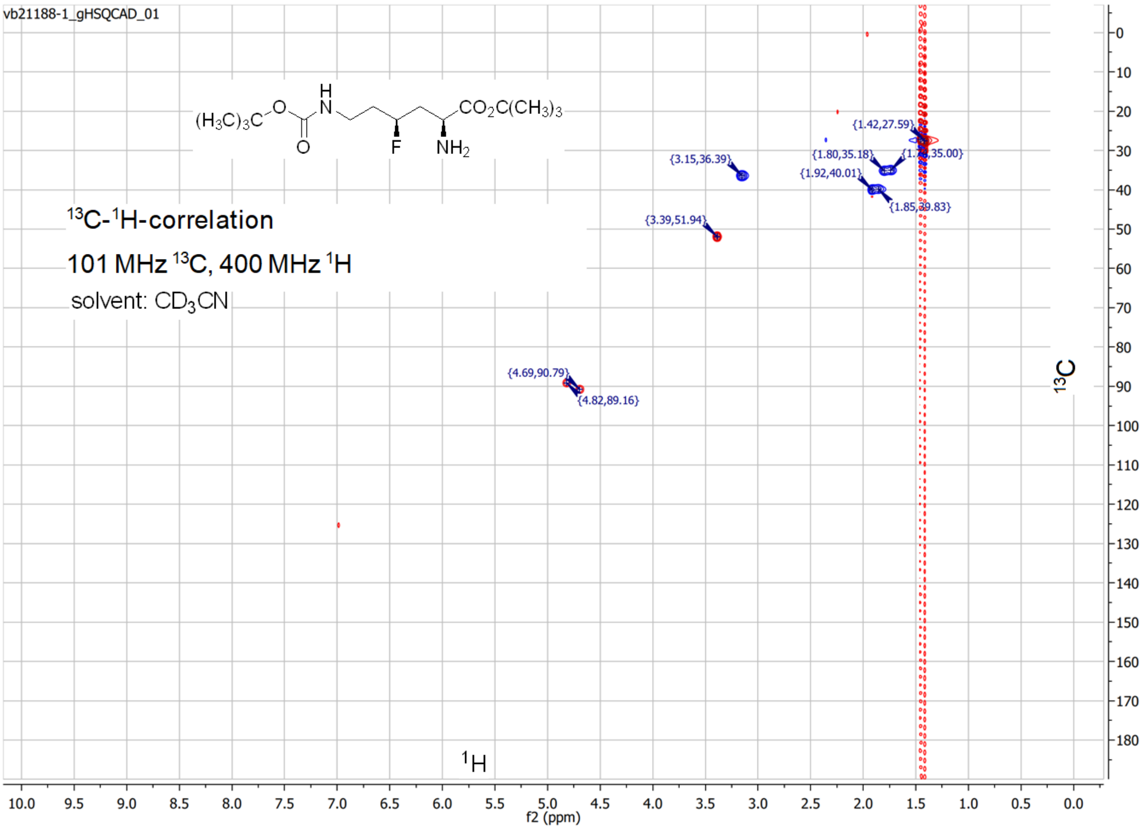

Compound 12 - epimer 2. (2*S*,4*R*)-BocNH$(CH_2)_2$CHFCH$_2$CH(NH$_2$)CO$_2$Bu^t, ^{1}H-NMR (400 MHz) and ^{13}C{^{1}H}-NMR (101 MHz) spectra in CD_3CN

^{1}H NMR (400 MHz, Acetonitrile-d_3) δ 5.39 (s, 1H), 4.90 (dddd, J = 10.0, 7.3, 4.4, 2.4 Hz, 0H), 4.78 (ddt, J = 12.3, 6.3, 2.4 Hz, 0H), 3.36 (dd, J = 10.3, 3.6 Hz, 1H), 3.16 (qd, J = 6.4, 2.4 Hz, 2H), 2.02 – 1.91 (m, 7H), 1.83 – 1.73 (m, 1H), 1.73 – 1.64 (m, 0H), 1.64 – 1.49 (m, 1H), 1.46 (d, J = 0.5 Hz, 12H), 1.42 (d, J = 3.1 Hz, 11H).

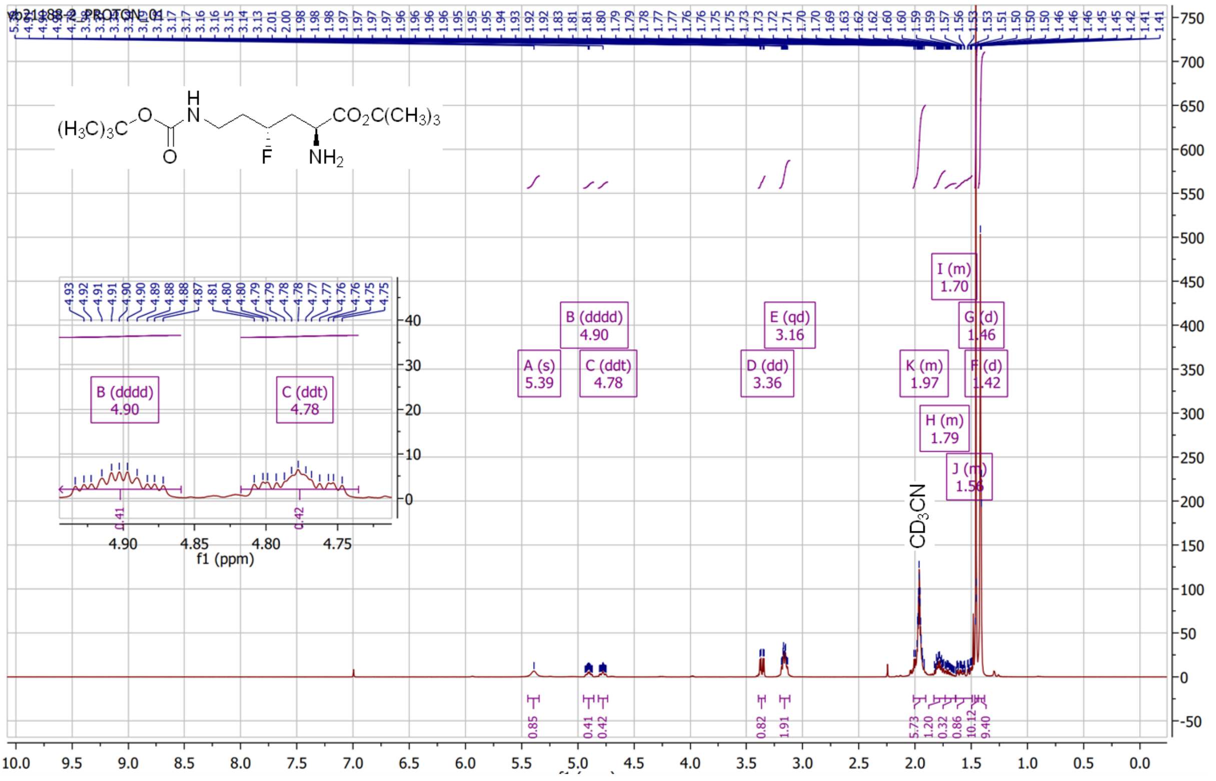

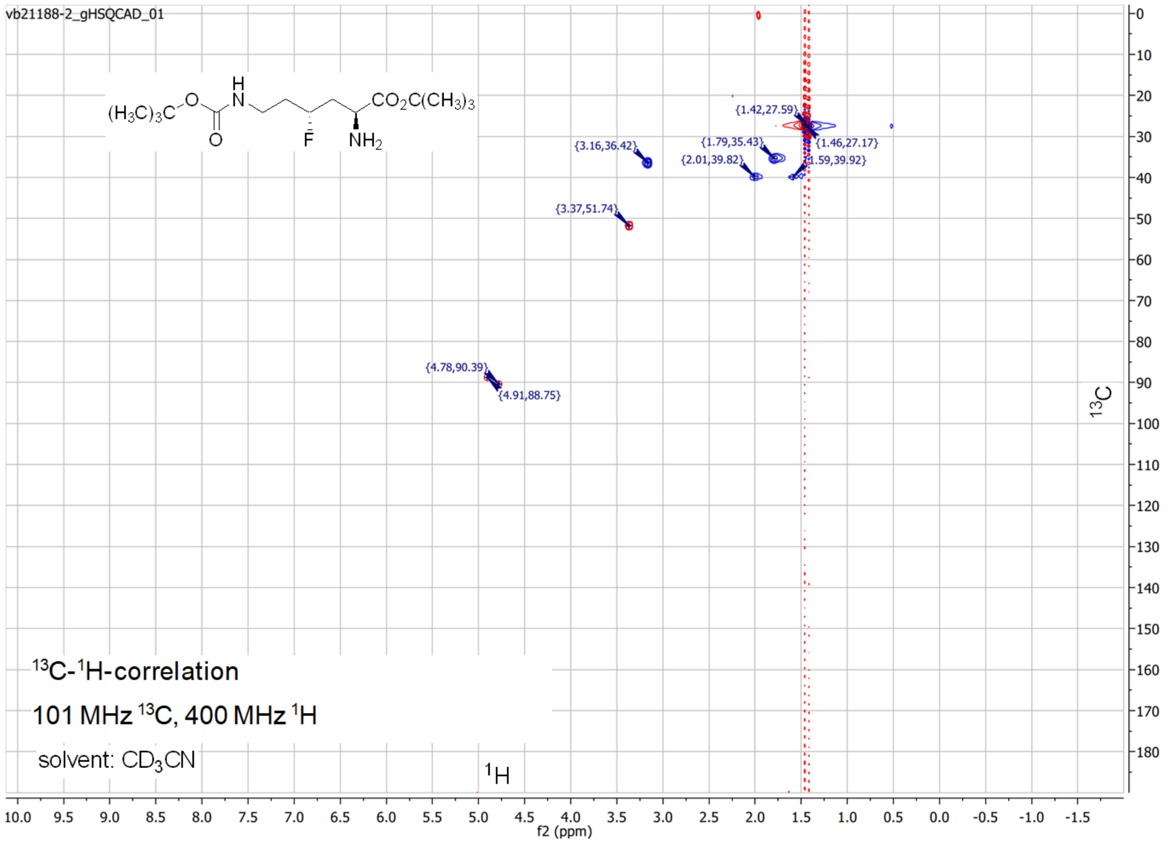

Compound 13 – epimer 1. (2*S*,4*S*)-BocNH$(CH_2)_2$CHFCH_2CH(NHFmoc)CO_2Bu^t.

Sample : vb21189-x

Gradient: A 20.0 % B 80.0 % ----> A 100.0 % B 0.0 % T = 7 Min.

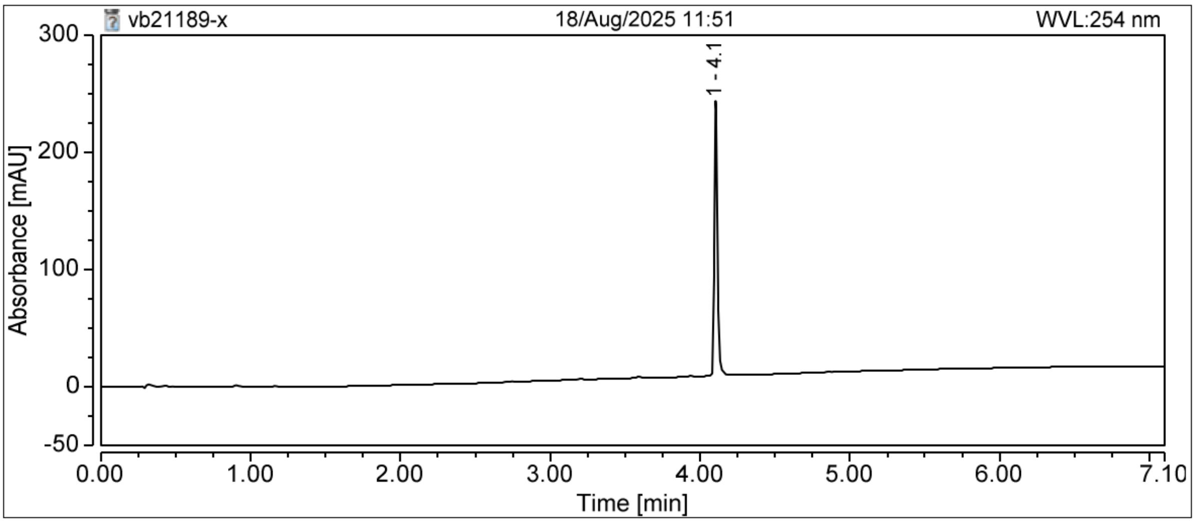

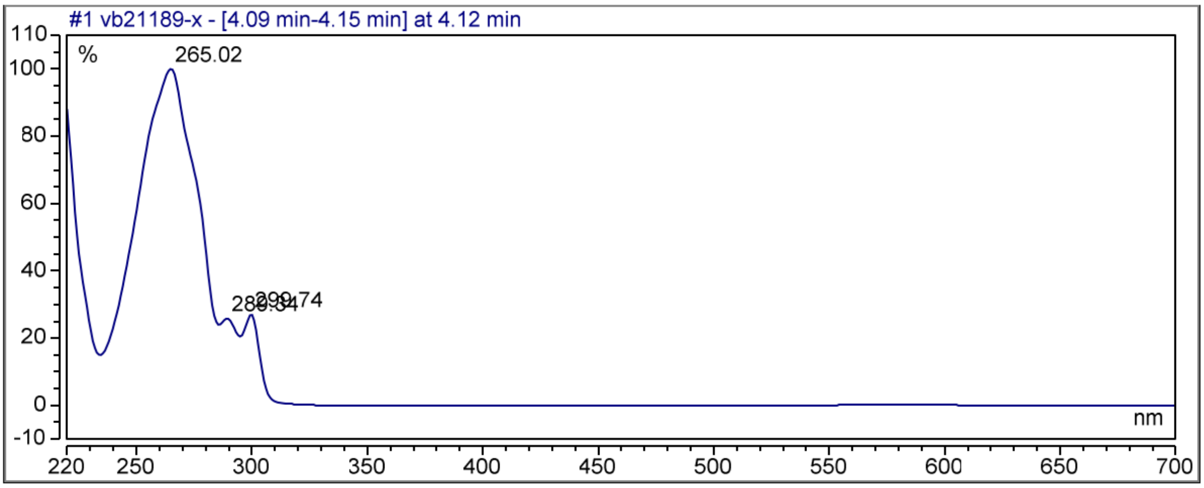

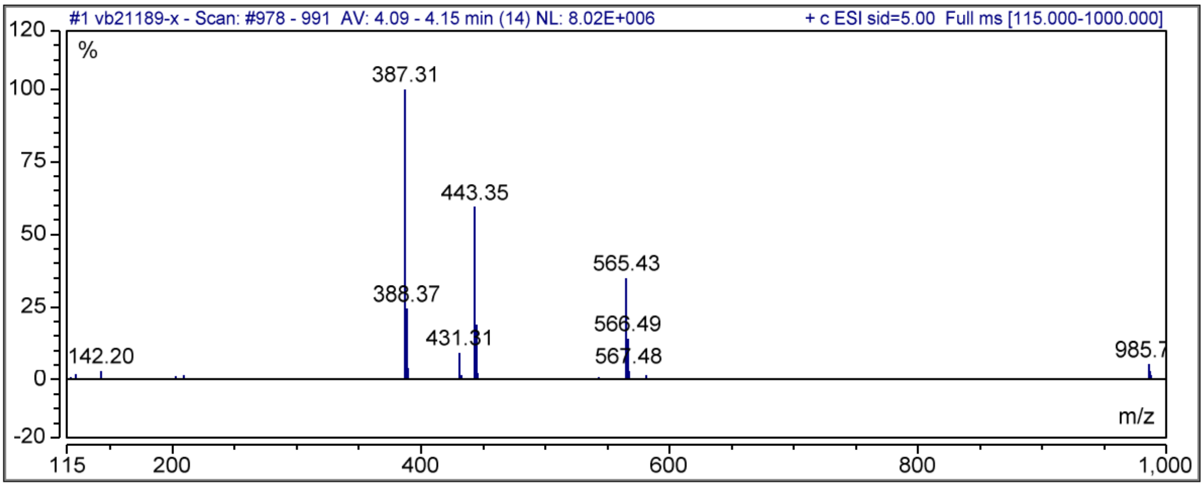

^{1}H-NMR (400 MHz), ^{13}C{^{1}H}-NMR (101 MHz) spectra in $CDCl_3$

^{1}H NMR (400 MHz, Chloroform-*d*) δ 7.78 (d, *J* = 7.5 Hz, 2H), 7.61 (d, *J* = 7.5 Hz, 2H), 7.41 (t, *J* = 7.5 Hz, 2H), 7.38 – 7.29 (m, 2H), 5.64 (d, *J* = 6.8 Hz, 1H), 4.80 (s, 0H), 4.76 – 4.64 (m, 2H), 4.41 (d, *J* = 7.1 Hz, 2H), 4.34 (t, *J* = 5.9 Hz, 1H), 4.23 (t, *J* = 6.9 Hz, 1H), 3.35 – 3.22 (m, 2H), 2.26 – 2.05 (m, 2H), 1.92 – 1.75 (m, 2H), 1.48 (s, 10H), 1.44 (s, 9H).

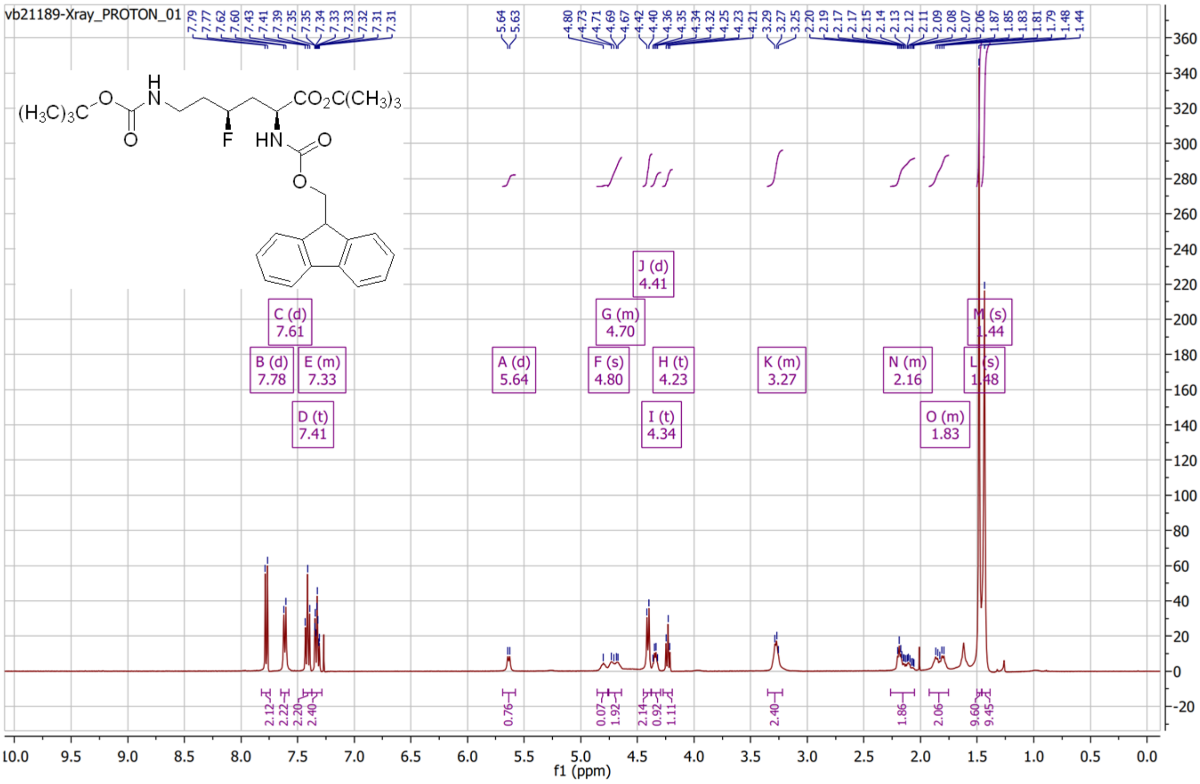

^{13}C NMR (101 MHz, Chloroform-*d*) δ 89.40 (d, *J* = 166.6 Hz), 37.59 (d, *J* = 20.5 Hz), 35.24 (d, *J* = 20.1 Hz).

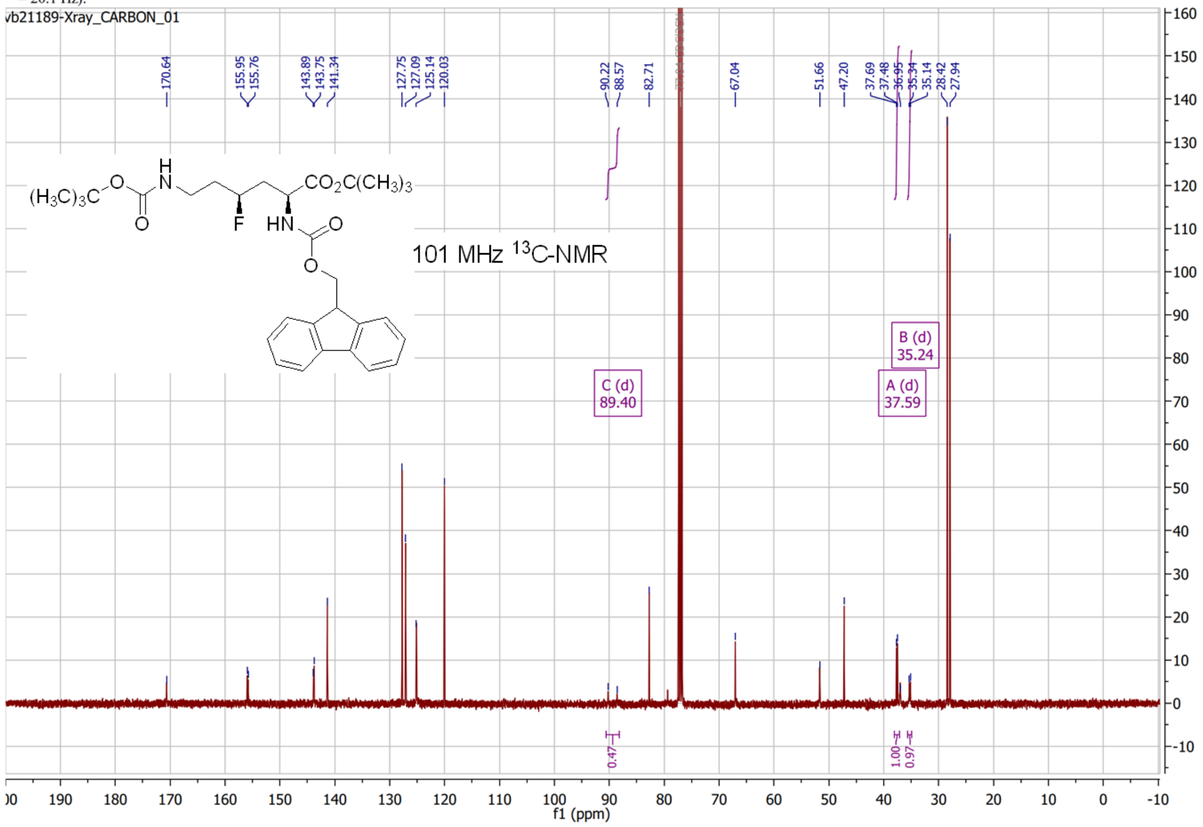

Compound 14 – epimer 1. (2*S*,4*S*)-$NH_2(CH_2)_2CHFCH_2CH(NHFmoc)CO_2H*CF_3CO_2H$

Sample : **vb21190-1**

Gradient: A 20.0 % B 80.0 % ----> A 100.0 % B 0.0 % T = 7 Min.

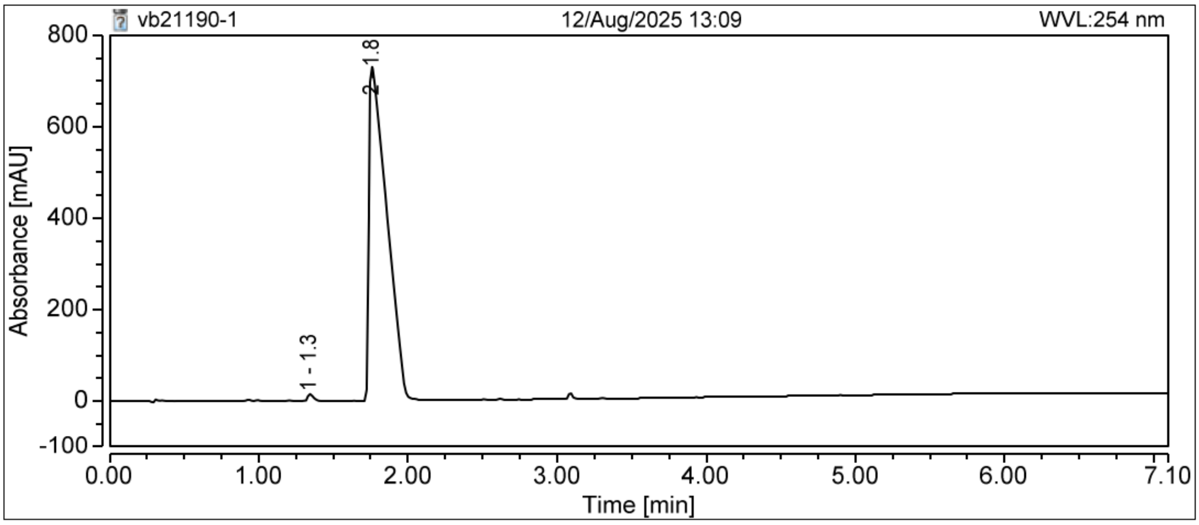

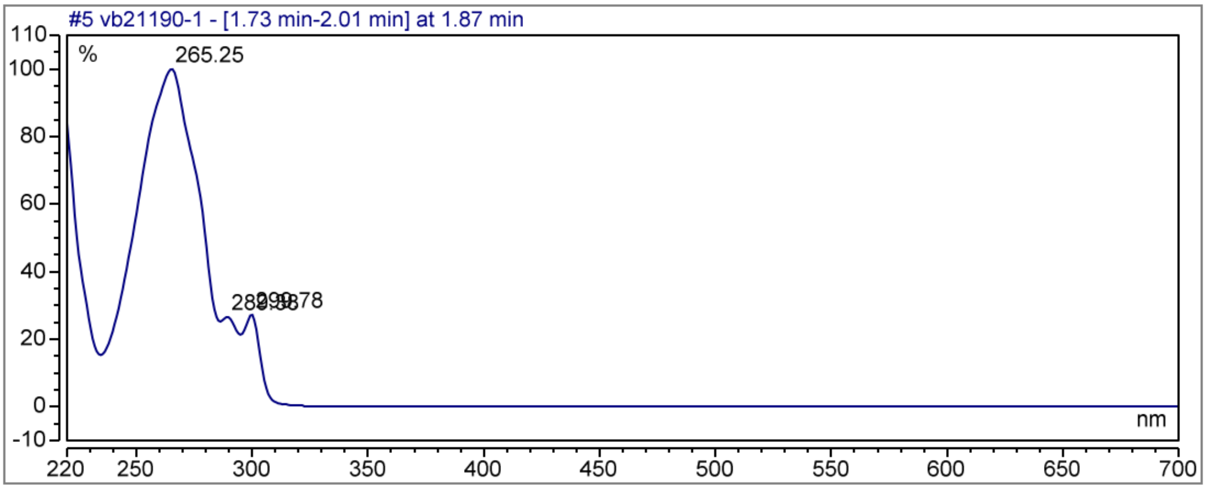

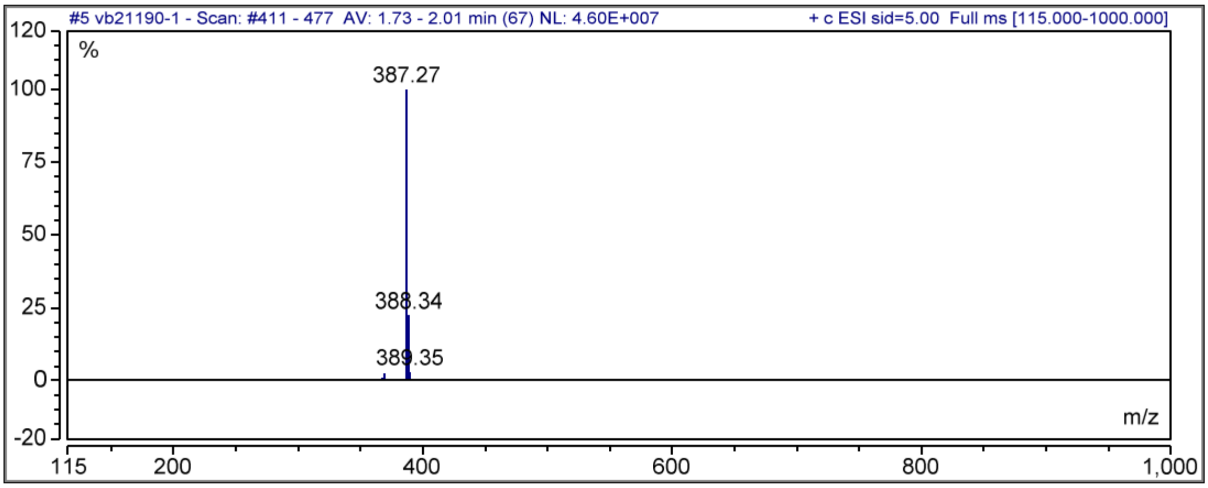

^{1}H-NMR (400 MHz) and $^{13}C\{^{1}H\}$-NMR (101 MHz) spectra in CD_3CN

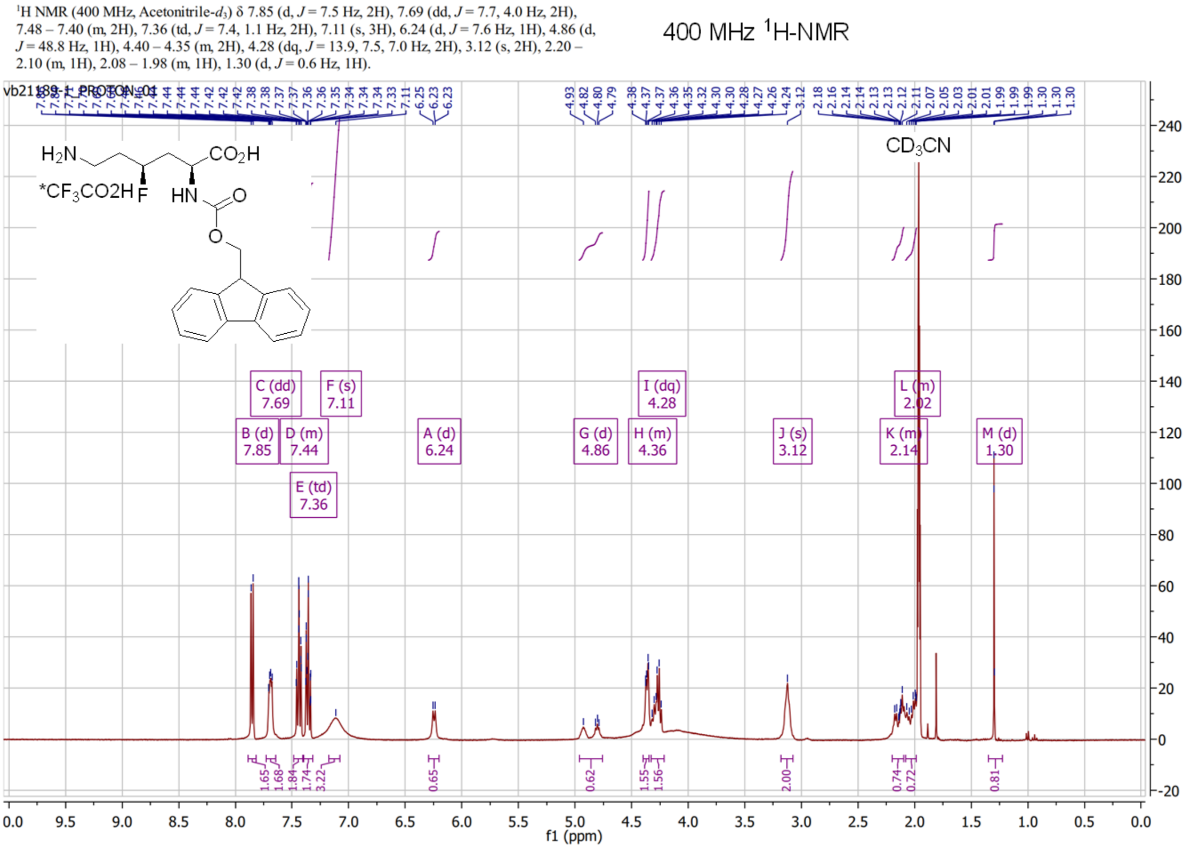

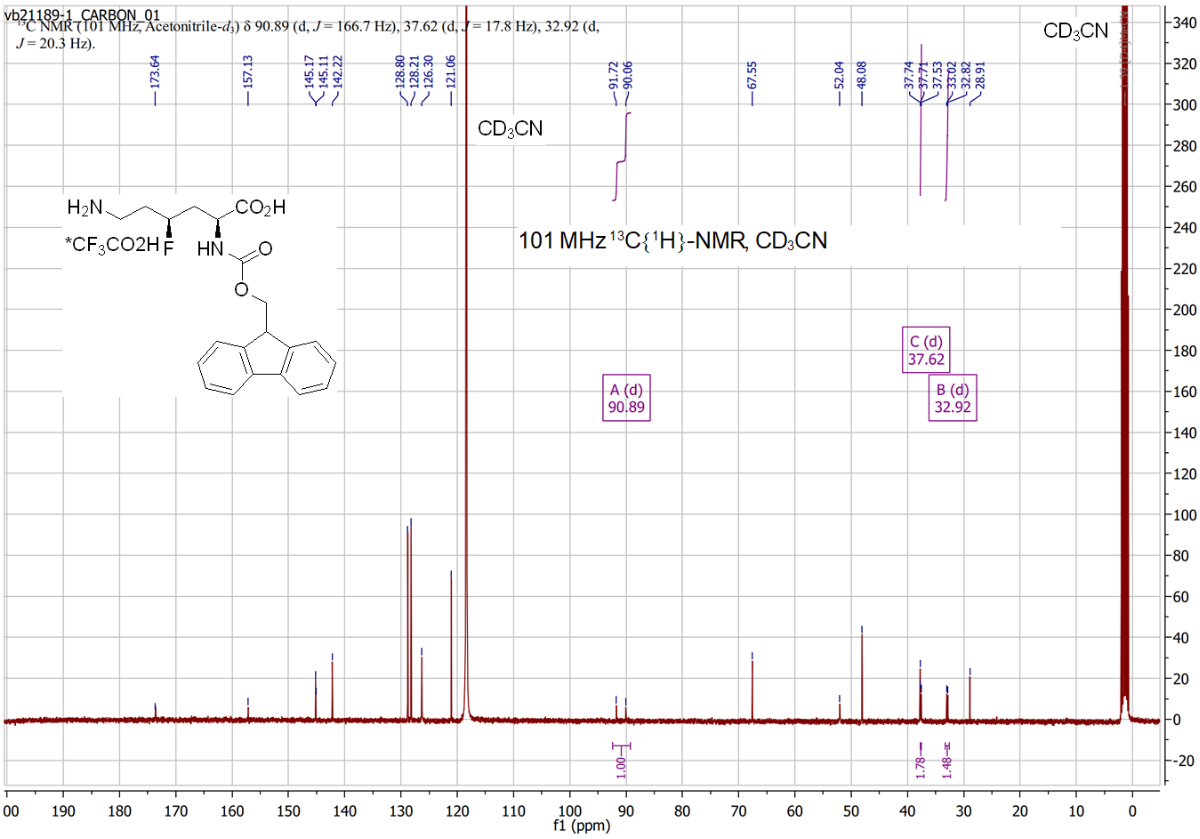

Compound 14 – epimer 2. (2*S*,4*R*)-$NH_2(CH_2)_2CHFCH_2CH(NHFmoc)CO_2H*CF_3CO_2H$

Sample : **vb21190-2**

Gradient: A 20.0 % B 80.0 % **---->** A 100.0 % B 0.0 % T = 7 Min.

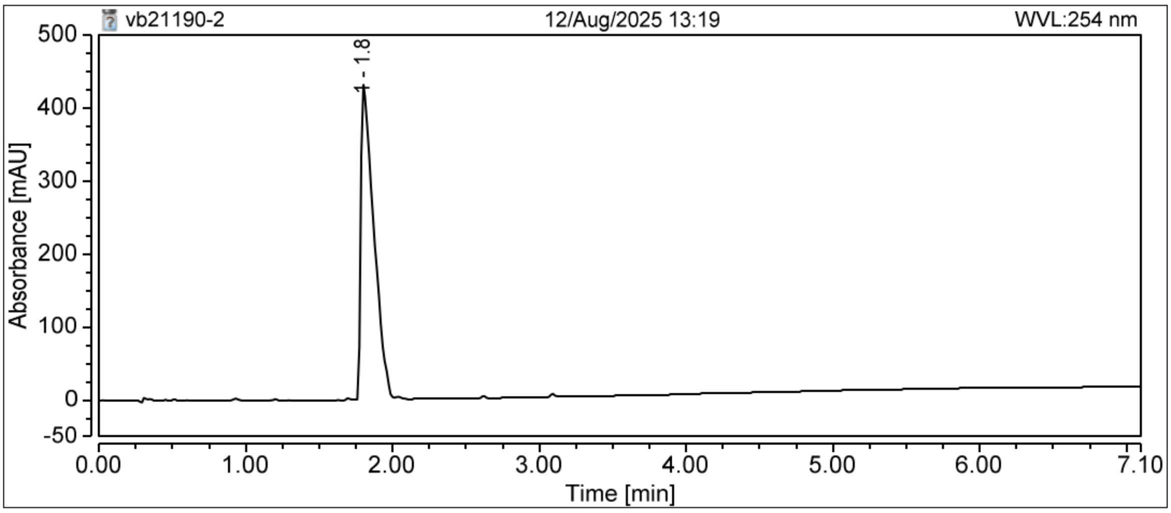

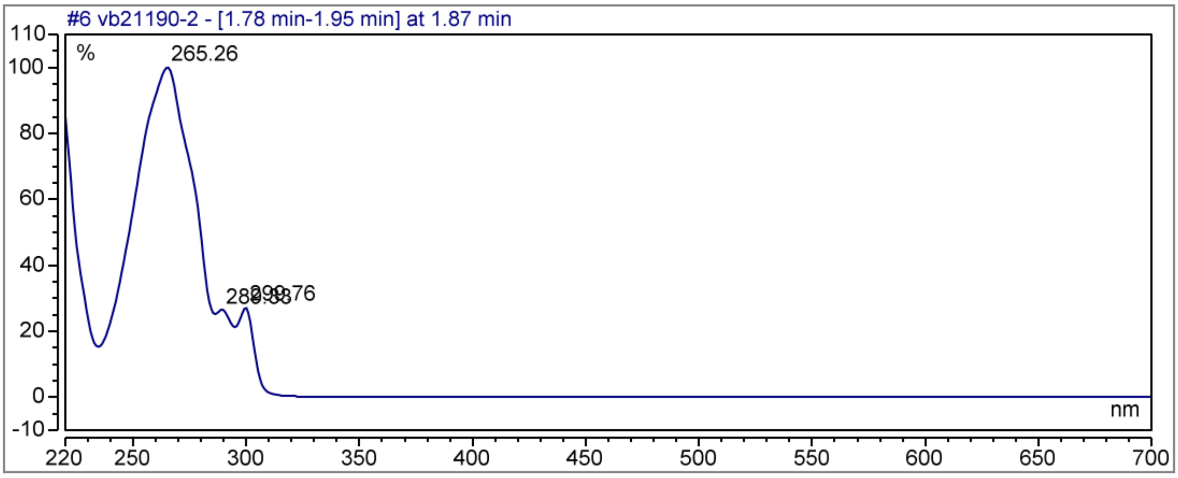

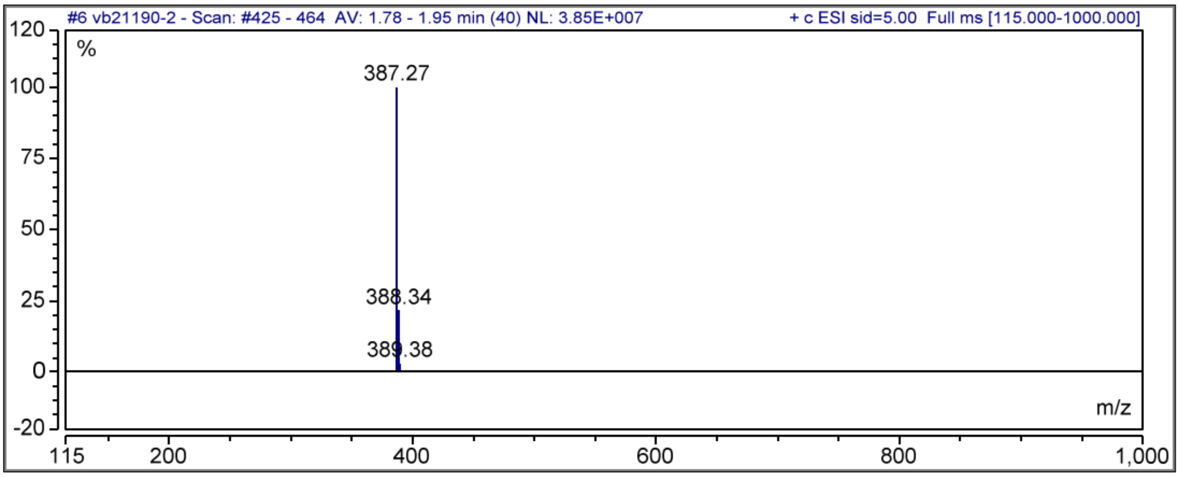

^{1}H-NMR (400 MHz) and $^{13}C\{^{1}H\}$-NMR (101 MHz) spectra in CD_3CN

^{1}H NMR (400 MHz, Acetonitrile-d_3) δ 7.82 (d, J = 7.5 Hz, 2H), 7.68 – 7.63 (m, 2H), 7.59 (s, 1H), 7.41 (t, J = 7.5 Hz, 2H), 7.37 – 7.29 (m, 2H), 4.68 (d, J = 48.5 Hz, 1H), 4.40 – 4.31 (m, 2H), 4.25 (dt, J = 13.7, 7.0 Hz, 2H), 3.06 (t, J = 7.7 Hz, 3H), 2.19 (q, J = 14.4 Hz, 1H), 1.92 – 1.78 (m, 1H), 1.28 – 1.18 (m, 1H).

400 MHz ^{1}H-NMR, CD_3CN

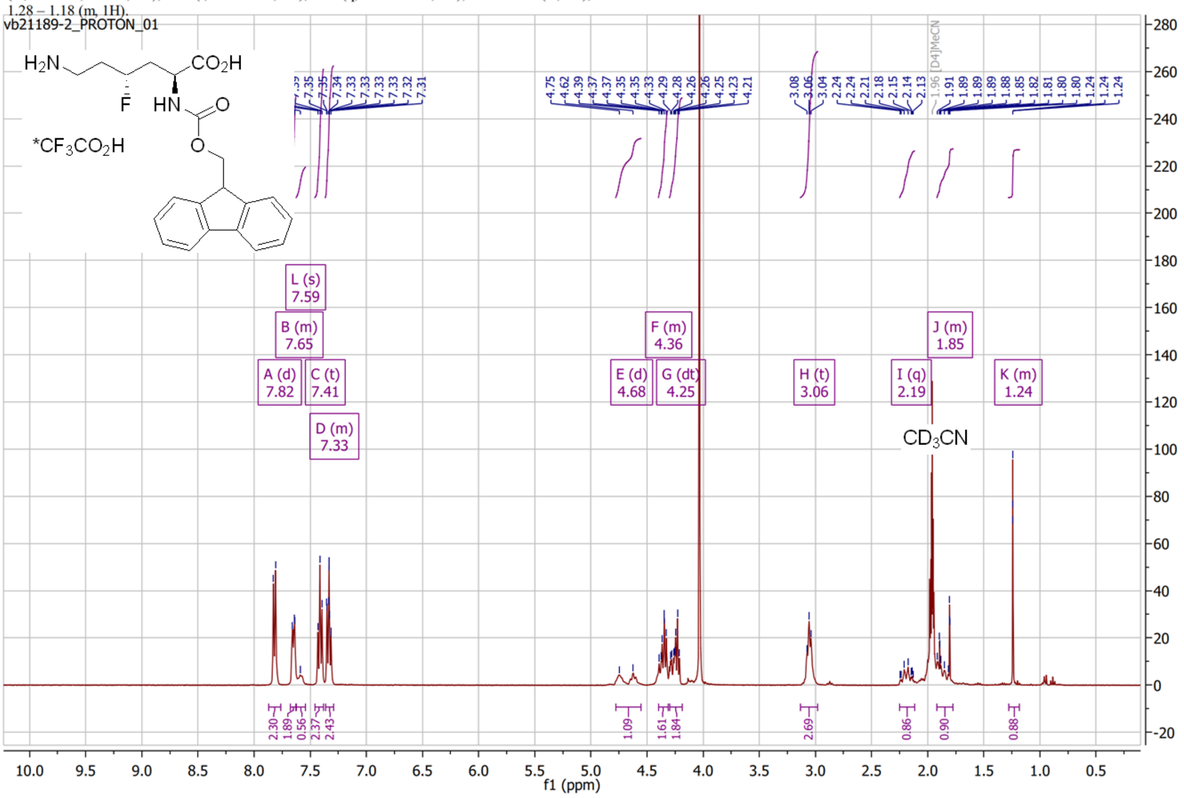

^{13}C NMR (101 MHz, Acetonitrile-d_3) δ 90.33 (d, J = 166.9 Hz), 37.05 (d, J = 21.1 Hz), 32.96 (d, J = 20.3 Hz).

101 MHz ^{13}C-NMR, CD_3CN

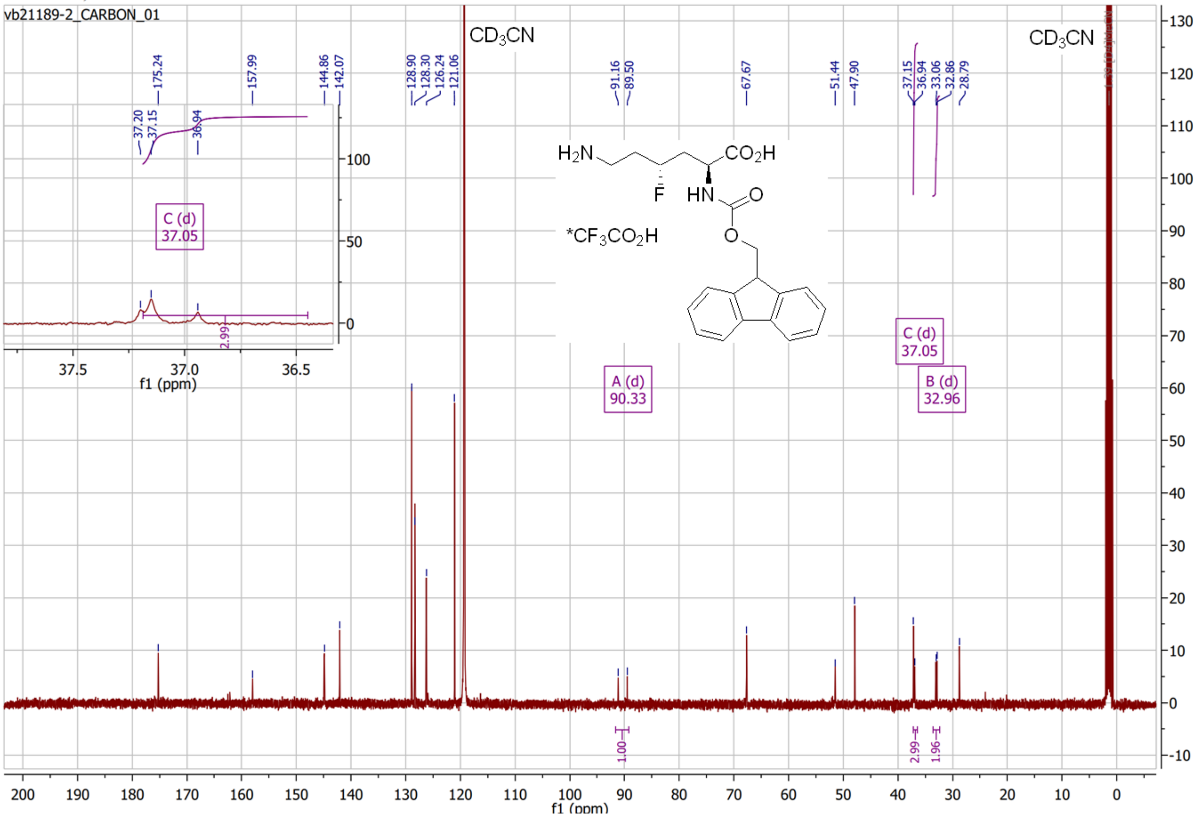

Compound 15 – epimer 1. (2*S*,4*S*)-BocNH$(CH_2)_2$CHFCH_2CH(NHFmoc)CO_2H

Sample : **vb21191-1**

Gradient: A 20.0 % B 80.0 % ----> A 100.0 % B 0.0 % T = 7 Min.

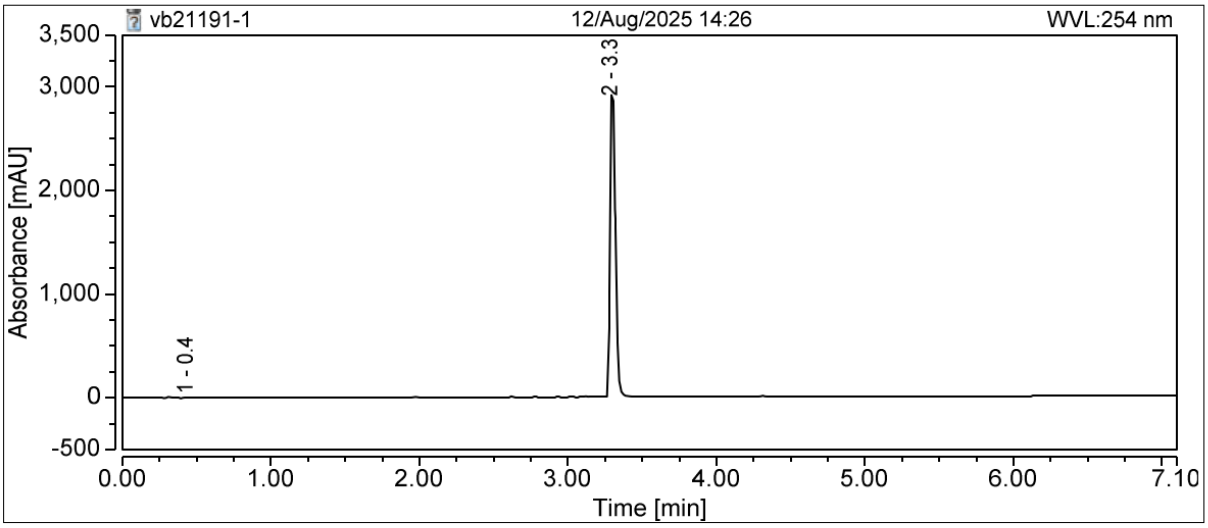

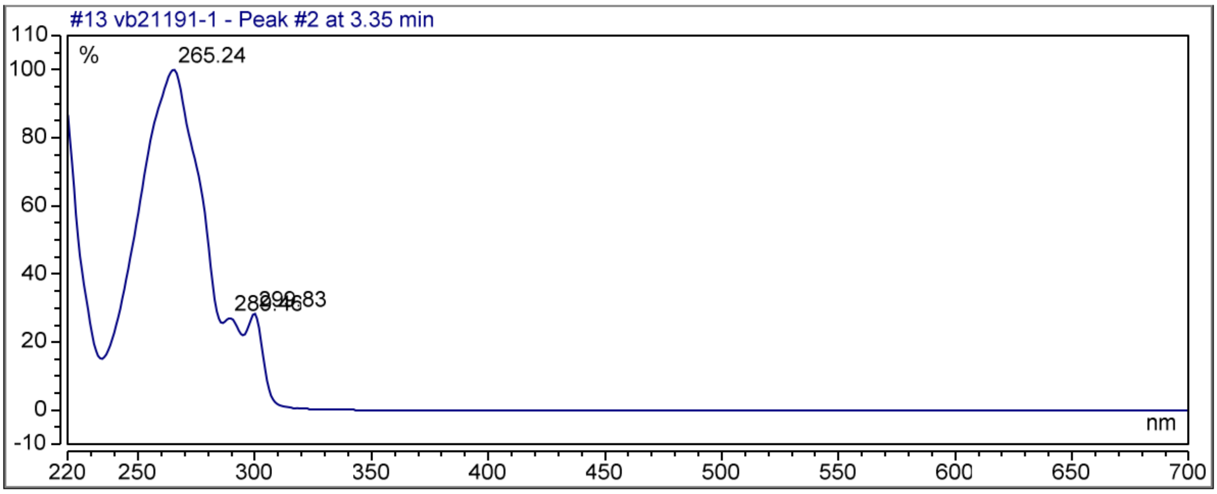

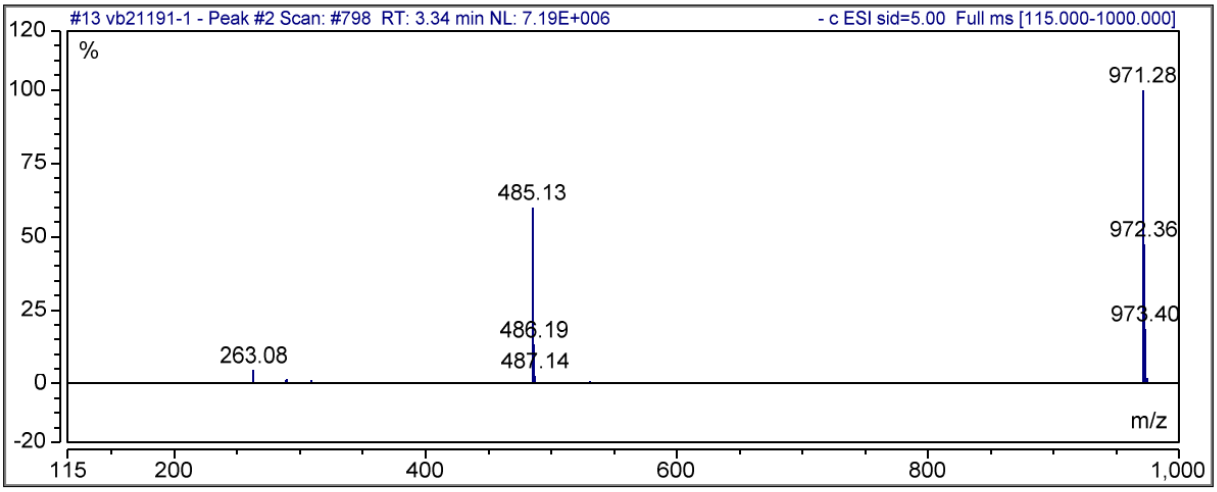

^{1}H-NMR (400 MHz) and $^{13}C\{^{1}H\}$-NMR (101 MHz) spectra in CD_3OD

^{1}H NMR (400 MHz, Methanol-d_4) δ 7.81 – 7.75 (m, 2H), 7.67 (ddd, J = 6.4, 5.0, 2.6 Hz, 2H), 7.40 – 7.35 (m, 2H), 7.33 – 7.27 (m, 2H), 4.65 (d, J = 51.7 Hz, 1H), 4.33 (dd, J = 7.1, 3.7 Hz, 2H), 4.26 – 4.14 (m, 2H), 3.17 (t, J = 6.9 Hz, 2H), 2.26 – 2.10 (m, 1H), 1.87 – 1.64 (m, 3H), 1.41 (d, J = 6.8 Hz, 9H).

400 MHz ^{1}H-NMR, CD_3OD

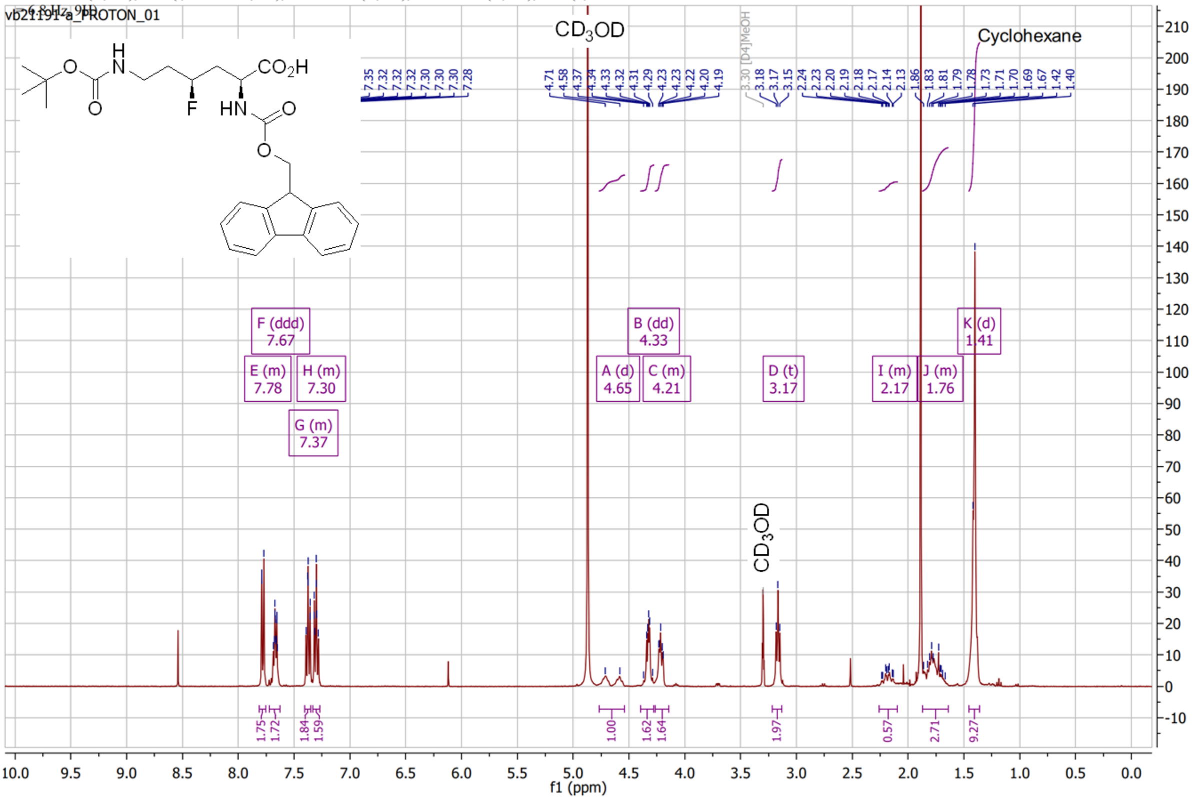

^{13}C NMR (101 MHz, Methanol-d_4) δ 90.67 (d, J = 168.2 Hz), 39.64 (d, J = 20.9 Hz), 36.76 (d, J = 20.6 Hz).

101 MHz ^{13}C-NMR, CD_3OD

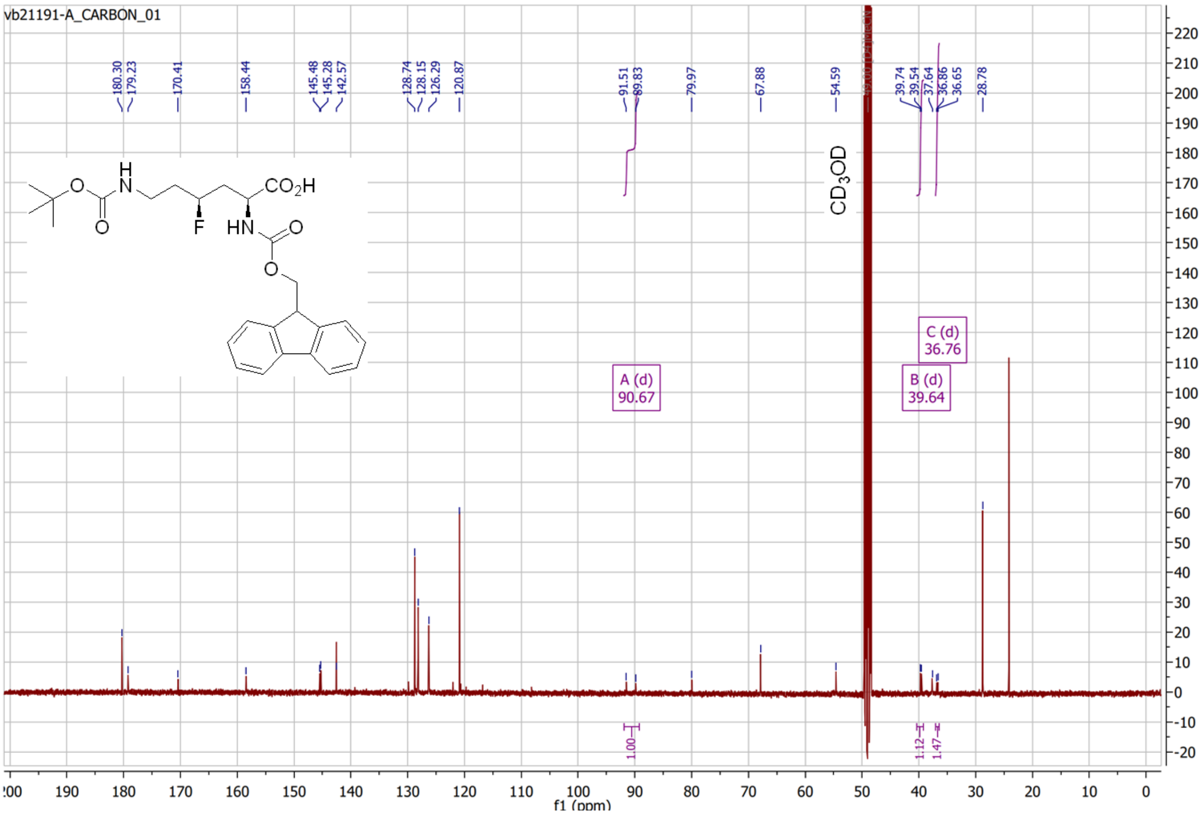

Compound 15 – epimer 2. (2*S*,4*R*)-BocNH$(CH_2)_2$CHFCH_2CH(NHFmoc)CO_2H

Sample : **vb21191-2**

Gradient: A 20.0 % B 80.0 % ----> A 100.0 % B 0.0 % T = 7 Min.

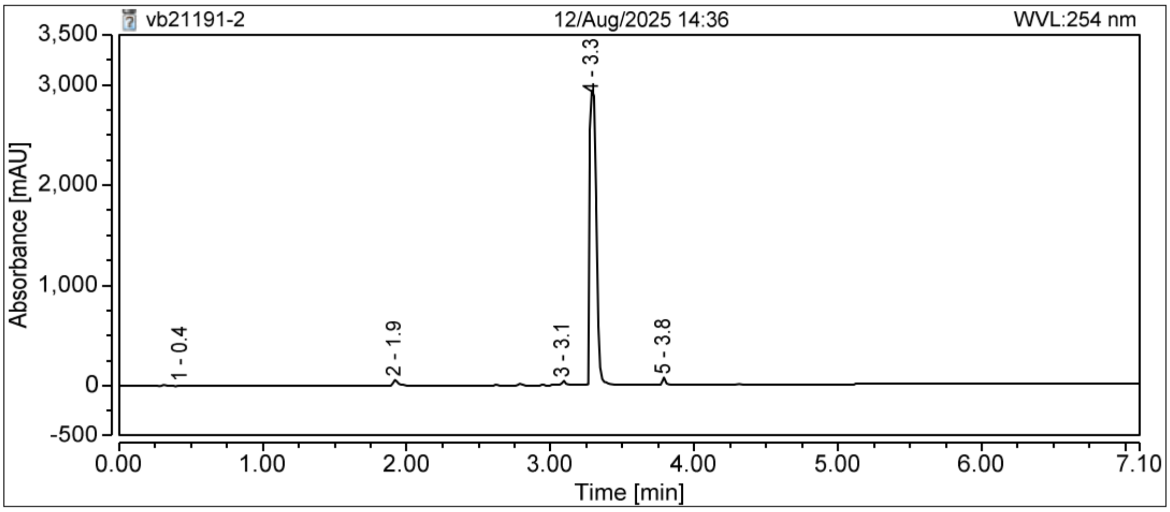

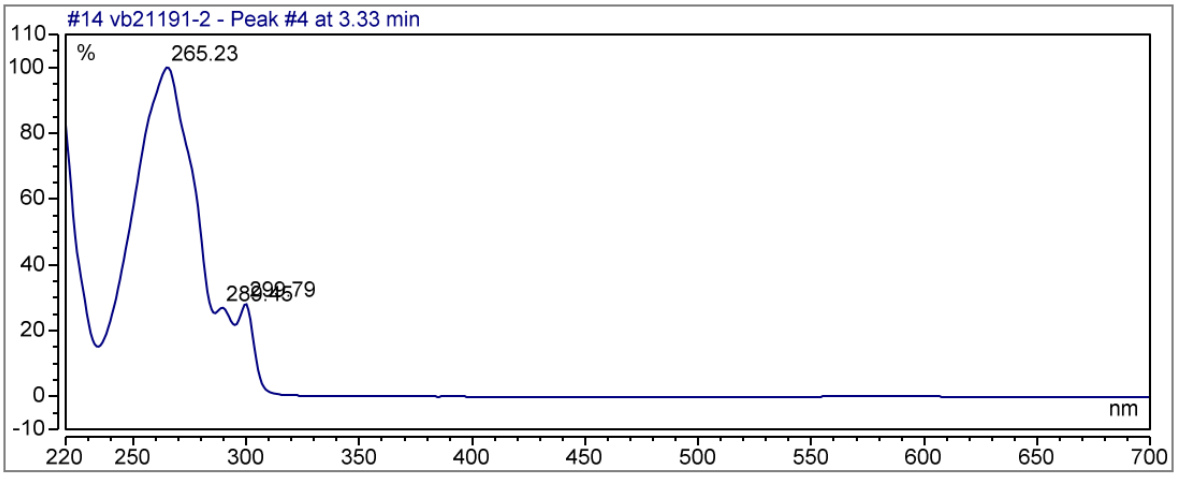

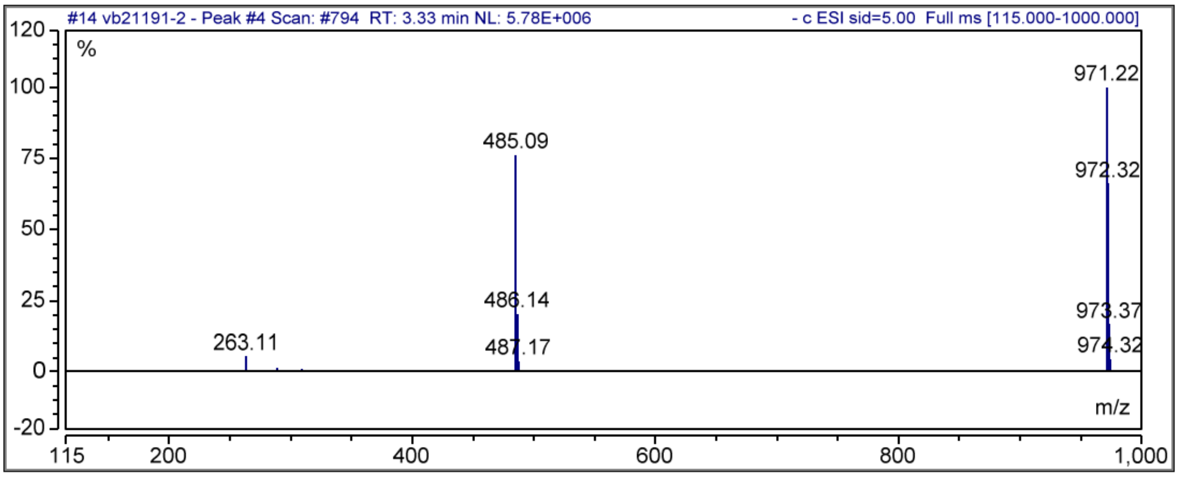

^{1}H-NMR (400 MHz) and ^{13}C{^{1}H}-NMR (101 MHz) spectra in CD_3OD

^{1}H NMR (400 MHz, Methanol-d_4) δ 7.79 (dt, J = 7.6, 0.9 Hz, 3H), 7.73 – 7.63 (m, 3H), 7.43 – 7.35 (m, 3H), 7.31 (td, J = 7.5, 1.2 Hz, 3H), 4.71 (dd, J = 48.9, 6.1 Hz, 1H), 4.34 (dd, J = 7.0, 3.8 Hz, 2H), 4.22 (t, J = 7.0 Hz, 1H), 4.13 (t, J = 6.7 Hz, 1H), 3.21 – 3.14 (m, 2H), 2.13 – 1.99 (m, 2H), 1.83 – 1.70 (m, 2H), 1.39 (s, 9H).

400 MHz ^{1}H-NMR, CD_3OD

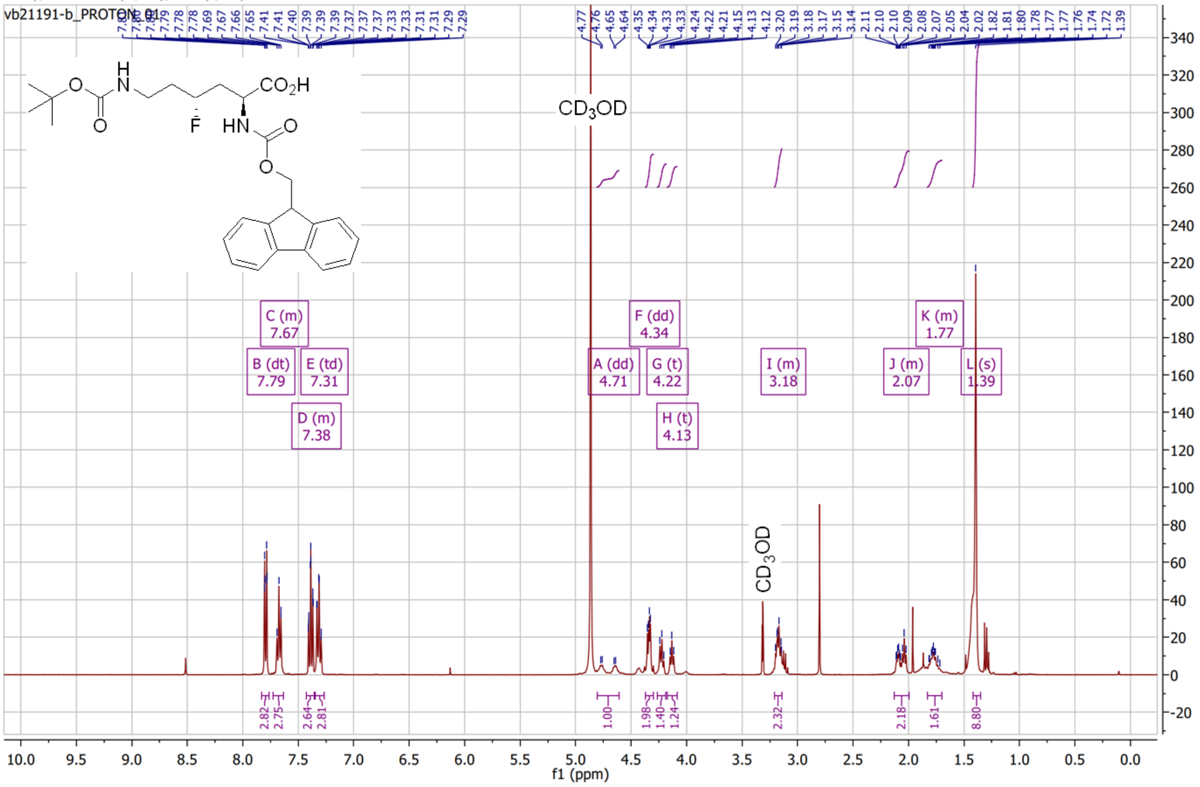

^{13}C NMR (101 MHz, Methanol-d_4) δ 91.11 (d, J = 167.3 Hz), 39.69 (d, J = 21.0 Hz), 36.23 (d, J = 21.2 Hz).

101 MHz ^{13}C-NMR, CD_3OD

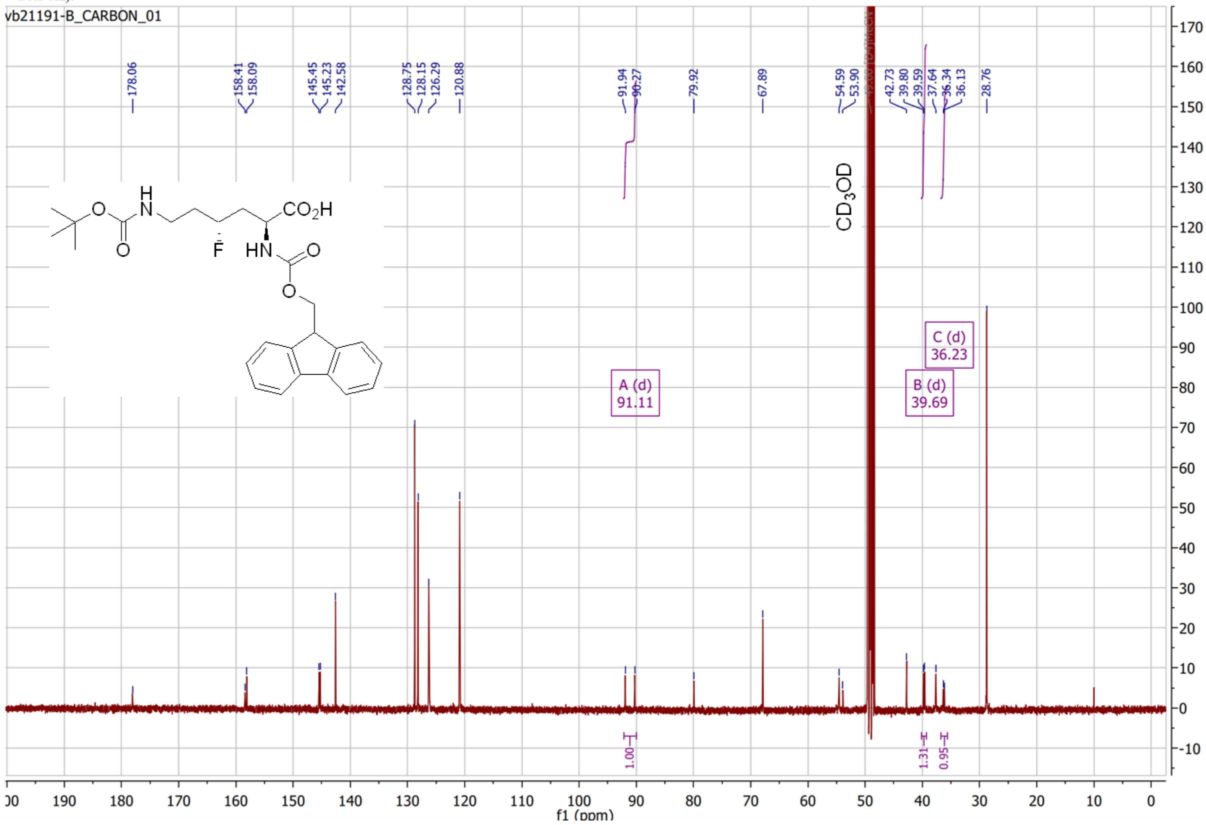

Crystallographic Data

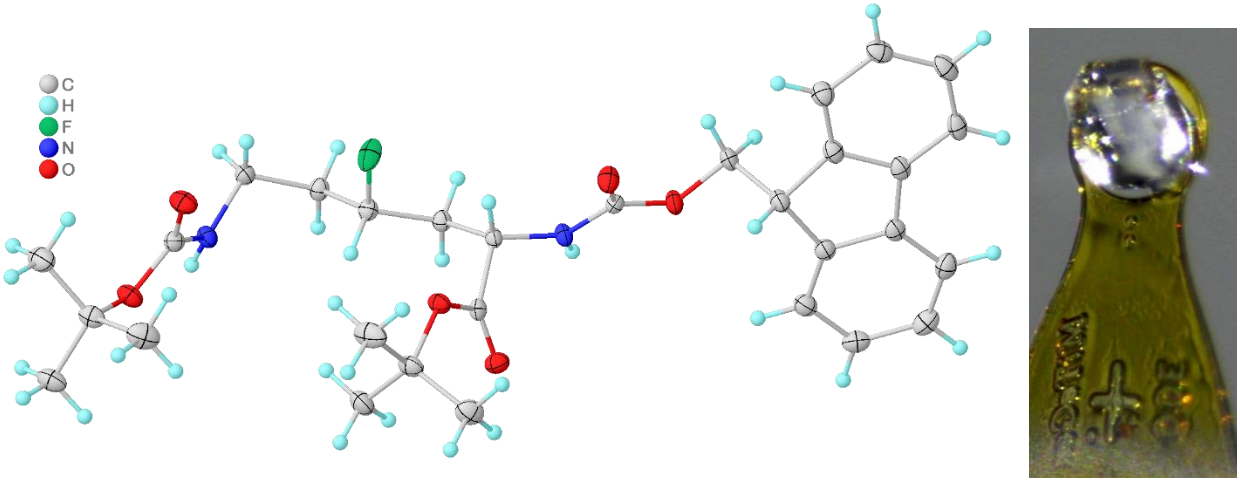

Figure S 1: Asymmetric unit of (4*S*)-**13**-epimer 1. Anisotropic displacement ellipsoids drawn at 50% probability level. Single-crystals were obtained from a solution in acetonitrile.

CCDC number	2485223
Empirical formula	$C_{30}H_{39}FN_2O_6$
Formula weight	542.63
Temperature [K]	100.00
Crystal system	Orthorhombic
Space group (number)	$P2_12_12_1$ (19)
a [Å]	8.9396(7)
b [Å]	9.5781(9)
c [Å]	33.592(4)
α [°]	90
β [°]	90
γ [°]	90
Volume [Å^3]	2876.3(5)
Z	4
ρ_{calc} [gcm^{-3}]	1.253
μ [mm^{-1}]	0.750
F(000)	1160
Crystal size [mm^3]	0.177×0.28×0.342
Crystal color	Colorless
Crystal shape	Block
Radiation	CuK_α (λ=1.54178 Å)
2θ range [°]	5.26 to 158.59 (0.78 Å)

Index ranges	−11 ≤ h ≤ 11<br>−11 ≤ k ≤ 12<br>−42 ≤ l ≤ 42
Reflections collected	85222
Independent reflections	6186<br>R_{int} = 0.0398<br>R_{sigma} = 0.0145
Completeness to θ = 67.679°	99.9 %
Data / Restraints / Parameters	6186 / 0 / 367
Absorption correction T_{min}/T_{max} (method)	0.7738 / 0.9470 (numerical)
Goodness-of-fit on F^2	1.042
Final R indexes [I≥2σ(I)]	R_1 = 0.0262<br>wR_2 = 0.0691
Final R indexes [all data]	R_1 = 0.0263<br>wR_2 = 0.0692
Largest peak/hole [eÅ^{-3}]	0.22/−0.17
Extinction coefficient	0.0026(2)
Flack X parameter	0.00(2)
